# Investigating the Impact of Circulating MicroRNAs on Knee and Hip Osteoarthritis: Causal Links, Biological Mechanisms, and Drug Interactions

**DOI:** 10.3390/ijms26010283

**Published:** 2024-12-31

**Authors:** Shanni Li, Yihui Peng, Yang Yu, Hongjun Xu, Zhaojing Yin, Yiyang Du, Mingyang Ma, Zhongyin Ji, Wenwei Qian

**Affiliations:** 1 Peking Union Medical College, Chinese Academy of Medical Science, Beijing 100010, China; lishanni.pumch@gmail.com (S.L.); gkyy1998@163.com (Y.Y.); xuhj1998@163.com (H.X.); mingyangyang1@163.com (M.M.); zyji@zju.edu.cn (Z.J.); 2 College of Computer Science and Software Engineering, Shenzhen University, Shenzhen 518060, China; yihuipengsms@gmail.com; 3 School of Medicine, Tsinghua University, Beijing 100084, China; yin-zj20@mails.tsinghua.edu.cn (Z.Y.); yy-du20@student.pumc.edu.cn (Y.D.)

**Keywords:** osteoarthritis, microRNAs, mendelian randomization, drug targets, signaling pathways, biological mechanisms

## Abstract

Osteoarthritis (OA), particularly in the knee and hip, poses a significant global health challenge due to limited therapeutic options. To elucidate the molecular mechanisms of OA and identify potential biomarkers and therapeutic targets, we utilized genome-wide association studies (GWAS) and cis-miRNA expression quantitative trait loci (cis-miR-eQTL) datasets to identify miRNAs associated with OA, revealing 16 that were linked to knee OA and 21 to hip OA. Among these, hsa-miR-1303 was significantly upregulated in both knee and hip OA (IVW: *p* = $6.8164\times 10^{-36}$ and $4.7919\times 10^{-2}$ respectively, OR > 1) and identified as a key factor in disease progression. Hsa-miR-1303 potentially regulates 30 genes involved in critical signaling pathways, such as the neurotrophin signaling pathway, and interacts with competing endogenous RNAs (ceRNAs) like circ_0041843 and LINC01338, thereby influencing key regulatory proteins such as SUMO2 and PARP1. Pharmacologically, hsa-miR-1303 targets nine druggable genes, including NRAS, H2AZ1, and RPS3, which have implications for drugs like cantharidin and diindolylmethane, potentially critical for developing novel OA treatments. Conversely, hsa-miR-125a-5p and hsa-miR-125b-5p, which are downregulated in both knee and hip OA, are associated with pathways such as HIF-1 and JAK-STAT, which modulate apoptotic signaling and transcriptional regulation. These miRNAs also interact with ceRNAs such as circ_0000254 and SPACA6P-AS, impacting proteins like STAT3, MCL1, and TRAF6. A drug interaction analysis identified 47 potential treatments, including Resveratrol and Acetaminophen, suggesting new therapeutic possibilities for OA management. This study not only highlights the role of miRNAs like hsa-miR-1303 and hsa-miR-125 in OA but also opens avenues for miRNA-based therapeutic development.

## 1. Introduction

Osteoarthritis (OA) is the most prevalent form of arthritis. It affects millions globally and serves as a leading cause of chronic pain and long-term disability among adults [1]. In 2020, 595 million people had osteoarthritis worldwide, which equals to 7.6% of the global population and an increase of 132.2% in total cases since 1990 [2]. However, there are neither available biomarkers for early diagnosis of the disease nor any effective therapy other than symptomatic treatment and joint replacement surgery [3]. MicroRNAs (miRNAs) are small non-coding RNAs that regulate gene expression and play critical roles in various physiological processes, including cell differentiation, inflammation, and tissue repair, all relevant to OA [4]. Recent studies have revealed that these miRNAs, found in bodily fluids like blood, urine, serum, plasma, and cerebrospinal fluid, are chemically stable [5], which makes them promising non-invasive biomarkers for disease diagnosis, prognosis, and treatment efficacy monitoring.

Current research on miRNA-related drug therapies is showing significant progress, with promising candidates such as Miravirsen for Hepatitis C [6], Cobomarsen for certain lymphomas [7], and Remlarsen aimed at reducing skin scarring [8] and currently undergoing clinical trials. Yet, miRNA research for osteoarthritis (OA) remains primarily at the experimental stage. Murata et al. found that miR-16 and miR-132 levels are significantly lower in the plasma of OA patients [9]. Borgonio Cuadra et al. identified 12 miRNAs with elevated expression levels in the plasma of OA patients, including miR-16, miR-20b, miR-29c, miR-30b, miR-93, miR-126, miR-146a, miR-184, miR-186, miR-195, miR-345, and miR-885-5p [10]. Moreover, recent studies have further revealed the extensive roles of miRNAs in osteoarthritis, particularly in crucial pathways such as MMP13 [11], STAT3 [12], SMAD2/3 [13], NOTCH3/Notch [14], and NF-κB signaling [15]. However, due to inherent limitations in traditional research designs, these studies could not completely rule out the risk of reverse causality or the influence of confounding factors such as obesity, diabetes, cardiovascular diseases, joint injuries, and physical activity, which could lead to biased results and conclusions [16]. Additionally, these findings typically indicate associations rather than causations, which compromise the reliability of miRNAs as OA biomarkers.

Mendelian Randomization (MR) is a robust method that uses genetic variants as instrumental variables (IVs) to estimate the causal effects of exposures on outcomes. This method helps reduce confounding biases because genetic variants are assigned at conception, and it also prevents reverse causation since these variants exist before the disease develops [17,18]. To evaluate the potential of miRNAs in predicting and treating OA, we employed MR analyses to investigate the causal impacts of specific miRNAs on the risk and progression of osteoarthritis, as well as to explore the potential mechanisms of these risk and protective miRNAs, using bioinformatics tools. Our research elucidates these causal relationships to better understand the molecular foundations of OA and to promote the development of innovative miRNA-based therapeutic strategies. This could lead to more precise and effective diagnoses and interventions, ultimately improving outcomes for osteoarthritis patients.

## 2. Results

After applying rigorous inclusion criteria and conducting detailed statistical analyses, we identified 16 miRNAs causally connected to knee osteoarthritis and 21 to hip osteoarthritis. Among these, hsa-miR-1303 was upregulated in both knee and hip OA patients (IVW: OR > 1, *p* < 0.05), while hsa-miR-125a-5p and hsa-miR-125b-5p were downregulated in cases of both knee and hip OA (IVW: OR < 1, *p* < 0.05). We further explored the biological mechanisms and drug interactions of these miRNAs through bioinformatics. Quality assessment was then conducted for Mendelian Randomization (MR) studies using STROBE-MR and assumption evaluation (see Supplementary File S1). All instrumental variables (IVs) in this study exhibited F-statistics greater than 10, indicating the absence of weak instrument bias.

### 2.1. Calculating the Causal Effects of Circulating miRNAs on Knee Osteoarthritis

**Figure 1:**
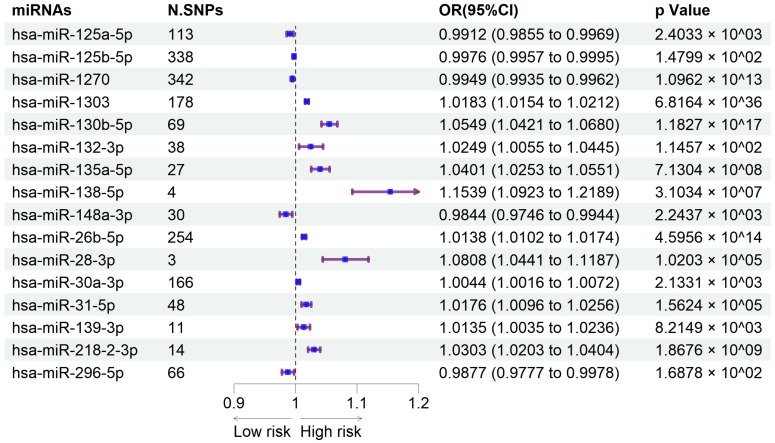
Forest maps of the causal relationships between miRNAs and the risk of knee osteoarthritis (IVW method). Purple lines represent the confidence intervals (95% CI) for each miRNA listed. Blue dots indicate the point estimates of the odds ratios (ORs) for each miRNA, with positions to the right of the vertical line suggesting an increased risk effect, and to the left suggesting a protective effect.

**Figure 2:**
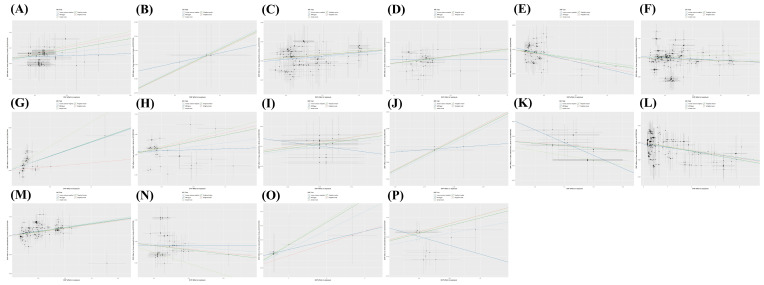
Scatter plots for Mendelian randomisation analyses of the causality of miRNAs on knee osteoarthritis. (**A**) hsa-miR-26b-5p, (**B**) hsa-miR-28-3p, (**C**) hsa-miR-30a-3p, (**D**) hsa-miR-31-5p, (**E**) hsa-miR-125a-5p, (**F**) hsa-miR-125b-5p, (**G**) hsa-miR-130b-5p, (**H**) hsa-miR-132-3p, (**I**) hsa-miR-135a-5p, (**J**) hsa-miR-138-5p, (**K**) hsa-miR-148a-3p, (**L**) hsa-miR-1270, (**M**) hsa-miR-1303, (**N**) hsa-miR-296-5p, (**O**) hsa-miR-139-3p, and (**P**) hsa-miR-218-2-3p.

Our study identified sixteen circulating miRNAs with causal associations to knee osteoarthritis, including eleven risk factors (IVW: OR > 1, *p* < 0.05) and five protective factors (IVW: OR < 1, *p* < 0.05). Results are presented in the forest plot (Figure 1) and the scatter plots (Figure 2).

In knee OA, IVW analysis suggested that 11 circulating miRNAs were associated with an increased risk of knee osteoarthritis: hsa-miR-1303 (*p* = $6.8164\times 10^{-36}$; OR = 1.0183, 95%CI = 1.0154–1.0212), hsa-miR-130b-5p (*p* = $1.1827\times 10^{-17}$; OR = 1.0549, 95%CI = 1.0421–1.0680), hsa-miR-26b-5p (*p* = $4.5956\times 10^{-14}$; OR = 1.0138, 95%CI = 1.0102–1.0174), hsa-miR-135a-5p (*p* = $7.1304\times 10^{-8}$; OR = 1.0401, 95%CI = 1.0253–1.0551), hsa-miR-138-5p (*p* = $3.1034\times 10^{-7}$; OR = 1.1539, 95%CI = 1.0923–1.2189), hsa-miR-28-3p (*p* = $1.0203\times 10^{-5}$; OR = 1.0808, 95%CI = 1.0441 –1.1187), hsa-miR-31-5p (*p* = $1.5624\times 10^{-5}$; OR = 1.0176, 95%CI = 1.0096–1.0256), hsa-miR-30a-3p (*p* = $2.1331\times 10^{-3}$; OR = 1.0044, 95%CI = 1.0016–1.0072), hsa-miR-132-3p (*p* = $1.1457\times 10^{-2}$; OR = 1.0249, 95%CI = 1.0055–1.0445), hsa-miR-139-3p (*p* = $8.2149\times 10^{-3}$; OR = 1.0135, 95%CI = 1.0035–1.0236), and hsa-miR-218-2-3p (*p* = $1.8676\times 10^{-9}$; OR = 1.0303, 95%CI = 1.0203–1.0404).

In contrast, five circulating miRNAs showed protective associations with knee osteoarthritis: hsa-miR-1270 (*p* = $1.0962\times 10^{-13}$; OR = 0.9949, 95%CI = 0.9935–0.9962), hsa-miR-148a-3p (*p* = $2.2437\times 10^{-3}$; OR = 0.9844, 95%CI = 0.9746–0.9944), hsa-miR-125a-5p (*p* = $2.4033\times 10^{-3}$; OR = 0.9912, 95%CI = 0.9855–0.9969), hsa-miR-125b-5p (*p* = $1.4799\times 10^{-2}$; OR = 0.9976, 95%CI = 0.9957–0.9995), and hsa-miR-296-5p (*p* = $1.6878\times 10^{-2}$; OR = 0.9877, 95%CI = 0.9777–0.9978).

We conducted rigorous sensitivity analyses to confirm the reliability and stability of our findings. We first performed Cochran’s Q-test to investigate the heterogeneity of the 16 miRNAs associated with knee osteoarthritis (OA). Of these, miR-125b-5p, miR-132-3p, and miR-296-5p showed significant heterogeneity in the Q-test with p-values of $2.7711\times 10^{-3}$, $1.1322\times 10^{-9}$, and $3.6471\times 10^{-2}$, respectively, and were analyzed using random effects models in MR analyses. The remaining miRNAs, showing no significant heterogeneity (*p* > 0.05 in the Q-test), were analyzed using fixed effects models. None of the 16 circulating miRNAs exhibited significant horizontal pleiotropy in the MR-Egger’s test (Table 1). Additionally, the funnel plots demonstrated a symmetrical distribution of SNPs, further substantiating the stability of our results (Supplementary File S2). The leave-one-out method was also applied to assess the influence of individual SNPs on the overall effect sizes, with no significant impacts identified (Supplementary File S3).

### 2.2. Calculating the Causal Effects of Circulating miRNAs on Hip Osteoarthritis

**Figure 3:**
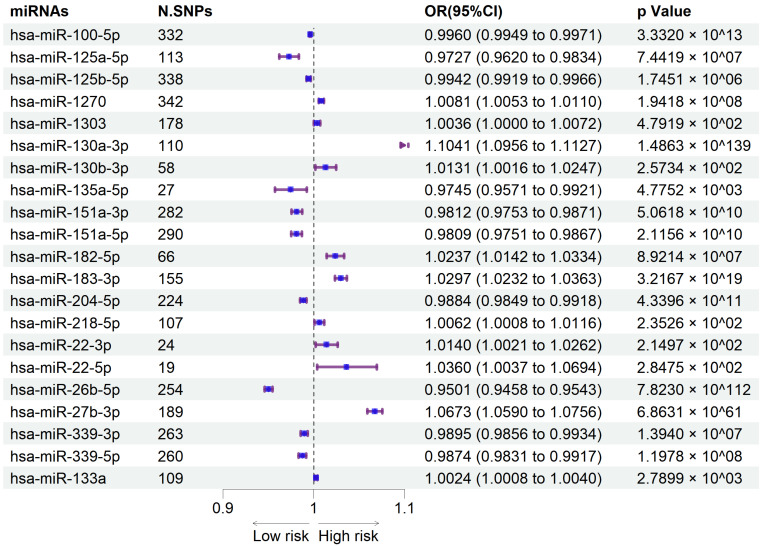
Forest maps of the causal relationships between miRNAs and the risk of hip osteoarthritis (IVW method). Purple lines represent the confidence intervals (95% CI) for each miRNA listed. Blue dots indicate the point estimates of the odds ratios (ORs) for each miRNA, with positions to the right of the vertical line suggesting an increased risk effect, and to the left suggesting a protective effect.

**Figure 4:**
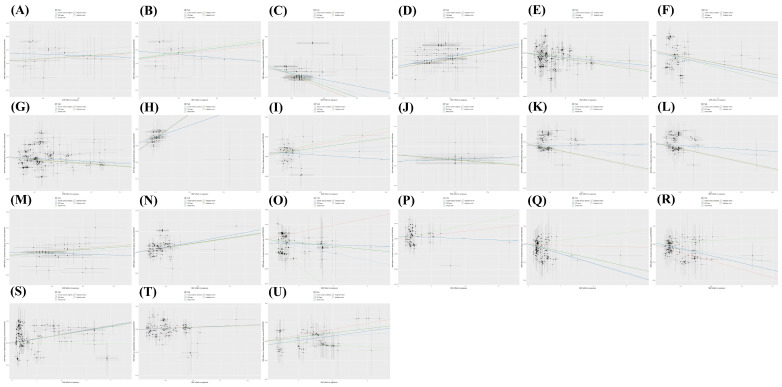
Scatter plots for Mendelian randomization analyses of the causality of miRNAs on hip osteoarthritis. (**A**) hsa-miR-22-3p, (**B**) hsa-miR-22-5p, (**C**) hsa-miR-26b-5p, (**D**) hsa-miR-27b-3p, (**E**) hsa-miR-100-5p, (**F**) hsa-miR-125a-5p, (**G**) hsa-miR-125b-5p, (**H**) hsa-miR-130a-3p, (**I**) hsa-miR-130b-3p, (**J**) hsa-miR-135a-5p, (**K**) hsa-miR-151a-3p, (**L**) hsa-miR-151a-5p, (**M**) hsa-miR-182-5p, (**N**) hsa-miR-183-3p, (**O**) hsa-miR-204-5p, (**P**) hsa-miR-218-5p, (**Q**) hsa-miR-339-3p, (**R**) hsa-miR-339-5p, (**S**) hsa-miR-1270, (**T**) hsa-miR-1303, and (**U**) hsa-miR-133a.

Our study identified 21 circulating miRNAs with causal associations to hip osteoarthritis, including 11 risk factors (IVW: OR > 1, *p* < 0.05) and 10 protective factors (IVW: OR < 1, *p* < 0.05). Results are presented in the forest plot (Figure 3) and the scatter plots (Figure 4).

In hip OA, IVW analysis suggested that 11 circulating miRNAs were associated with an increased risk of hip osteoarthritis: hsa-miR-130a-3p (*p* = $1.4863\times 10^{-139}$; OR = 1.1041, 95%CI = 1.0956–1.1127), hsa-miR-27b-3p (*p* = $6.8631\times 10^{-61}$; OR = 1.0673, 95%CI = 1.0590–1.0756), hsa-miR-183-3p (*p* = $3.2167\times 10^{-19}$; OR = 1.0297, 95%CI = 1.0232–1.0363), hsa-miR-1270 (*p* = $1.9418\times 10^{-8}$; OR = 1.0081, 95%CI = 1.0053–1.0110), hsa-miR-182-5p (*p* = $8.9214\times 10^{-7}$; OR = 1.0237, 95%CI = 1.0142–1.0334), hsa-miR-22-3p (*p* = $2.1497\times 10^{-2}$; OR = 1.0140, 95%CI = 1.0021–1.0262), hsa-miR-218-5p (*p* = $2.3526\times 10^{-2}$; OR = 1.0062, 95%CI = 1.0008–1.0116), hsa-miR-130b-3p (*p* = $2.5734\times 10^{-2}$; OR = 1.0131, 95%CI = 1.0016–1.0247), hsa-miR-22-5p (*p* = $2.8475\times 10^{-2}$; OR = 1.0360, 95%CI = 1.0037–1.0694), hsa-miR-1303 (*p* = $4.7919\times 10^{-2}$; OR = 1.0036, 95%CI = 1.0000–1.0072), and hsa-miR-133a (*p* = $2.7899\times 10^{-3}$; OR = 1.0024, 95%CI = 1.0008–1.0040).

In contrast, 10 circulating miRNAs showed protective associations with hip osteoarthritis: hsa-miR-26b-5p (*p* = $7.8230\times 10^{-112}$; OR = 0.9501, 95%CI = 0.9458–0.9543), hsa-miR-100-5p (*p* = $3.3320\times 10^{-13}$; OR = 0.9960, 95%CI = 0.9949–0.9971), hsa-miR-204-5p (*p* = $4.3396\times 10^{-11}$; OR = 0.9884, 95%CI = 0.9849–0.9918), hsa-miR-151a-5p (*p* = $2.1156\times 10^{-10}$; OR = 0.9809, 95%CI = 0.9751–0.9867), hsa-miR-151a-3p (*p* = $5.0618\times 10^{-10}$; OR = 0.9812, 95%CI = 0.9753–0.9871), hsa-miR-339-5p (*p* = $1.1978\times 10^{-8}$; OR = 0.9874, 95%CI = 0.9831–0.9917), hsa-miR-339-3p (*p* = $1.3940\times 10^{-7}$; OR = 0.9895, 95%CI = 0.9856–0.9934), hsa-miR-125a-5p (*p* = $7.4419\times 10^{-7}$; OR = 0.9727, 95%CI = 0.9620–0.9834), hsa-miR-125b-5p (*p* = $1.7451\times 10^{-6}$; OR = 0.9942, 95%CI = 0.9919–0.9966), and hsa-miR-135a-5p (*p* = $4.7752\times 10^{-3}$; OR = 0.9745, 95%CI = 0.9571–0.9921).

We conducted rigorous sensitivity analyses to confirm the reliability and stability of our findings. Cochran’s Q was utilized to determine the heterogeneity among the 21 miRNAs linked to hip osteoarthritis. Among these, miR-125a-5p, miR-125b-5p, and miR-1270 demonstrated heterogeneity in the Q-test with *p*-values of $8.8172\times 10^{-14}$, $1.3626\times 10^{-2}$, and $8.1675\times 10^{-56}$, respectively, and were analyzed using random effects models. The miRNAs without significant heterogeneity (*p* > 0.05 in the Q-test) were evaluated using fixed effects models. Moreover, none of the analyzed circulating miRNAs showed significant horizontal pleiotropy in the MR-Egger test (Table 2). The funnel plots displayed a symmetrical distribution of SNPs, thereby confirming the stability of our results (see Supplementary File S4). Furthermore, the application of the leave-one-out method confirmed that no individual SNP substantially influenced the overall conclusions, as outlined in Supplementary File S5.

### 2.3. Unveiling Biological Mechanisms and Drug Interactions of Causal Risk miRNAs

**Figure 5:**
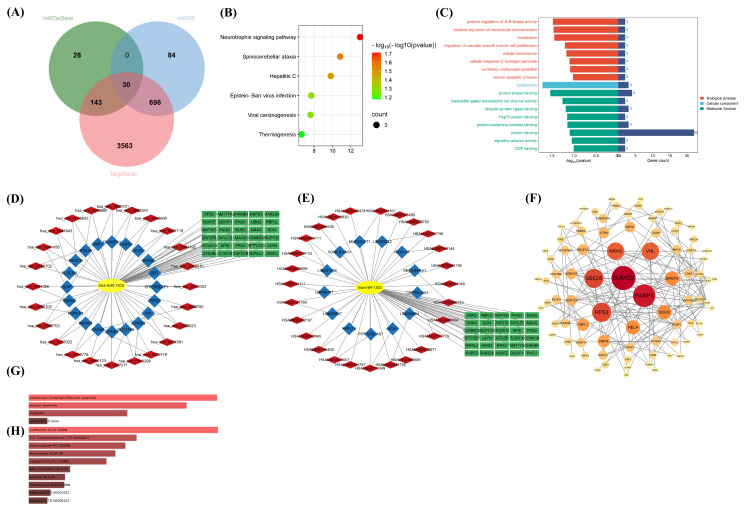
The potential biological mechanisms and drug interactions of causative risk miRNAs: (**A**) Prediction of target genes using the TargetScan, miRDB, and miRTarBase databases. (**B**) KEGG enrichment analysis highlighting the top 6 implicated pathways. (**C**) Results from GO analysis detailing biological processes, cellular components, and molecular functions. (**D**,**E**) Visualization of circRNA-miRNA-mRNA and lncRNA-miRNA-mRNA networks: (**D**) Red rhomboids represent circRNAs, blue quadrilaterals indicate the gene symbols of circRNAs, yellow ellipses are miRNAs, and green rectangles denote mRNAs. (**E**) Red rhomboids signify lncRNAs, blue squares show the gene symbols of lncRNAs, yellow ellipses represent miRNAs, and green rectangles indicate mRNAs. (**F**) Analysis of the protein–protein interaction (PPI) network and identification of key genes. (**G**,**H**) Application of the Enrichr platform to identify significant drug interactions: (**G**) Analysis of human diseases associated with these genes, utilizing the ClinVar 2019 database. (**H**) Exploration of drugs linked to these genes, employing the DSigDB. Red indicates a significance level of padj < 0.05.

Among the identified miRNAs, hsa-miR-1303 consistently emerges as a risk factor for both knee and hip osteoarthritis, with *p*-values of $6.8164\times 10^{-36}$ and $4.7919\times 10^{-2}$, respectively. To further elucidate the potential roles of hsa-miR-1303, we predicted its target genes and conducted enrichment analyses using three databases: Targetscan, miRDB, and miRTarBase. A total of 30 genes have been identified as being regulated by hsa-miR-1303 (Figure 5A, Supplementary File S6). KEGG pathway analysis highlighted that the most significantly enriched pathways included the Neurotrophin signaling pathway, Spinocerebellar ataxia, and Hepatitis C (Figure 5B). The GO enrichment analysis showed significant involvement in biological processes (BP) such as the positive regulation of JUN kinase activity and the positive regulation of microtubule polymerization localization. For Cellular Components (CC), the kinetochore was predominantly enriched. In terms of Molecular Function (MF), the most enriched terms were protein kinase binding, regulation of postsynaptic membrane potential, ubiquitin protein ligase binding, and protein binding (Figure 5C).

In addition to directly binding to target genes, miRNAs can also be bound by competitive endogenous RNAs (ceRNAs), such as lncRNAs and circRNAs. This interaction indirectly enhances the expression of target genes. Building on the ceRNA hypothesis, we utilized hsa-miR-1303 as a focal point to construct ceRNA network diagrams for lncRNA-miRNA-mRNA and circRNA-miRNA-mRNA interactions (Figure 5D,E). Within these networks, we identified several key axes, including hsa-miR-1303-hsa_circ_0041843, hsa-miR-1303-hsa_circ_0027022, hsa-miR-1303-hsa_circ_0081678, and hsa-miR-1303 interactions with LINC01338, LINC00884, and LINC01338. To further explore significant clusters involving hsa-miR-1303 within the ceRNA networks, we performed a protein–protein interaction (PPI) network analysis using the STRING database (version 11.0) and visualized the results with Cytoscape (version 3.10.3). This analysis highlighted key hub proteins such as SUMO2, PARP1, RPS3, UBE2S, and NRAS (Figure 5F). Notably, the expression of SUMO2 and PARP1 appears to be mediated by multiple lncRNAs or circRNAs via hsa-miR-1303.

Building on the target genes of hsa-miR-1303, we attempted to predict the associated disorders, drugs, and druggable genes, aiming to provide new ideas for the treatment of osteoarthritis. ClinVar analysis via Enrichr revealed a significant correlation between these target genes and disorders such as Autoimmune lymphoproliferative syndrome (ALPS), RASopathy and Noonan Syndrome [19,20,21] (Figure 5G). Autoimmune lymphoproliferative syndrome (ALPS) is a genetic disorder caused by defects in the Fas-signaling pathway, leading to impaired lymphocyte apoptosis. It is marked by lymphadenopathy, splenomegaly, hyperlymphocytosis, and the presence of circulating CD4-/CD8- double-negative T cells [21,22]. RASopathy and Noonan Syndrome are part of a family of genetic conditions caused by dysregulation in the RAS/MAPK signaling pathway, presenting with cardiovascular, lymphatic, and skeletal abnormalities [19]. Common musculoskeletal manifestations include thoracic wall anomalies, spinal deformities, flat feet, hand deformities, hip dysplasia, joint laxity or contractures, muscular hypotonia, and muscle paucity [23,24]. Further analysis with the DSigDB tool in Enrichr identified 92 drugs, including cantharidin, diindolylmethane, metronidazole, thiostrepton, and pregnenolone, which significantly impact the expression of these genes (Figure 5H). Moreover, among the predicted target genes of hsa-miR-1303, nine were identified as druggable, such as NRAS, H2AZ1, RPS3, CDKN1B, and FAM177A1, indicating that their protein products have known drug-binding sites according to the DGIdb database (Supplementary File S7). These genes are considered potential therapeutic targets for osteoarthritis. Continued validation of drugs targeting these genes could be crucial in developing effective treatments for osteoarthritis.

### 2.4. Unveiling Biological Mechanisms and Drug Interactions of Causal Protective miRNAs

**Figure 6:**
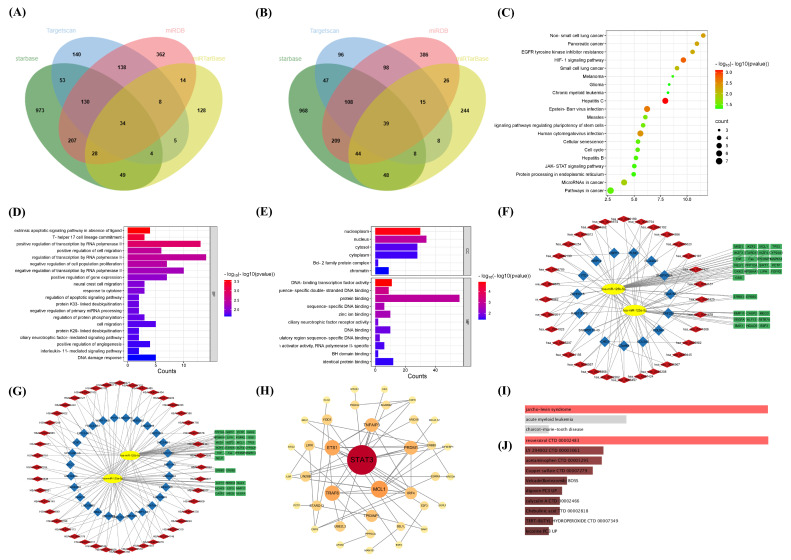
The potential biological mechanisms and drug interactions of causative protective miRNAs: (**A**,**B**) Prediction of target genes for hsa-miR-125a-5p (**A**) and hsa-miR-125b-5p (**B**) using TargetScan, StarBase, miRDB, and miRTarBase databases. (**C**) KEGG enrichment analysis identifying the top 20 pathways involved. (**D**,**E**) Results of GO analysis: (**D**) Biological processes. (**E**) Cellular components and molecular functions. (**F**,**G**) Visualizations of the circRNA-miRNA-mRNA (**F**) and lncRNA-miRNA-mRNA (**G**) networks: Red rhomboids represent circRNAs or lncRNAs. Blue squares show the gene symbols of circRNAs or lncRNAs. Yellow ellipses indicate miRNAs. Green rectangles denote mRNAs. (**H**) Analysis of the protein–protein interaction (PPI) network and identification of key genes. (**I**,**J**) Utilization of the Enrichr platform to investigate significant drug interactions: (**I**) Analysis of human diseases associated with these genes, using the ClinVar 2019 database. (**J**) Exploration of drugs related to these genes, using the DSigDB option. Red color indicates a significance level of padj < 0.05.

Among the miRNAs studied, hsa-miR-125a-5p and hsa-miR-125b-5p have been recognized as protective in both knee and hip osteoarthritis, making them prime candidates for deeper investigation into their target genes and pathways. Utilizing databases like Targetscan, Starbase, miRDB, and miRTarBase, we identified 73 target genes influenced by these two miRNAs (Figure 6A,B, and Supplementary File S8). KEGG pathway analysis indicated significant enrichment in pathways related to Hepatitis C, HIF-1 signaling pathway, Epstein–Barr virus infection, and EGFR tyrosine kinase inhibitor resistance (Figure 6C). Gene Ontology (GO) enrichment analysis further detailed the biological processes (BP) these miRNAs influence, such as the extrinsic apoptotic signaling pathway without ligand, transcription regulation by RNA polymerase II, T-helper 17 cell lineage commitment, and the regulation of cell migration and cell population proliferation (Figure 6D). Enrichment in cellular components was observed predominantly in the nucleoplasm and nucleus, while molecular function analysis highlighted enrichment in DNA-binding transcription factor activity, protein binding, and sequence-specific double-stranded DNA binding (Figure 6E).

Except for their direct interaction with target genes, miRNAs might also interact indirectly through binding by competitive endogenous RNAs (ceRNAs), such as lncRNAs and circRNAs, which can competitively enhance the expression of these targets. Based on this ceRNA hypothesis, we utilized hsa-miR-125a-5p and hsa-miR-125b-5p to construct ceRNA network diagrams for lncRNA-miRNA-mRNA and circRNA-miRNA-mRNA interactions (Figure 6F,G). Within these networks, key interactions such as miR-125 with hsa_circ_0000254, miR-125 with hsa_circ_0034156, miR-125 with hsa_circ_0081678, miR-125 with SPACA6P-AS, miR-125 with HSALNG0055678, and miR-125 with NKILA were identified, among others. We further analyzed the central clusters of these miRNAs within the ceRNA networks using the STRING database version 11.0 for protein–protein interaction (PPI) analysis, with visualizations completed in Cytoscape (version 3.10.3). This analysis highlighted central hub proteins including STAT3, MCL1, TRAF6, and ETS1 (Figure 6H). Notably, STAT3’s expression is modulated by various lncRNAs or circRNAs through interactions with both hsa-miR-125a-5p and hsa-miR-125b-5p.

Furthermore, based on these two miRNAs, we explored their potential links to diseases, therapeutic drugs, and druggable genes, aiming to suggest new approaches for osteoarthritis treatment. The ClinVar analysis on Enrichr revealed a strong link between miRNA-predicted genes and Jarcho–Levin Syndrome (JLS) (Figure 6I), a congenital condition characterized by rib and vertebral anomalies [25]. Skeletal surveys have identified multiple vertebral anomalies at different levels of the spine, including butterfly vertebrae, hemivertebrae, and fused hypoplastic vertebrae [26]. The associated small thorax size in newborns frequently leads to respiratory compromise and death in infancy [27]. Additionally, using the DSigDB option on Enrichr, we identified 47 drugs, including Resveratrol and Acetaminophen, that significantly influence the expression of these miRNA-predicted genes (Figure 6J). We recognized 22 of these genes as druggable according to the DGIdb database, such as MTF1, KLC3, STAT3, LIFR, and CBFB, making them potential targets for osteoarthritis therapy (Supplementary File S9). Advances in drug development targeting these genes may provide new therapeutic options for osteoarthritis management.

## 3. Discussion

In this research, we employed a combination of Genome-Wide Association Studies (GWAS) and cis-miR-eQTL databases along with Mendelian Randomization to investigate the causal impact of miRNAs on both knee and hip osteoarthritis. To the best of our knowledge, this is the first study to explore the causal relationships between miRNAs and osteoarthritis using extensive genetic data. Furthermore, this research is pioneering in identifying hsa-miR-1303 as a risk factor for both knee and hip OA while recognizing hsa-miR-125a-5p and hsa-miR-125b-5p as protective factors, offering critical insights and identifying novel targets for therapeutic intervention and further investigation.

In our study, hsa-miR-1303 was identified as a potential regulator of 30 genes that influence the neurotrophin signaling pathway, JUN kinase activity, and protein kinase binding. It interacts with ceRNAs such as circ_0041843 and LINC01338, impacting key proteins, including SUMO2 and PARP1. Pharmacologically, hsa-miR-1303 is associated with conditions like lymphoproliferative syndrome (ALPS), RASopathy, and Noonan Syndrome, targeting nine druggable genes and showing therapeutic potential with drugs such as cantharidin and diindolylmethane. However, the roles of hsa-miR-1303 variants in osteoarthritis have yet to be studied despite their known associations with other diseases such as tuberculosis [28], breast cancer [29], and osteosarcoma [30].

The protective miRNAs, hsa-miR-125a-5p and hsa-miR-125b-5p, were identified in this study as targeting 73 genes involved in the HIF-1 signaling pathway, Hepatitis C, and resistance to EGFR tyrosine kinase inhibitors. These miRNAs influence apoptotic signaling and transcription regulation and interact with ceRNAs such as circ_0000254 and SPACA6P-AS to affect proteins like STAT3, MCL1, and TRAF6. Drug interaction analysis revealed 22 druggable genes and 47 potential treatments, including Resveratrol and Acetaminophen, suggesting new avenues for osteoarthritis management. Furthermore, multiple studies have underscored the role of miR-125 in osteoarthritis. Xia et al. demonstrated that miR-125a-5p, abundant in exosomes from bone marrow mesenchymal stem cells, facilitates chondrocyte migration and reduces cartilage degradation by targeting E2F2, thereby alleviating osteoarthritis symptoms in vitro and in mouse models [31]. Murata et al. observed lower levels of miR-125a-5p in osteoarthritis patients compared to those with rheumatoid arthritis, suggesting its diagnostic potential [32]. Shen et al. showed that CircCDK14 sponges miR-125a-5p to modulate TGF-β signaling in osteoarthritis [33]. Meanwhile, Rasheed et al. found that miR-125b-5p inhibits inflammation by regulating the TRAF6/MAPKs/NF-κB pathway [34], and Ge et al. reported its elevated levels in severe osteoarthritis synovial cells, inversely correlated with SYVN1 expression, which it downregulates to promote apoptosis, highlighting its therapeutic potential [35].

In addition to the three miRNAs identified in our study, recent research has expanded our understanding of miRNAs in osteoarthritis (OA), highlighting their significant involvement in key OA-associated signaling pathways, including MMP13 [11], STAT3 [12], SMAD2/3 [13], NOTCH3/Notch [14], and NF-κB [15]. Li et al. found that CS-semi5, a chondroitin sulfate analog, prevents cartilage degeneration and maintains matrix homeostasis in osteoarthritis by targeting the miR-122-5p/p38/MMP13 pathway [11]. Lin et al. found that upregulating miR-653-5p in osteoarthritis reduces chondrocyte senescence and cartilage degradation via the IL-6/JAK/STAT3 pathway [12]. Qin et al. showed that blocking miR-155-5p alleviates chondrocyte senescence and osteoarthritis caused by overloading through the PIEZO1-miR-155-5p-GDF6-SMAD2/3 pathway [13]. Tang et al. reported that overexpression of circ-IQGAP1 in BMSCs accelerates osteoarthritis development by activating the NOTCH3/Notch pathway through miR-875-5p, suggesting a potential therapeutic target [14]. Chen et al. observed that circSLTM exacerbates osteoarthritis by influencing the miR-515-5p/VAPB axis and the NF-κB pathway, but its inhibition can improve chondrocyte proliferation, reduce apoptosis, and decrease inflammation in osteoarthritis models [15]. These studies highlight the critical roles of miRNAs and related networks in key pathways affecting osteoarthritis, emphasizing their potential as targets for developing more effective therapies.

In addition to their roles in OA-related pathways, microRNAs have also been linked to the staging and severity of osteoarthritis. Researchers have identified key miRNAs like hsa-miR-335-3p and hsa-miR-199a-5p essential for early knee OA detection and monitoring [36], while miRNAs such as let-7e and miRNA-454 inversely correlate with OA severity, indicating potential for predicting severe conditions [37]. Lian et al. found that miR-18a aggravates OA by enhancing NF-κB mediated inflammation and inhibiting TGF-β pathways, leading to chondrocyte degeneration [38]. In synovial fluid, Li et al. discovered a panel of seven miRNAs (23a-3p, 24-3p, 27a-3p, 27b-3p, 29c-3p, 34a-5p, and 186-5p), with miR-378a-5p prevalent in late-stage OA, useful for monitoring progression [39]. Specific miRNAs such as miR-34a-5p and miR-27b-3p are elevated in severe OA, with the former leading to cartilage damage [40] and the latter contributing to synovial fibrosis [41]. Wilson et al. highlighted miR-335-5p’s role in late-stage OA fat metabolism [42], while Lu et al. reported that miR-218-5p exacerbates cartilage degradation, which can be mitigated by targeting the PI3K/Akt/mTOR pathway [43]. Suppression of miR-10a reduces synovitis severity by affecting the TWIST1-miR-10a-MAP3K7-NF-κB pathway [44]. These findings underline the significant regulatory roles of miRNAs in OA’s stage and severity, positioning them as potential biomarkers and therapeutic targets.

In this study, we utilized circulating cis-miR-eQTLs and the OA cartilage SNPs to explore the interactions between systemic biomarkers and local changes. However, synovial miRNAs are also crucial for understanding the molecular mechanisms of osteoarthritis (OA), as they can directly reflect pathological changes in the local joint environment. Ji et al. reported that miR-182-5p is significantly downregulated in the synovial fluid of OA patients, influencing chondrocyte autophagy by targeting TNFAIP8, indicating its therapeutic potential [45]. Lu et al. observed that miR-99b-5p is upregulated in OA synovial fluid, exacerbating chondrocyte senescence by inhibiting MFG-E8 and activating NF-κB pathways [46]. Regarding synovial exosomes, Wang et al. found that miR-146a, upregulated in OA, reduces chondrocyte apoptosis and modulates macrophage polarization by inhibiting TRAF6 and impacting the Toll-like receptor 4/TRAF6/NF-κB pathway, suggesting therapeutic relevance [47]. Zhou et al. noted that exosomal miRNA-126-3p, downregulated in OA synovial fluid, promotes chondrocyte proliferation and inhibits apoptosis, reducing inflammatory cytokines like IL-1β, IL-6, and TNF-α in vitro, which helps prevent osteophyte formation and cartilage degeneration, highlighting its therapeutic potential [48]. Although circulating miRNAs are more accessible, practical, and capable of reflecting systemic impacts in a clinical setting, miRNAs in synovial fluid remain critically valuable for studying the local pathological processes of osteoarthritis.

While there is growing evidence linking miRNAs to knee and hip osteoarthritis, the causal relationships remain unclear. To our knowledge, this is the first study to explore these relationships using extensive genetic data. The key strengths of our study include the following: 1. Broad Scope and Depth: We conducted a detailed examination of a wide range of miRNAs, covering 5269 cis-miR-eQTLs—the largest set of cis-miR-eQTL data available—in relation to both knee and hip osteoarthritis. 2. Robust Methodology: By using genetic variants, we minimized confounding factors such as obesity, diabetes, cardiovascular diseases, articular injury, and physical activity. These genetic variants also represent lifelong exposure, enabling our study to capture the long-term effects of risk factors on knee and hip osteoarthritis. Additionally, rigorous sensitivity analyses confirmed the robustness of our findings, making them reliable and applicable for future laboratory and clinical research. 3. Novel Insights: We identified 16 miRNAs causally connected to knee osteoarthritis and 21 to hip osteoarthritis, and we were the first to categorize hsa-miR-1303 as a risk factor, while hsa-miR-125a-5p and hsa-miR-125b-5p were identified as protective. We also elucidated their biological mechanisms and drug interactions, providing new ideas for further research and treatment.

Despite its valuable insights, this study has certain limitations. Primarily, our analysis was confined to European populations. Therefore, extrapolating our results to other ethnic groups with distinct genetic backgrounds necessitates further research and validation. Additionally, current research on cis-miRNAs is primarily derived from blood samples, and studies on cis-miRNAs in synovial fluid are limited, hindering our acquisition of synovial cis-miR-eQTL data. Furthermore, the specific mechanisms by which miR-1303 influences osteoarthritis remain elusive, warranting further investigation. Lastly, due to incomplete information on gender, ethnicity, and socioeconomic status, we were unable to perform related stratification analyses. Therefore, the depth of our research is insufficient and necessitates further comprehensive studies to overcome these limitations.

## 4. Materials and Methods

### 4.1. Data Acquisition

In this study, we utilized circulating cis-miR-eQTLs and OA cartilage SNPs to explore the interactions between systemic biomarkers and local changes. We employed the largest dataset of circulating cis-miRNA expression quantitative loci (cis-miR-eQTLs) to date as our exposure. This study comprehensively examined 5329 blood samples, through which it identified 5269 cis-miR-eQTLs associated with 76 mature microRNAs [49]. Gender, age, BMI, blood pressure, cholesterol, and glucose were corrected during the analysis. Data on different types of osteoarthritis were obtained from the IEU OpenGWAS project (https://gwas.mrcieu.ac.uk/ (accessed on 13 October 2024)). The knee osteoarthritis dataset included 403,124 European participants, comprising 24,955 cases and 378,169 controls, and identified 29,999,696 independent SNPs. The hip osteoarthritis dataset included 393,873 participants, comprising 15,704 cases and 378,169 controls, with a total of 29,771,219 SNPs. Both datasets were derived from the research conducted by Tachmazidou et al. in the UK Biobank [50]. Detailed information on data sources and characteristics can be found in Table 3.

### 4.2. Selection of IVs

Genetic instrumental variables for cis-miR-eQTLs were meticulously selected based on a genome-wide significance threshold of $p<5\times 10^{-8}$. We set the parameter r threshold at 0.001 and the SNP distance at 10,000 kb to minimize the effects of linkage disequilibrium (LD) among the cis-miR-eQTLs. We systematically removed all SNPs associated with confounding factors for OA, including BMI, hip circumference, waist circumference, body weight, body height, LDL, VLDL, IDL, cholesterol, triglycerides, apolipoprotein B, blood pressure, diabetes, cardiovascular diseases, articular injury, insomnia, depression, and strenuous exercise, using the association feature on the GWAS Catalog (https://www.ebi.ac.uk/gwas/home (accessed on 25 October 2024)) and the FastTraitR R package. To address the potential influence of weak instrumental variables, a heterogeneity test was used to exclude SNPs showing significant variance, retaining only those with an F-statistic greater than 10 for use as instrumental variables [51].

### 4.3. Statistical Analysis

The MR analyses were performed using R version 4.2.1 and the TwoSampleMR package (https://github.com/MRCIEU/TwoSampleMR.git (accessed on 7 October 2024)). A variety of estimation methods were used, including inverse variance weighted (IVW) [52], weighted median [53], simple mode, weighted mode, and MR-Egger regression [54]. IVW served as the primary analytical method, while other methods were used as supplements [55]. We performed multiple sensitivity analyses to address potential issues of heterogeneity and pleiotropy [56]. Cochran’s Q-test was used to assess heterogeneity, and MR-Egger’s test was employed to evaluate horizontal pleiotropy [57]. The leave-one-out analysis was performed to determine if a single SNP significantly influenced the causal effect. The MR-PRESSO test was applied to identify and correct for pleiotropy and to eliminate outlier SNPs [56]. These statistical methods enhance the study’s reliability and precision. All results are presented as odds ratios (ORs) with 95% confidence intervals (CIs), deemed statistically significant at *p* < 0.05.

### 4.4. Mendelian Randomization

MR analysis employs genetic variation as a proxy for risk factors and relies on validated instrumental variables (IVs) to meet three key hypotheses in causal inference: 1. The Relevance hypothesis: IVs are directly associated with exposure factors; 2. The Independent hypothesis: IVs are independent of any potential confounders that impact exposure and outcome; 3. The Exclusionary hypothesis: IVs influence outcome factors only through exposure factors [58]. All data employed were obtained from publicly accessible sources, were previously approved by relevant ethics committees, and involved no personal or identifiable information. No new human data were collected, and no ethical review was required.

### 4.5. Quality Assessment

In our study, we evaluated the quality of Mendelian Randomization (MR) research using the guidelines from the STROBE-MR Statement (Strengthening the Reporting of Observational Studies in Epidemiology Using Mendelian Randomization) [59]. The STROBE-MR checklist, detailed on their website (https://www.strobe-mr.org/ (accessed on 23 November 2024)), includes 20 specific items organized into several categories: Title and Abstract (item 1), Introduction (items 2–3), Methods (items 4–9), Results (items 10–13), Discussion (items 14–17), and Other Information (items 18–20). The checklist promotes clear reporting of the assumptions made in the models and the sensitivity analyses, which were key aspects assessed in our research.

### 4.6. miRNA Target Prediction

To further explore the potential functions of the identified miRNAs, we used Targetscan [60] (https://www.targetscan.org/ (accessed on 2 November 2024)), Starbase [61] (https://rnasysu.com/encori/ (accessed on 2 November 2024)), miRDB [62] (https://mirdb.org/ (accessed on 2 November 2024)), and miRTarBase [63] (https://mirtarbase.cuhk.edu.cn/ (accessed on 2 November 2024)) to predict the target genes of these miRNAs. We considered only experimentally validated target genes. These tools utilize different algorithms and databases to provide a comprehensive analysis of miRNA-target interactions. To optimize the sensitivity of our predictions and minimize potential false negatives, we integrated the results from all four databases, selecting the union of predicted targets as indicated by the minimum overlap in the corresponding Venn diagrams. This integrative approach helps in identifying a more reliable set of potential targets by leveraging the strengths and minimizing the weaknesses of each prediction tool.

### 4.7. GO and KEGG Analysis

For the analysis of gene ontology (GO) and pathway enrichment, we categorized the relevant miRNA target genes into biological processes (BP), molecular functions (MF), and cellular components (CC) using the Database for Annotation, Visualization, and Integrated Discovery (DAVID) and the Kyoto Encyclopedia of Genes and Genomes (KEGG) for pathway enrichment analysis. The analysis was executed using the clusterProfiler R package (version 4.14.4) and an online platform [64], adjusting for gene length and identifying significant pathways and GO terms that had the most substantial gene representation, with a significance threshold set at a *p*-value of 0.05.

### 4.8. ceRNA Regulatory Networks

Using miRNAs as the central elements, circRNAs and lncRNAs as the decoys, and mRNAs as the targets, we established the ceRNA network diagrams for lncRNA-miRNA-mRNA and circRNA-miRNA-mRNA [65,66]. In these networks, circRNAs and lncRNAs act as competitive endogenous RNAs (ceRNAs) that sequester miRNAs from their mRNA targets, thus modulating gene expression. For the construction of the ceRNA network, LncBook [67] (https://ngdc.cncb.ac.cn/lncbook/home (accessed on 11 November 2024)) and CircBank [68] (http://www.circbank.cn/ (accessed on 11 November 2024)) were utilized to forecast target lncRNAs and circRNAs. Cytoscape [69] software (version 3.10.3) was used to build and display the ceRNA networks.

### 4.9. PPI Regulatory Networks

The protein–protein interaction (PPI) networks were constructed using the STRING database [70] (version 11.0), which is renowned for its extensive interactions and functional associations between proteins. These interactions were visualized in Cytoscape [69] (version 3.10.3) with a stringent cut-off value of >0.4 for the combined score to ensure the relevance and strength of the displayed interactions. This approach allowed us to delineate the complex interplay of proteins influenced by miRNA activity, providing insights into the molecular underpinnings of osteoarthritis and identifying potential molecular targets for therapeutic intervention.

### 4.10. Druggable Analysis

To thoroughly examine the molecular mechanisms affecting osteoarthritis and pinpoint promising drug candidates, we uploaded all relevant targeted genes discovered in our research to the Enrichr online platform [71] (https://maayanlab.cloud/Enrichr/ (accessed on 19 November 2024)). The ClinVar option was employed to determine the association of these genes with human diseases, and the DSigDB option was used to identify drugs that significantly alter their expression. A statistical threshold of *p*_adj < 0.05 was applied. The druggable gene set used in this study was sourced from the DGIdb database [72] (https://dgidb.org/ (accessed on 19 November 2024)). By integrating the druggable genes, we aimed to reveal the druggability of the causal genes identified in our research.

## 5. Conclusions

In conclusion, this study provides a comprehensive overview of the causal relationships between miRNAs and both knee and hip osteoarthritis. The research identifies 16 miRNAs causally connected to knee osteoarthritis and 21 to hip osteoarthritis. We were the first to categorize hsa-miR-1303 as a risk factor, while hsa-miR-125a-5p and hsa-miR-125b-5p were identified as protective. Additionally, it recognizes their biological mechanisms and drug interactions. These findings offer novel insights into the mechanisms of osteoarthritis and suggest new targets for treatment and risk assessment of knee and hip OA.

## Figures and Tables

**Table 1:** Results of sensitivity analysis of miRNAs on the risk of knee osteoarthritis.

miRNAs	Inverse Variance Weighted			MR Egger		
	Q	Q_df	Q_pval	Intercept	se	Pval
miR-26b-5p	34.7923	253	1.0000	0.0034	0.0039	0.3793
miR-28-3p	0.0491	2	0.9757	0.0150	0.0691	0.8636
miR-30a-3p	82.4031	165	1.0000	−0.0019	0.0029	0.5026
miR-31-5p	15.5080	47	1.0000	0.0073	0.0081	0.3733
miR-125a-5p	42.7909	112	1.0000	0.0025	0.0026	0.3396
miR-125b-5p	413.4771	337	0.0028	0.0026	0.0014	0.0593
miR-130b-5p	67.5879	68	0.4913	0.0002	0.0045	0.9582
miR-132-3p	113.3294	37	0.0000	0.0072	0.0096	0.4560
miR-135a-5p	5.0379	26	1.0000	0.0205	0.0496	0.6828
miR-138-5p	0.1208	3	0.9892	0.0204	0.0608	0.7693
miR-148a-3p	9.1334	29	0.9998	0.0303	0.0173	0.0910
miR-1270	279.7342	341	0.9934	−0.0014	0.0007	0.0528
miR-1303	136.3953	177	0.9896	0.0014	0.0018	0.4513
miR-139-3p	0.6988	10	1.0000	0.0065	0.0081	0.4422
miR-218-2-3p	13.5615	13	0.4054	0.0473	0.0255	0.0882

**Table 2:** Results of sensitivity analysis of miRNAs on the risk of hip osteoarthritis.

miRNAs	Inverse Variance Weighted			MR Egger		
	Q	Q_df	Q_pval	Intercept	se	Pval
miR-22-3p	8.6079	23	0.9971	0.0087	0.0093	0.3616
miR-22-5p	7.8917	18	0.9802	0.0113	0.0133	0.4091
miR-26b-5p	56.2075	253	1.0000	−0.0064	0.0049	0.1936
miR-27b-3p	29.8430	188	1.0000	0.0024	0.0086	0.7802
miR-100-5p	301.3135	331	0.8778	−0.0012	0.0015	0.4153
miR-125a-5p	259.9559	112	0.0000	−0.0023	0.0049	0.6410
miR-125b-5p	396.8821	337	0.0136	0.0016	0.0017	0.3541
miR-130a-3p	17.9411	109	1.0000	0.0161	0.0116	0.1680
miR-130b-3p	38.5184	57	0.9713	0.0068	0.0058	0.2408
miR-135a-5p	0.8487	26	1.0000	−0.0097	0.0622	0.8770
miR-151a-3p	107.6494	281	1.0000	−0.0051	0.0033	0.1187
miR-151a-5p	108.3825	289	1.0000	−0.0022	0.0038	0.5636
miR-182-5p	33.1607	65	0.9996	0.0110	0.0092	0.2337
miR-183-3p	125.9891	154	0.9521	−0.0019	0.0040	0.6358
miR-204-5p	254.8952	223	0.0701	−0.0062	0.0034	0.0663
miR-218-5p	88.0118	106	0.8974	0.0043	0.0030	0.1530
miR-339-3p	79.7054	262	1.0000	0.0002	0.0016	0.9202
miR-339-5p	72.3811	259	1.0000	0.0028	0.0019	0.1413
miR-1270	928.0103	341	0.0000	0.0006	0.0015	0.6807
miR-1303	91.8105	177	1.0000	−0.0010	0.0023	0.6744
miR-133a	54.7812	108	1.0000	−0.0042	0.0028	0.1328

**Table 3:** Detailed information on the analyzed data.

Exposure/Outcome	Population	Year	Ncases	Ncontrols	Number of SNPs	Data Source
knee osteoarthritis	European	2019	24,955	378,169	29,999,696	https://gwas.mrcieu.ac.uk/datasets/ebi-a-GCST007090/ (accessed on 13 October 2024)
hip osteoarthritis	European	2019	15,704	378,169	29,771,219	https://gwas.mrcieu.ac.uk/datasets/ebi-a-GCST007091/ (accessed on 13 October 2024)
cis-miR-eQTLs	European	2015	5239	\	5269	https://doi.org/10.1038/ncomms7601 (accessed on 18 October 2024)

## Data Availability

All summary statistics used in this study are publicly available for download in the IEU OpenGWAS project (https://gwas.mrcieu.ac.uk/ (accessed on 13 October 2024)) as shown in Table 3. All datasets generated for this study are included in the manuscript and its Supplementary Files.

## Supplementary Materials

The following supporting information can be downloaded at: https://www.mdpi.com/article/10.3390/ijms26010283/s1. References [59,73] are cited in the supplementary materials.
