# Supplementary material for: Investigating the Impact of Circulating MicroRNAs on Knee and Hip Osteoarthritis: Causal Links, Biological Mechanisms, and Drug Interactions
Source: Int J Mol Sci. 2024 Dec 31;26(1):283. doi: 10.3390/ijms26010283 (PMC11720664; doi:10.3390/ijms26010283)
Supplement: Supplementary file 1 [file ijms-26-00283-s001.zip › Supplement File S1. STROBE-MR-checklist-fillable.pdf]

STROBE-MR checklist of recommended items to address in reports of Mendelian randomization studies[1 2]

Item No.	Section		Checklist item	Page No.	Relevant text from manuscript
1	**TITLE and ABSTRACT**		Indicate Mendelian randomization (MR) as the study's design in the title and/or the abstract if that is a main purpose of the study	1	MR is utilized merely as a method for selecting pathogenic miRNAs associated with osteoarthritis. The primary aim of our study is to investigate the biological mechanisms and drug interactions of OA-related miRNAs.
	**INTRODUCTION**				
2	**Background**		Explain the scientific background and rationale for the reported study. What is the exposure? Is a potential causal relationship between exposure and outcome plausible? Justify why MR is a helpful method to address the study question	2	However, due to inherent limitations in traditional research designs, these studies could not completely rule out the risk of reverse causality or the influence of confounding factors, such as obesity, diabetes, cardiovascular diseases, joint injuries, and physical activity, which could lead to biased results and conclusions [8]. Additionally, these findings typically indicate associations rather than causations, which compromises the reliability of miRNAs as OA biomarkers.
3	**Objectives**		State specific objectives clearly, including pre-specified causal hypotheses (if any). State that MR is a method that, under specific assumptions, intends to estimate causal effects	2	Mendelian Randomization (MR) is a robust method that uses genetic variants as instrumental variables (IVs) to estimate the causal effects of exposures on outcomes. This method helps reduce confounding biases because genetic variants are assigned at conception, and it also prevents reverse causation, since these variants exist before the disease develops [9, 10].
	**METHODS**				
4	**Study design and data sources**		Present key elements of the study design early in the article. Consider including a table listing sources of data for all phases of the study. For each data source contributing to the analysis, describe the following:		
		a)	Setting: Describe the study design and the underlying population, if possible. Describe the setting, locations, and relevant dates, including periods of recruitment, exposure, follow-up, and data collection, when available.	13	In this study, we used the largest cis-miRNA expression quantitative loci (cis-miR-eQTLs) data to date as our exposure. This study comprehensively examined 5,329 blood samples, through which it identified 5,269 cis-miR-eQTLs associated with 76 mature microRNAs [11]. Gender, age, BMI, blood pressure, cholesterol, and glucose were corrected during the analysis.

					Data on different types of osteoarthritis were obtained from the IEU OpenGWAS project (https://gwas.mrcieu.ac.uk/).
		b)	Participants: Give the eligibility criteria, and the sources and methods of selection of participants. Report the sample size, and whether any power or sample size calculations were carried out prior to the main analysis	14	Table3
		c)	Describe measurement, quality control and selection of genetic variants	14	Genetic instrumental variables for cis-miR-eQTLs were meticulously selected based on a genome-wide significance threshold of p < 5×10^–8. We set the parameter r threshold at 0.001 and the SNP distance at 10,000 kb to minimize the effects of linkage disequilibrium (LD) among the cis-miR-eQTLs.
		d)	For each exposure, outcome, and other relevant variables, describe methods of assessment and diagnostic criteria for diseases	14	The knee osteoarthritis dataset included 403,124 European participants, comprising 24,955 cases and 378,169 controls, and identified 29,999,696 independent SNPs. The hip osteoarthritis dataset included 393,873 participants, comprising 15,704 cases and 378,169 controls, with a total of 29,771,219 SNPs. Both datasets were derived from the research conducted by Tachmazidou et al. in the UK Biobank [12].
		e)	Provide details of ethics committee approval and participant informed consent, if relevant	\	All data employed was obtained from publicly accessible sources, was previously approved by relevant ethics committees, and involved no personal or identifiable information. No new human data were collected, and no ethical review was required.
5	**Assumptions**		Explicitly state the three core IV assumptions for the main analysis (relevance, independence and exclusion restriction) as well assumptions for any additional or sensitivity analysis	15	MR analysis employs genetic variation as a proxy for risk factors and relies on validated instrumental variables (IVs) to meet three key hypotheses in causal inference: 1. The Relevance hypothesis: IVs are directly associated with exposure factors; 2. The Independent hypothesis: IVs are independent of any potential confounders that impact exposure and outcome; 3. The Exclusionary hypothesis: IVs influence outcome factors only through exposure factors [21].
6	**Statistical methods: main analysis**		Describe statistical methods and statistics used		

		a) Describe how quantitative variables were handled in the analyses (i.e., scale, units, model)	14	All results are presented as odds ratios (ORs) with 95% confidence intervals (CIs), deemed statistically significant at $p < 0.05$.
		b) Describe how genetic variants were handled in the analyses and, if applicable, how their weights were selected	14	Genetic instrumental variables for cis-miR-eQTLs were meticulously selected based on a genome-wide significance threshold of p < 5×10^–8. We set the parameter r threshold at 0.001 and the SNP distance at 10,000 kb to minimize the effects of linkage disequilibrium (LD) among the cis-miR-eQTLs.
		c) Describe the MR estimator (e.g. two-stage least squares, Wald ratio) and related statistics. Detail the included covariates and, in case of two-sample MR, whether the same covariate set was used for adjustment in the two samples	14	A variety of estimation methods were used, including inverse variance weighted (IVW) [15], weighted median [16], simple mode, weighted mode, and MR-Egger regression [17]. IVW served as the primary analytical method, while other methods were used as supplements [18].
		d) Explain how missing data were addressed	\	The data used in our analysis were complete with no instances of missing entries. Therefore, no specific measures or techniques were required to address missing data in this study.
		e) If applicable, indicate how multiple testing was addressed	14	A variety of estimation methods were used, including inverse variance weighted (IVW) [15], weighted median [16], simple mode, weighted mode, and MR-Egger regression [17]. IVW served as the primary analytical method, while other methods were used as supplements [18].
7	**Assessment of assumptions**	Describe any methods or prior knowledge used to assess the assumptions or justify their validity	14	Cochran's Q-test was used to assess heterogeneity, and MR-Egger's test was employed to evaluate horizontal pleiotropy [20].
8	**Sensitivity analyses and additional analyses**	Describe any sensitivity analyses or additional analyses performed (e.g. comparison of effect estimates from different approaches, independent replication, bias analytic techniques, validation of instruments, simulations)	14	The leave-one-out analysis was performed to determine if a single SNP significantly influenced the causal effect. The MR-PRESSO test was applied to identify and correct for pleiotropy and to eliminate outlier SNPs [19].
9	**Software and pre-registration**			
		a) Name statistical software and package(s), including version and settings used	14	The MR analyses were performed using R version 4.2.1 and the TwoSampleMR package.

		b)	State whether the study protocol and details were pre-registered (as well as when and where)	\	The study protocol and details were not pre-registered.
	**RESULTS**				
10	**Descriptive data**				
		a)	Report the numbers of individuals at each stage of included studies and reasons for exclusion. Consider use of a flow diagram	\	Mendelian randomization (MR) is not the main focus of our study but merely a screening method; therefore, a flow diagram or reporting the numbers of individuals at each stage and reasons for exclusion is not required.
		b)	Report summary statistics for phenotypic exposure(s), outcome(s), and other relevant variables (e.g. means, SDs, proportions)	14	Table3
		c)	If the data sources include meta-analyses of previous studies, provide the assessments of heterogeneity across these studies	\	The data do not include meta-analyses of previous studies.
		d)	For two-sample MR:<br>i. Provide justification of the similarity of the genetic variant-exposure associations between the exposure and outcome samples<br>ii. Provide information on the number of individuals who overlap between the exposure and outcome studies	2	The exposure and outcome data are derived from two distinct databases, IEU and UK Biobank, resulting in lower similarity and overlap of the genetic variant-exposure associations between the samples.
11	**Main results**				
		a)	Report the associations between genetic variant and exposure, and between genetic variant and outcome, preferably on an interpretable scale	2	All instrumental variables (IVs) in this study exhibited F-statistics greater than 10, indicating the absence of weak instrument bias.
		b)	Report MR estimates of the relationship between exposure and outcome, and the measures of uncertainty from the MR analysis, on an interpretable scale, such as odds ratio or relative risk per SD difference	3-7	In knee OA, IVW analysis suggested that 11 circulating miRNAs were associated with an increased risk of knee osteoarthritis: hsa-miR-1303 (p = 6.8164E-36; OR = 1.0183, 95%CI = 1.0154 - 1.0212), hsa-miR-130b-5p (p = 1.1827E-17; OR = 1.0549, 95%CI = 1.0421 - 1.0680), hsa-miR-26b-5p (p = 4.5956E-14; OR = 1.0138, 95%CI = 1.0102 - 1.0174), hsa-miR-135a-5p (p = 7.1304E-08; OR = 1.0401, 95%CI = 1.0253 - 1.0551), hsa-miR-138-5p (p = 3.1034E-07; OR = 1.1539, 95%CI = 1.0923 - 1.2189), hsa-miR-28-3p (p = 1.0203E-05; OR = 1.0808, 95%CI = 1.0441 - 1.1187), hsa-miR-31-5p (p = 1.5624E-05; OR = 1.0176, 95%CI = 1.0096 - 1.0256), hsa-miR-30a-3p (p = 2.1331E-03; OR = 1.0044, 95%CI = 1.0016 - 1.0072), hsa-miR-132-3p (p = 1.1457E-

					02; OR = 1.0249, 95%CI = 1.0055 - 1.0445), hsa-miR-139-3p (p = 8.2149E-03; OR = 1.0135, 95%CI = 1.0035 - 1.0236), and hsa-miR-218-2-3p (p = 1.8676E-09; OR = 1.0303, 95%CI = 1.0203 - 1.0404).
		c)	If relevant, consider translating estimates of relative risk into absolute risk for a meaningful time period	\	The type of disease studied does not require conversion of relative risk estimates into absolute risk.
		d)	Consider plots to visualize results (e.g. forest plot, scatterplot of associations between genetic variants and outcome versus between genetic variants and exposure)	3,5,6	Results are presented in the forest plot (Figure 1,3) and the scatter plots (Figure 2,4).
12	**Assessment of assumptions**				
		a)	Report the assessment of the validity of the assumptions	2	All instrumental variables (IVs) in this study exhibited F-statistics greater than 10, indicating the absence of weak instrument bias. Sensitivity analyses have confirmed the robustness of our results.
		b)	Report any additional statistics (e.g., assessments of heterogeneity across genetic variants, such as I^2, Q statistic or E-value)	3,5	Of these, 3 miRNAs showed significant heterogeneity (P < 0.05 in the Q-test) and were analyzed using random effects models in Mendelian randomization (MR), while the remaining miRNAs without significant heterogeneity (P > 0.05 in the Q-test) were analyzed using fixed effects models.
13	**Sensitivity analyses and additional analyses**				
		a)	Report any sensitivity analyses to assess the robustness of the main results to violations of the assumptions	4,6	Table2,3
		b)	Report results from other sensitivity analyses or additional analyses	4,7	None of the 16 circulating miRNAs exhibited significant horizontal pleiotropy in the MR-Egger's test (Table 2). Additionally, the funnel plots demonstrated a symmetrical distribution of SNPs, further substantiating the stability of our results (Supplementary File 1). The leave-one-out method was also applied to assess the influence of individual SNPs on the overall effect sizes, with no significant impacts identified (Supplementary File

					2).
		c)	Report any assessment of direction of causal relationship (e.g., bidirectional MR)	\	No bidirectional Mendelian randomization was conducted in this study.
		d)	When relevant, report and compare with estimates from non-MR analyses	11-13	However, the roles of hsa-miR-1303 variants in osteoarthritis have yet to be studied, despite their known associations with other diseases such as tuberculosis[44], breast cancer[45], and osteosarcoma[46].<br>Furthermore, multiple studies have underscored the role of miR-125 in osteoarthritis......
		e)	Consider additional plots to visualize results (e.g., leave-one-out analyses)	Supplement Files	Funnel polts (Supplement File 2,4)<br>Leave-one-out forest maps (Supplement File 3,5)
	**DISCUSSION**				
14	**Key results**		Summarize key results with reference to study objectives	11	We identified 16 miRNAs associated with knee osteoarthritis and 21 linked to hip osteoarthritis. Notably, hsa-miR-1303 was identified as a risk factor, whereas hsa-miR-125a-5p and hsa-miR-125b-5p were found to be protective across all datasets, including knee OA, hip OA, and validation OA data.
15	**Limitations**		Discuss limitations of the study, taking into account the validity of the IV assumptions, other sources of potential bias, and imprecision. Discuss both direction and magnitude of any potential bias and any efforts to address them	13	Primarily, our analysis was confined to European populations to ensure genetic homogeneity. Consequently, extrapolating our results to other ethnic groups with distinct genetic backgrounds requires additional research and validation. Secondly, the specific mechanisms of miR-1303 in osteoarthritis remain unclear. Further research into these mechanisms is required.
16	**Interpretation**				
		a)	Meaning: Give a cautious overall interpretation of results in the context of their limitations and in comparison with other studies	3,5	Our study identified 16 circulating miRNAs with causal associations to knee osteoarthritis, including 11 risk factors (IVW: OR>1, P<0.05) and 5 protective factors (IVW: OR<1, P<0.05). Results are presented in the forest plot (Figure 1) and the scatter plots (Figure 2).
		b)	Mechanism: Discuss underlying biological mechanisms that could drive a potential causal relationship between the investigated exposure and the outcome, and whether the gene-environment equivalence assumption is reasonable. Use causal language	7-11	2.3 Unveiling Biological Mechanisms and Drug Interactions of Causal Risk miRNAs

		carefully, clarifying that IV estimates may provide causal effects only under certain assumptions		2.4 Unveiling Biological Mechanisms and Drug Interactions of Causal Protective miRNAs
		c) Clinical relevance: Discuss whether the results have clinical or public policy relevance, and to what extent they inform effect sizes of possible interventions	12	Furthermore, multiple studies have underscored the role of miR-125 in osteoarthritis. Xia et al. demonstrated that miR-125a-5p, abundant in exosomes from bone marrow mesenchymal stem cells, facilitates chondrocyte migration and reduces cartilage degradation by targeting E2F2, thereby alleviating osteoarthritis symptoms in vitro and in mouse models[47]. Murata et al. observed lower levels of miR-125a-5p in osteoarthritis patients compared to those with rheumatoid arthritis, suggesting its diagnostic potential[48]. Shen et al. showed that CircCDK14 sponges miR-125a-5p to modulate TGF-βsignaling in osteoarthritis[49]. Meanwhile, Rasheed et al. found that miR-125b-5p inhibits inflammation by regulating the TRAF6/MAPKs/NF-κB pathway[50], and Ge et al. reported its elevated levels in severe osteoarthritis synovial cells, inversely correlated with SYVN1 expression, which it downregulates to promote apoptosis, highlighting its therapeutic potential[51].
17	**Generalizability**	Discuss the generalizability of the study results (a) to other populations, (b) across other exposure periods/timings, and (c) across other levels of exposure	13	Primarily, our analysis was confined to European populations to ensure genetic homogeneity. Consequently, extrapolating our results to other ethnic groups with distinct genetic backgrounds requires additional research and validation.
	**OTHER INFORMATION**			
18	**Funding**	Describe sources of funding and the role of funders in the present study and, if applicable, sources of funding for the databases and original study or studies on which the present study is based	17	This work was supported by the National Natural Science Foundation of China (No.81972046, and No.82372410), and the National High Level Hospital Clinical Research Funding (Grant No. 2022-PUMCH-B-001).
19	**Data and data sharing**	Provide the data used to perform all analyses or report where and how the data can be accessed, and reference these sources in the article. Provide the statistical code needed to reproduce the results in the article, or report whether the code is publicly accessible and if so, where	17	All summary statistics used in this study are publicly available for download in the IEU OpenGWAS project (\url{https://gwas.mrcieu.ac.uk/}) as shown in Table 3. All datasets generated for this study are included in the manuscript and its Supplementary Files.

20	**Conflicts of Interest**	All authors should declare all potential conflicts of interest	17	The authors declare no competing interests.

This checklist is copyrighted by the Equator Network under the Creative Commons Attribution 3.0 Unported (CC BY 3.0) license.

1. Skrivankova VW, Richmond RC, Woolf BAR, Yarmolinsky J, Davies NM, Swanson SA, et al. Strengthening the Reporting of Observational Studies in Epidemiology using Mendelian Randomization (STROBE-MR) Statement. JAMA. 2021;326(16):1614-1621.

2. Skrivankova VW, Richmond RC, Woolf BAR, Davies NM, Swanson SA, VanderWeele TJ, et al. Strengthening the Reporting of Observational Studies in Epidemiology using Mendelian Randomisation (STROBE-MR): Explanation and Elaboration. BMJ. 2021;375:n2233.
