# Supplementary figures and images for: Investigating the Impact of Circulating MicroRNAs on Knee and Hip Osteoarthritis: Causal Links, Biological Mechanisms, and Drug Interactions
Source: Int J Mol Sci. 2024 Dec 31;26(1):283. doi: 10.3390/ijms26010283 (PMC11720664; doi:10.3390/ijms26010283)

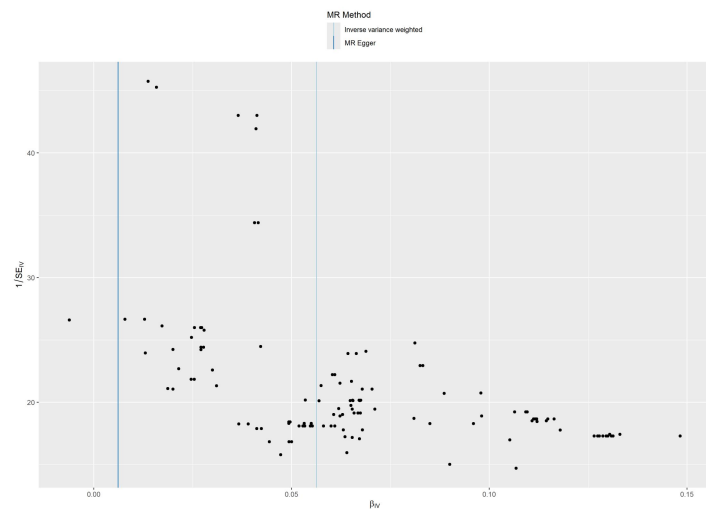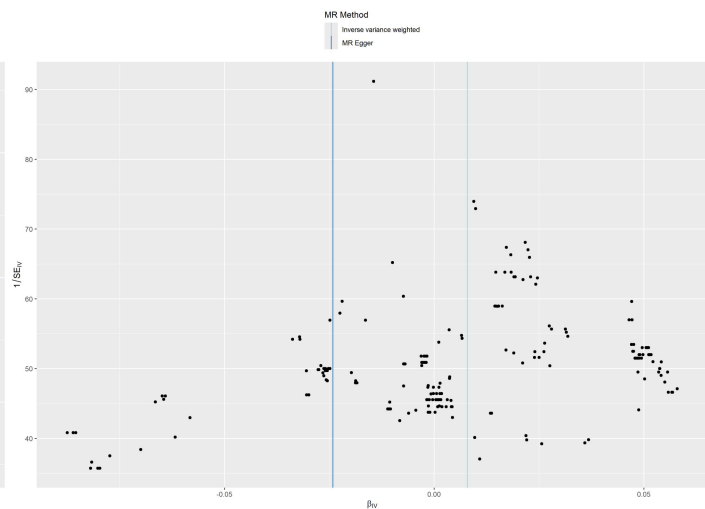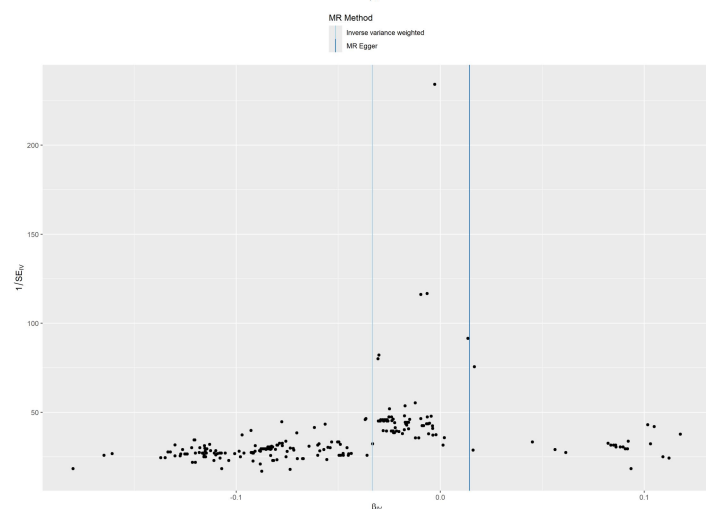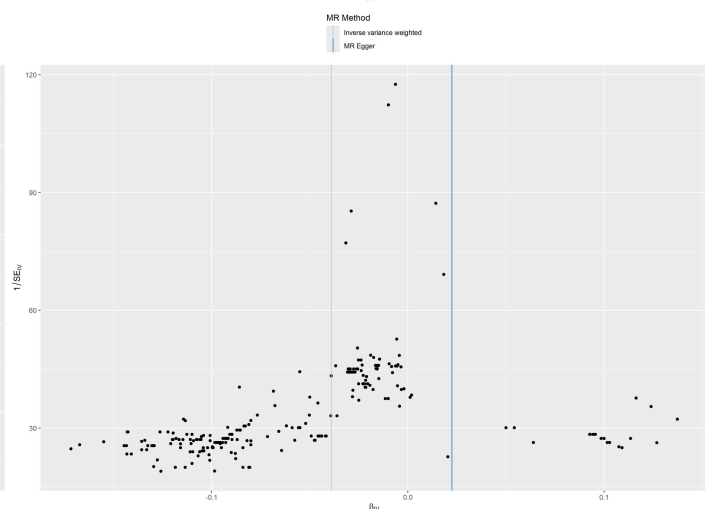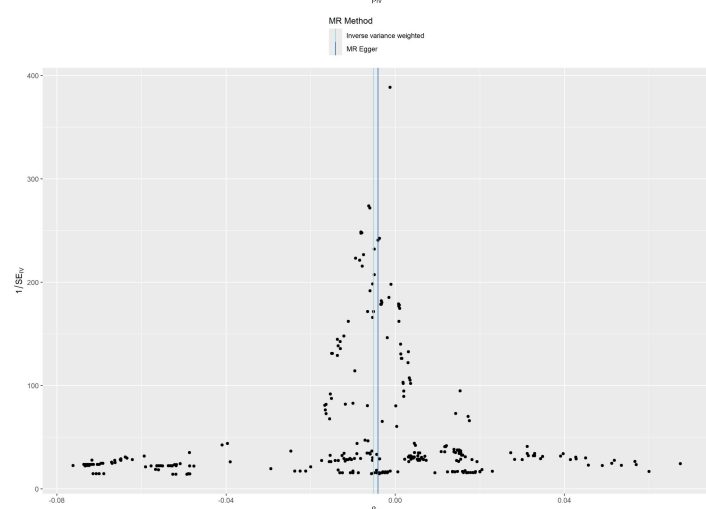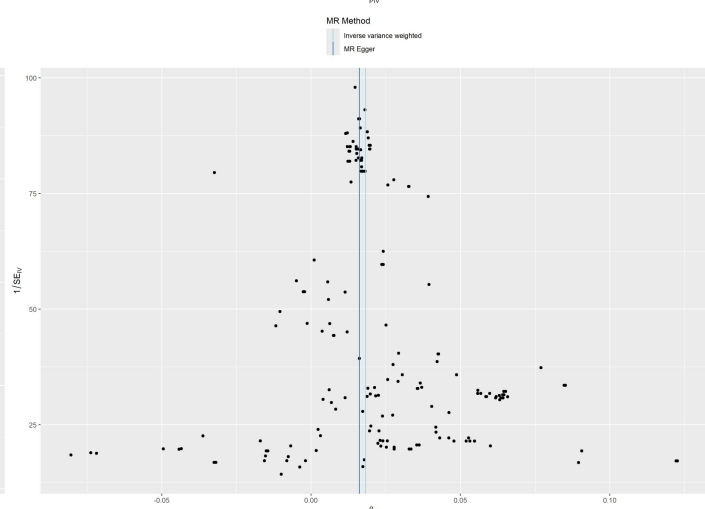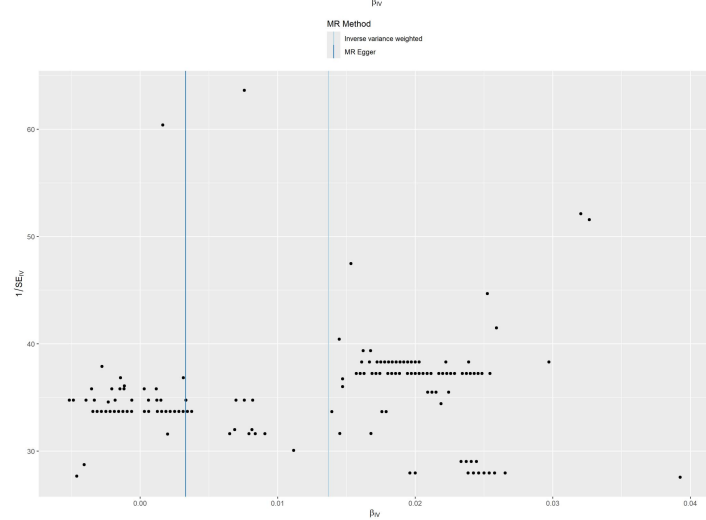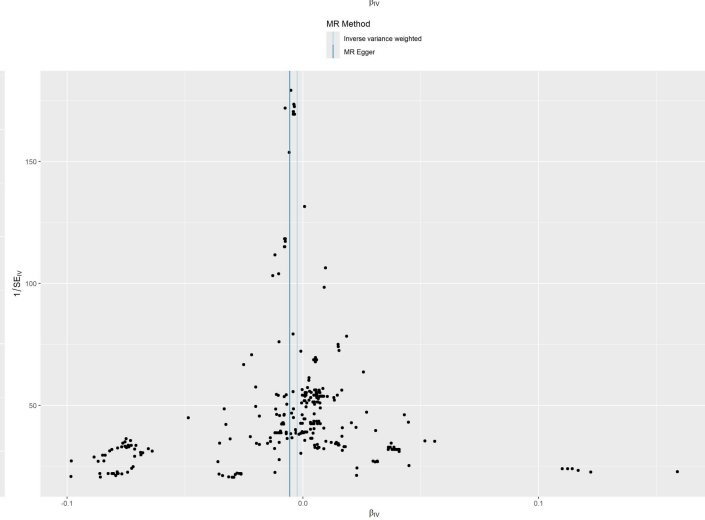

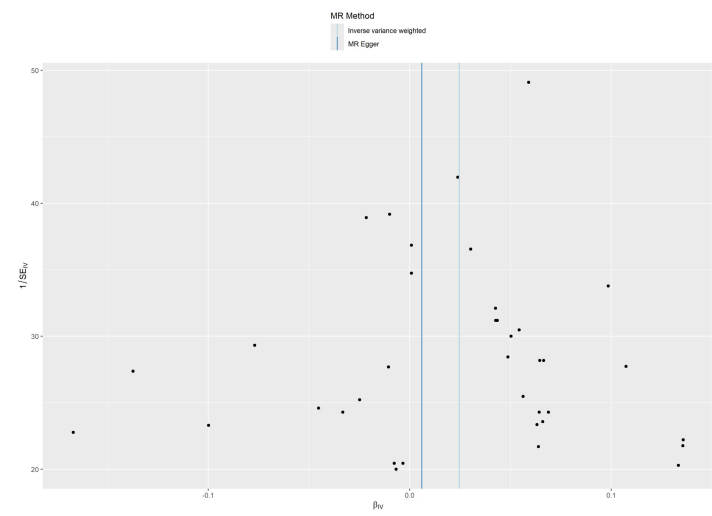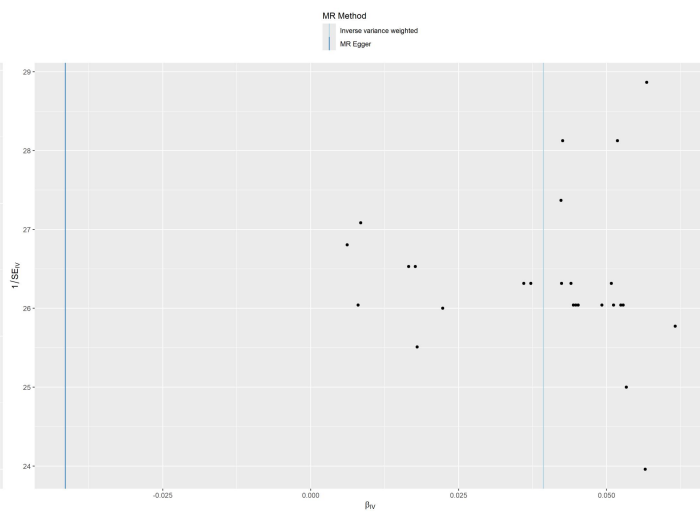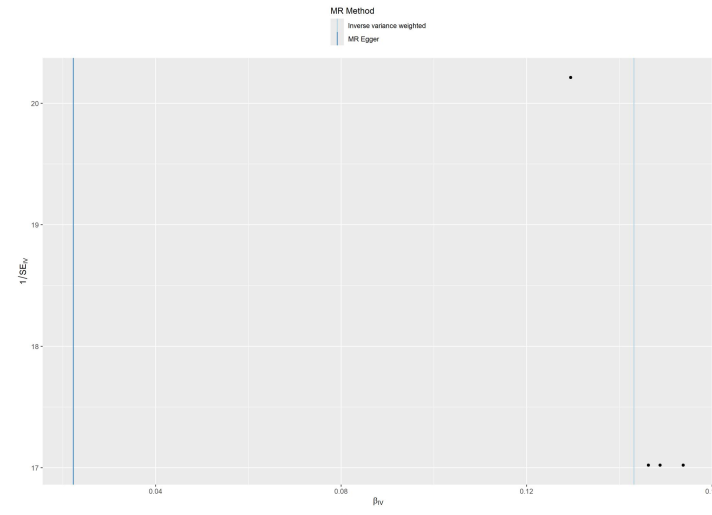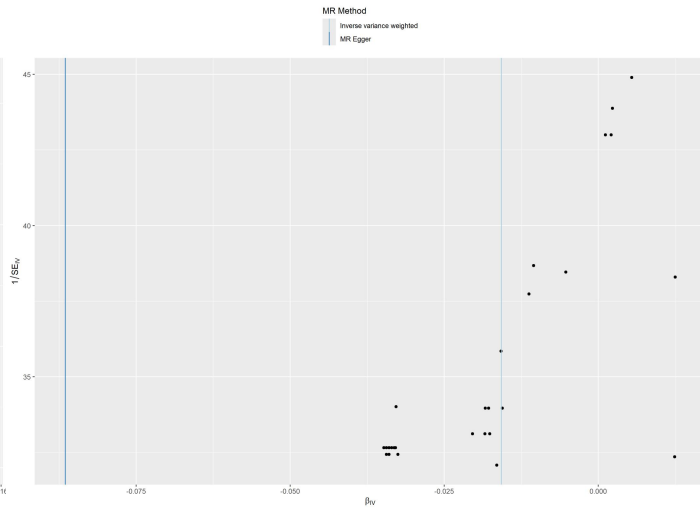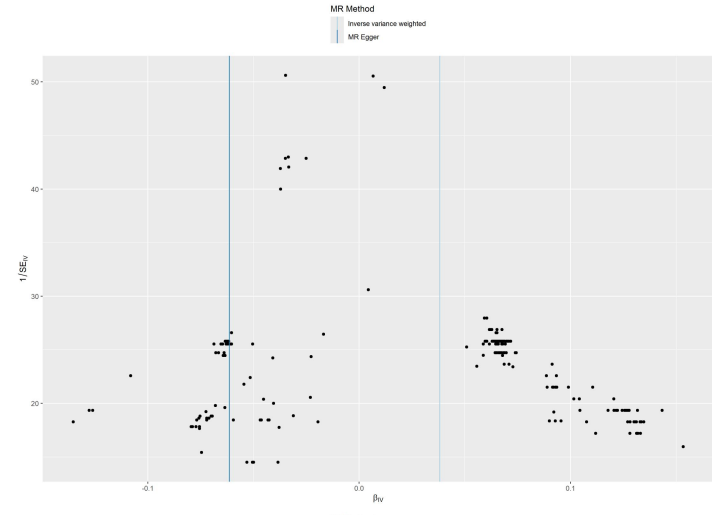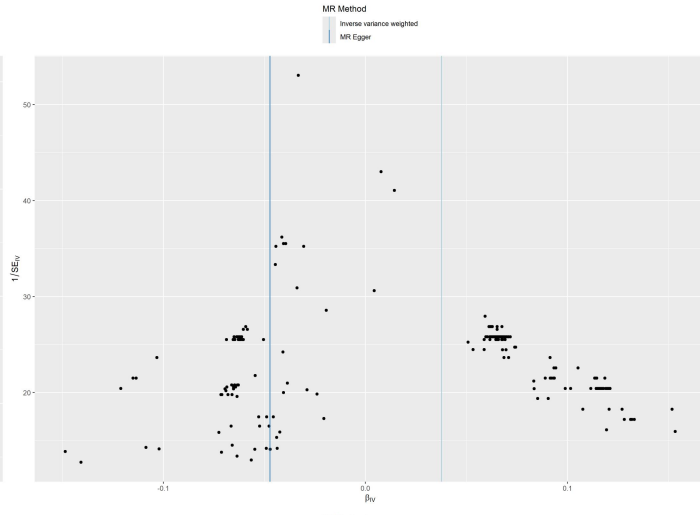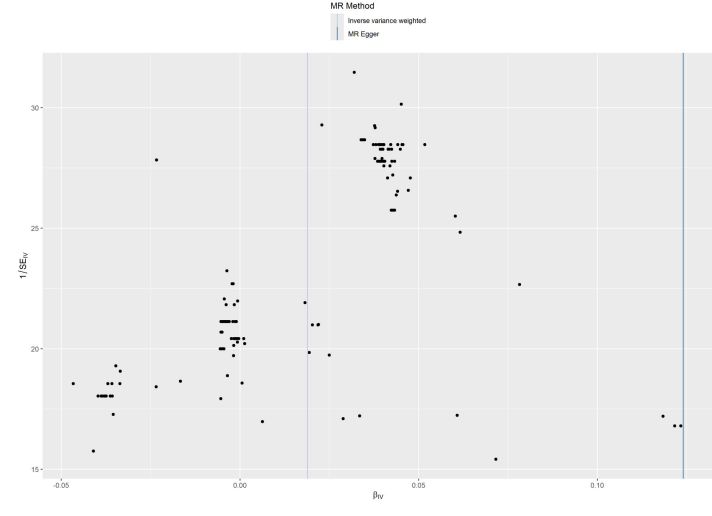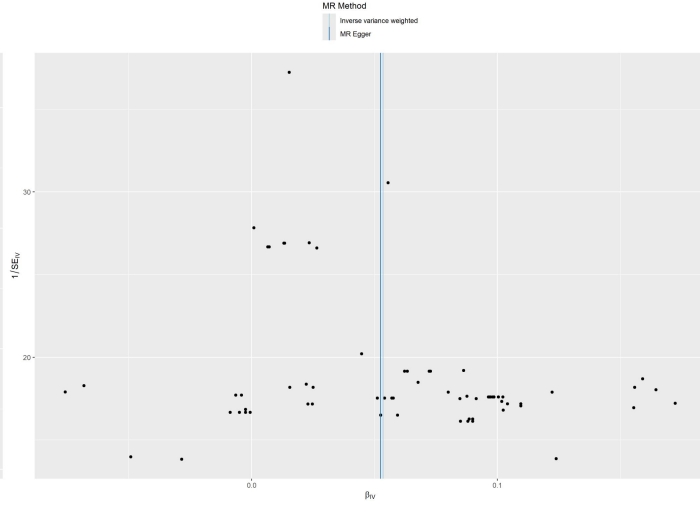

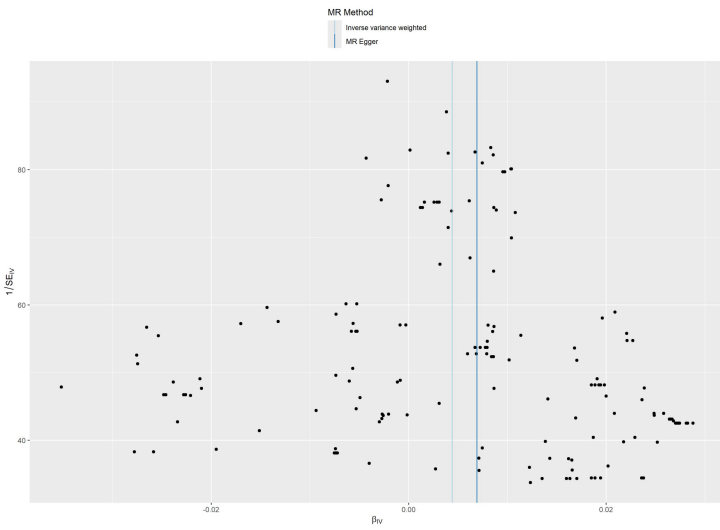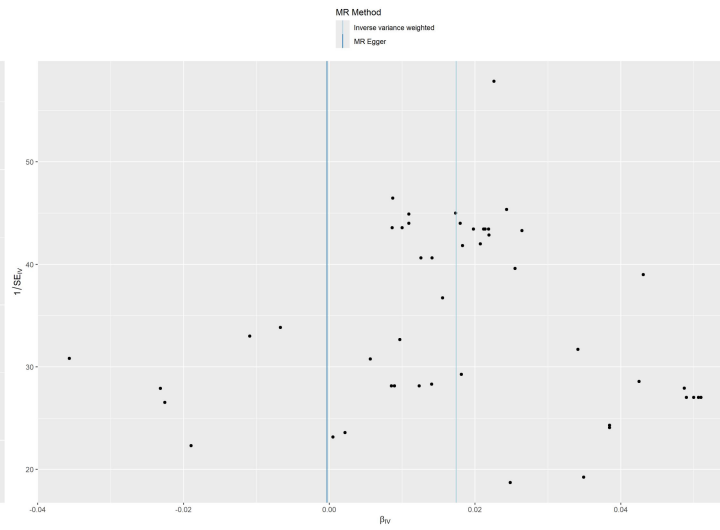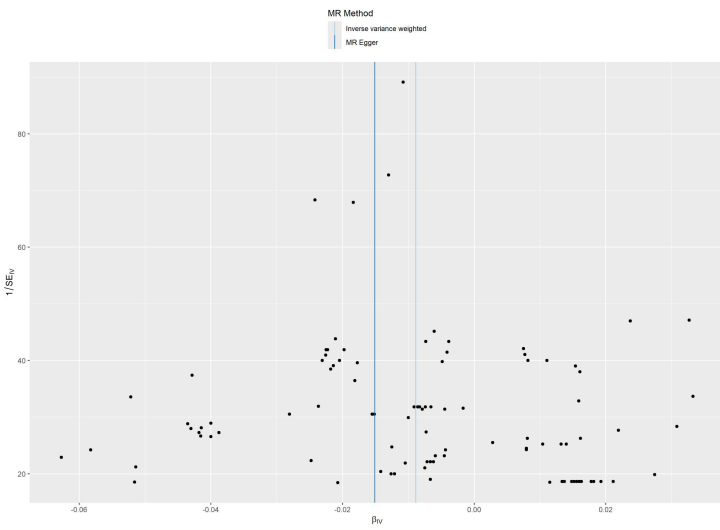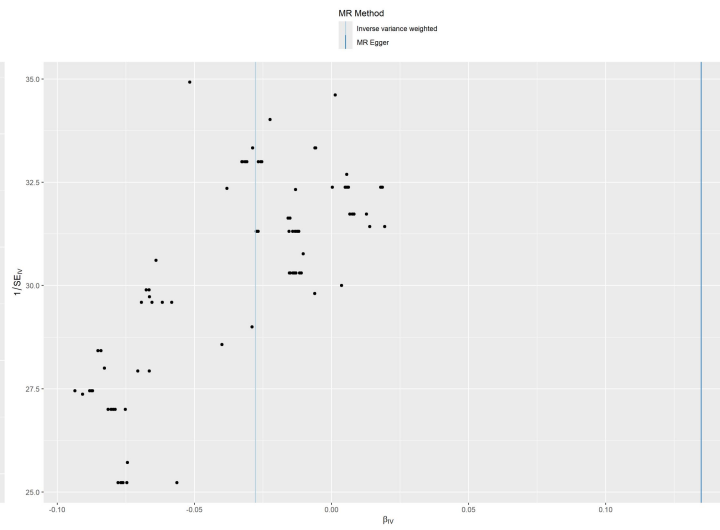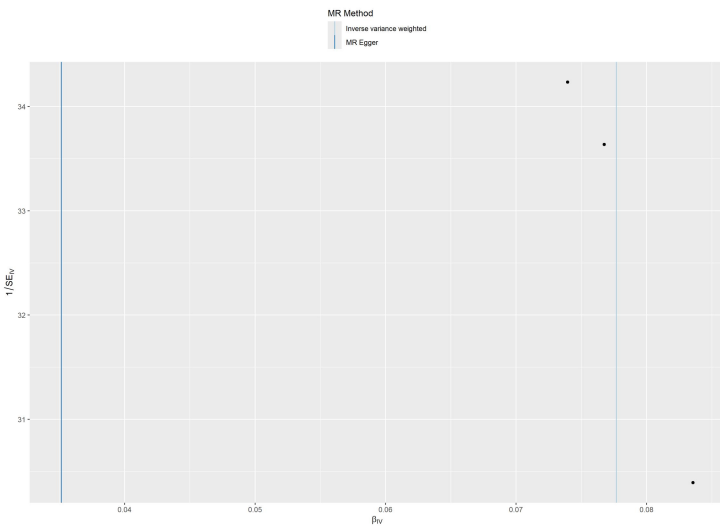

Supplement: Supplementary file 1 [file ijms-26-00283-s001.zip › Supplement File S2. knee OA funnel plots.pdf]

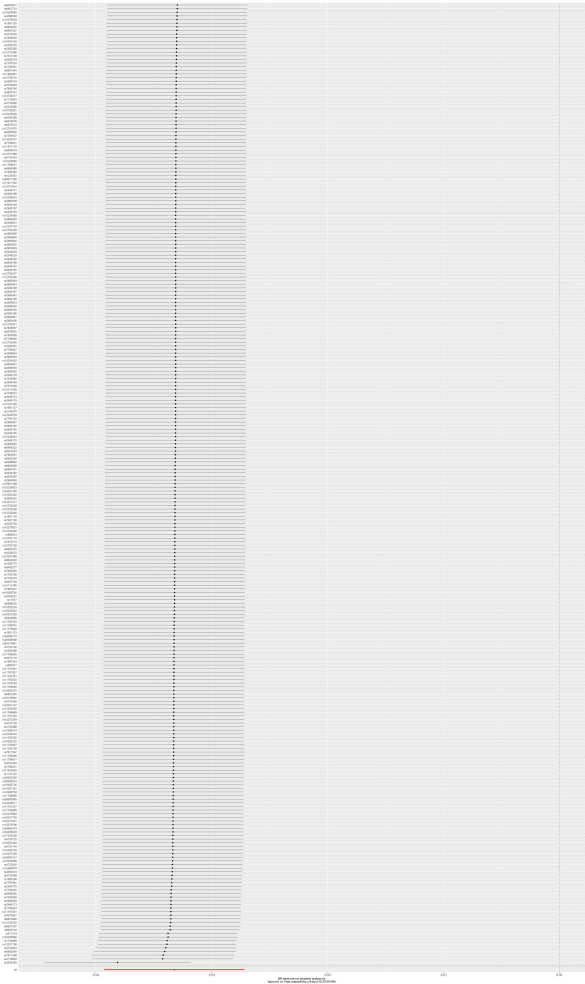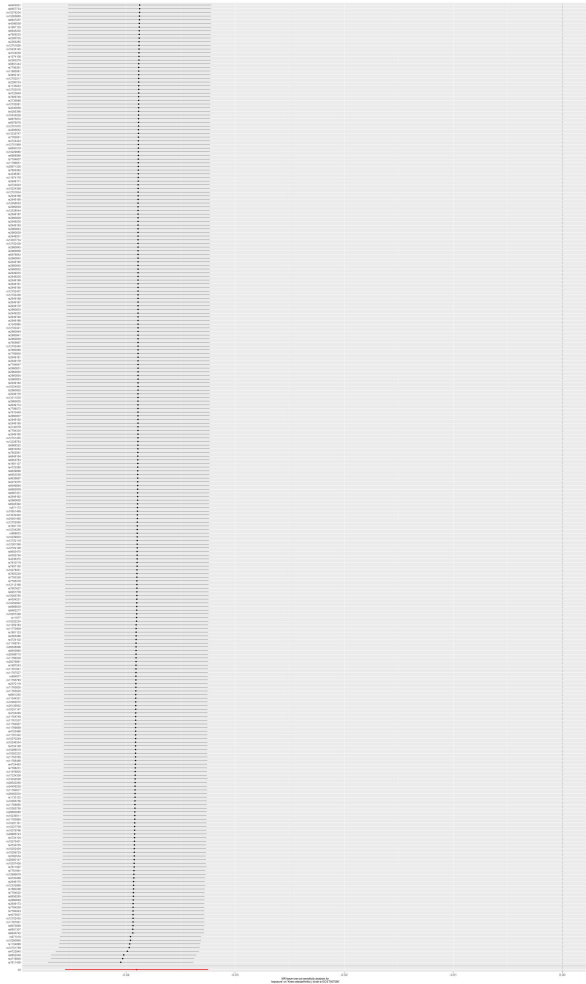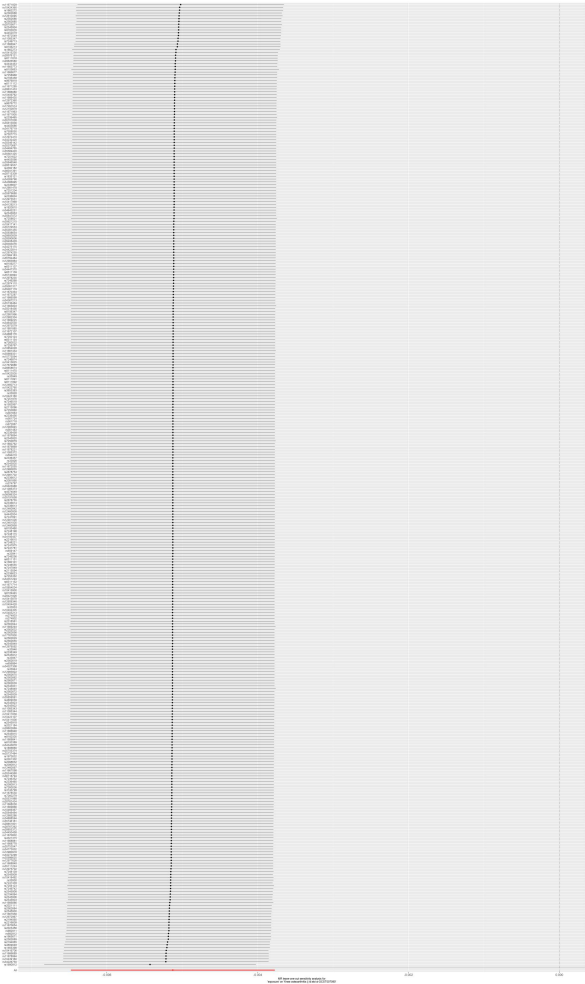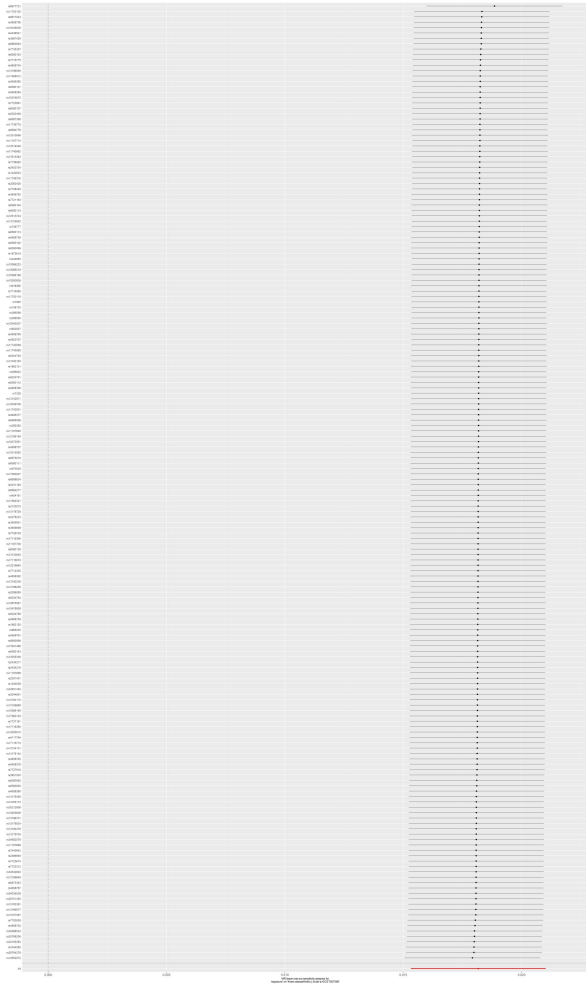

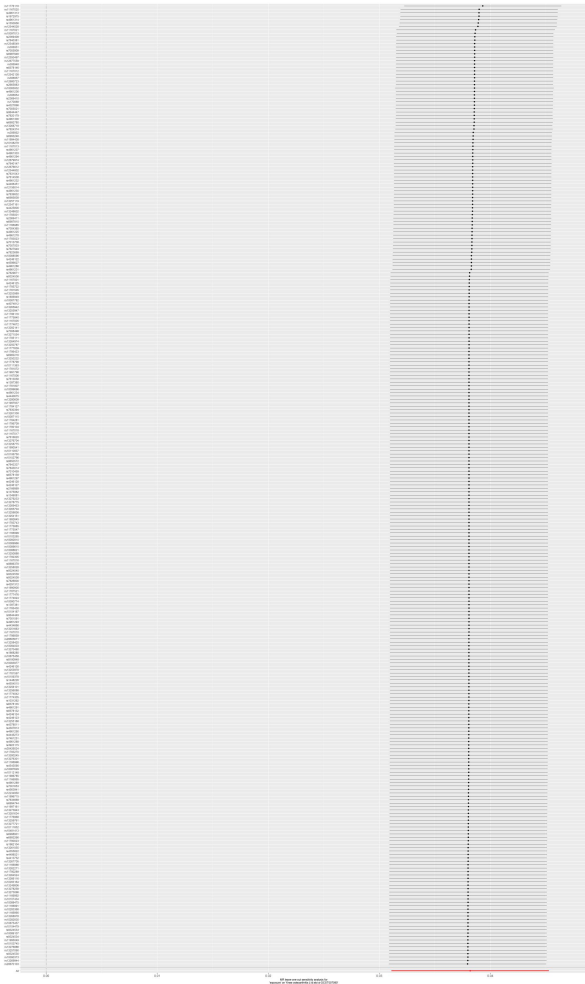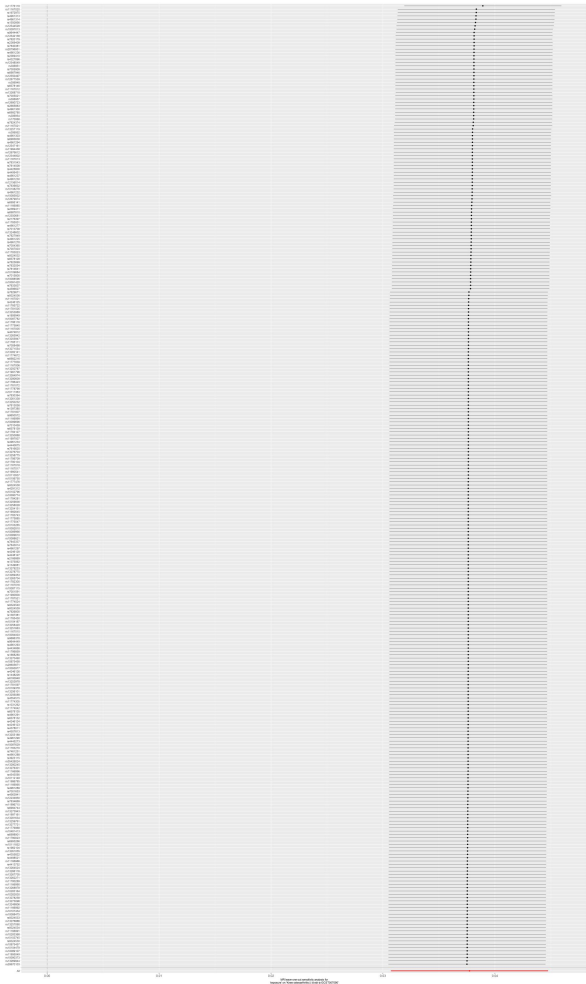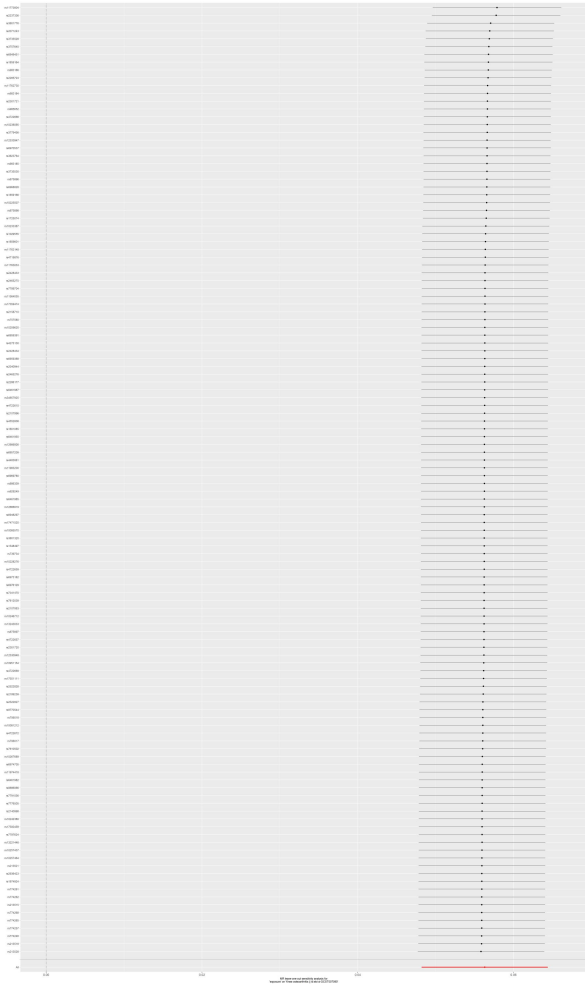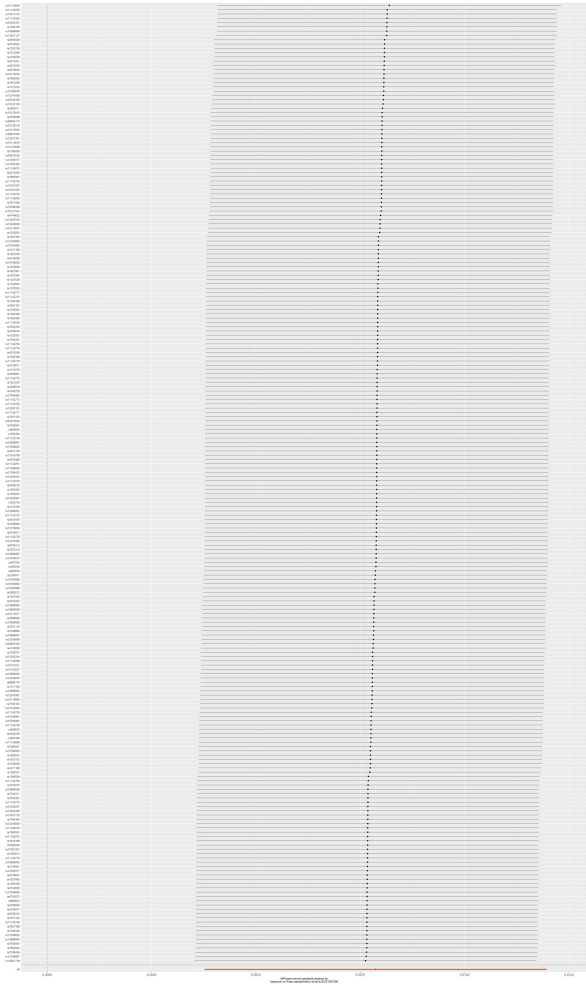

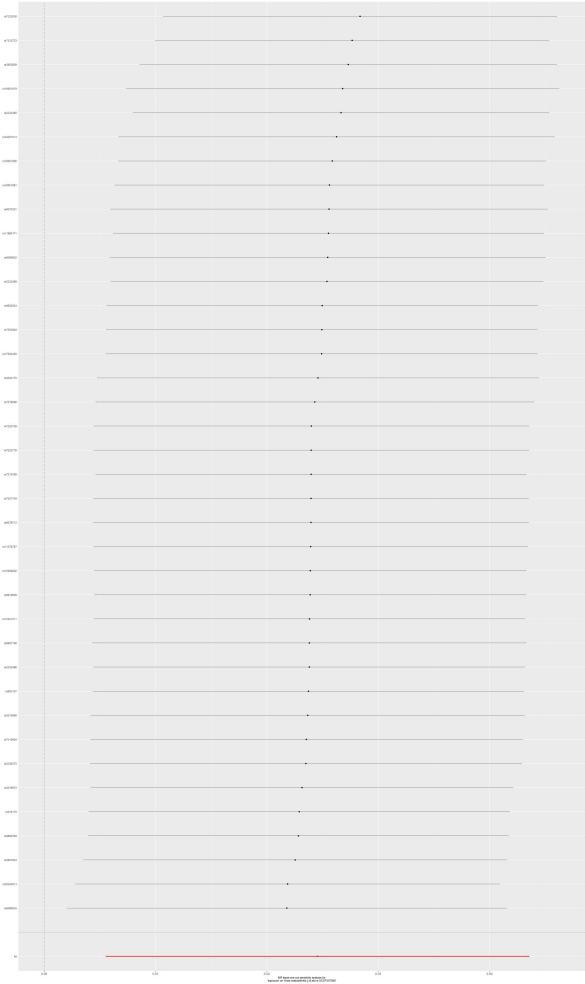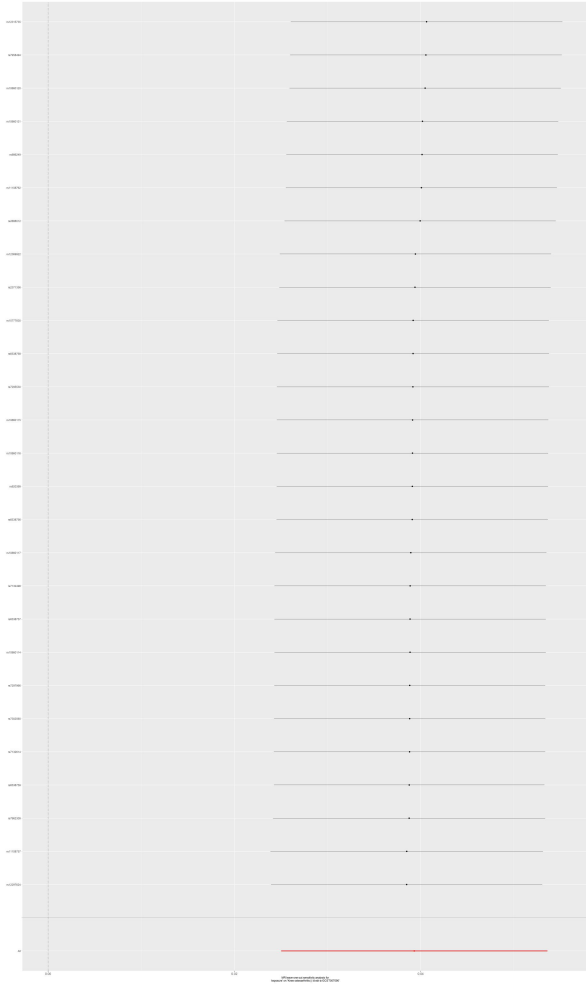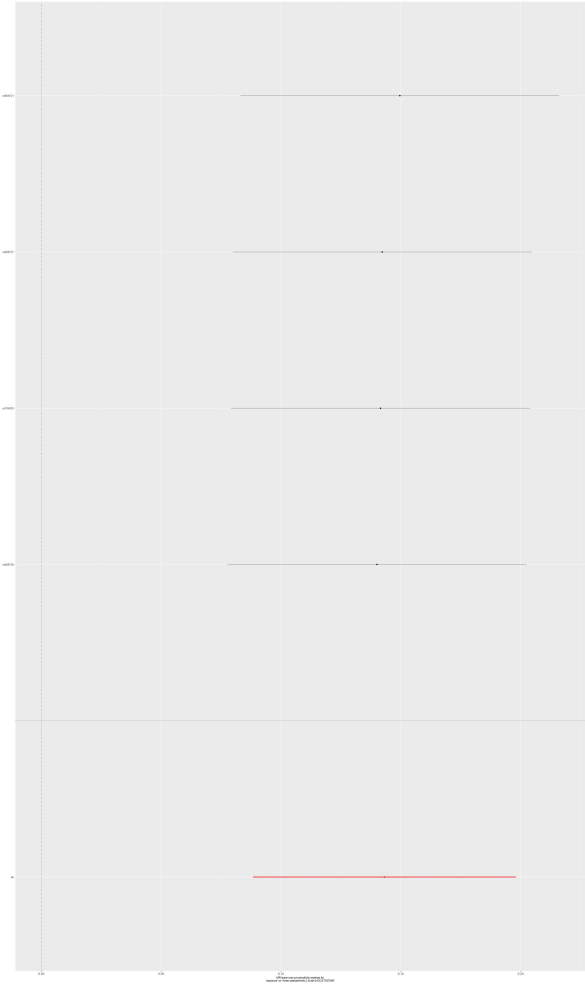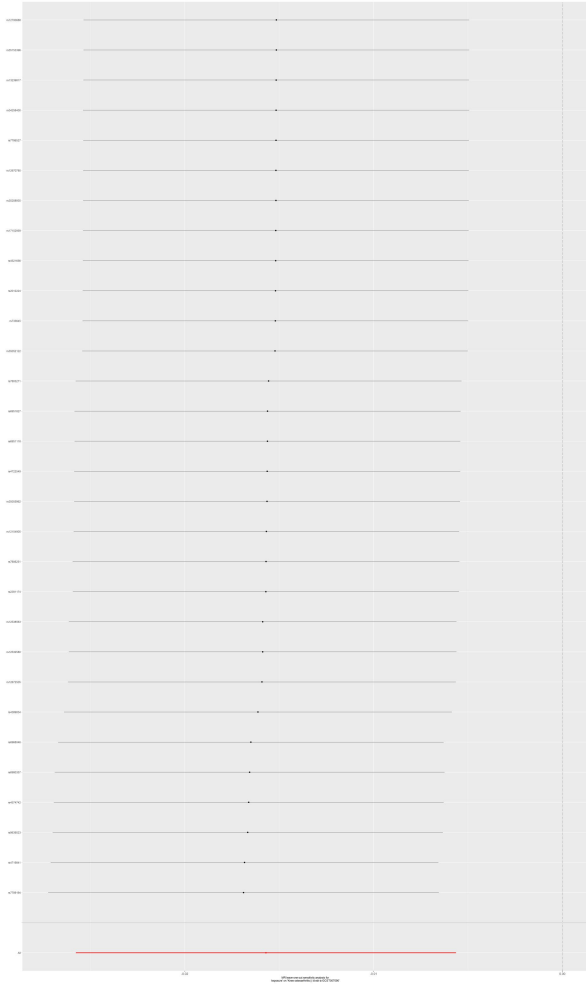

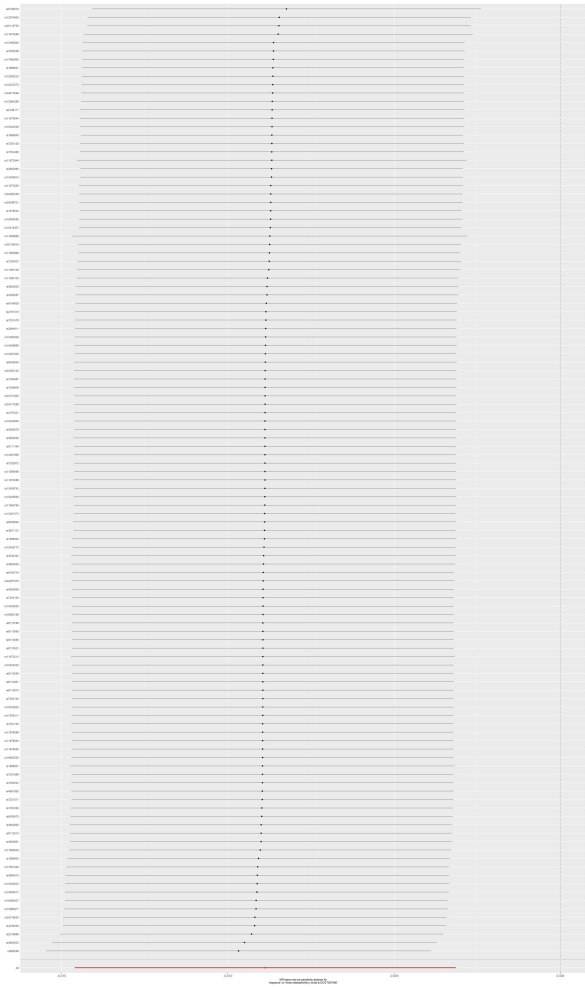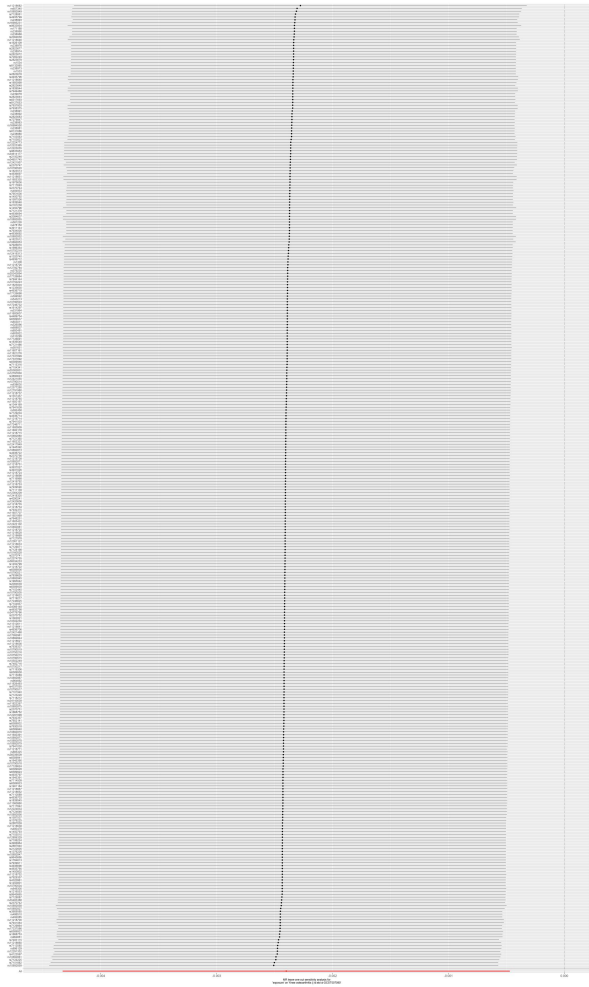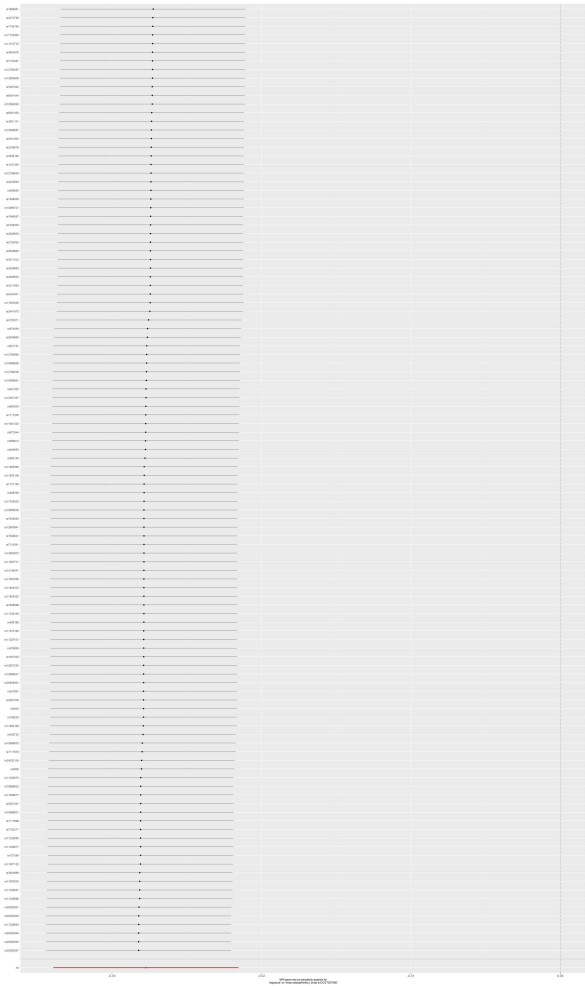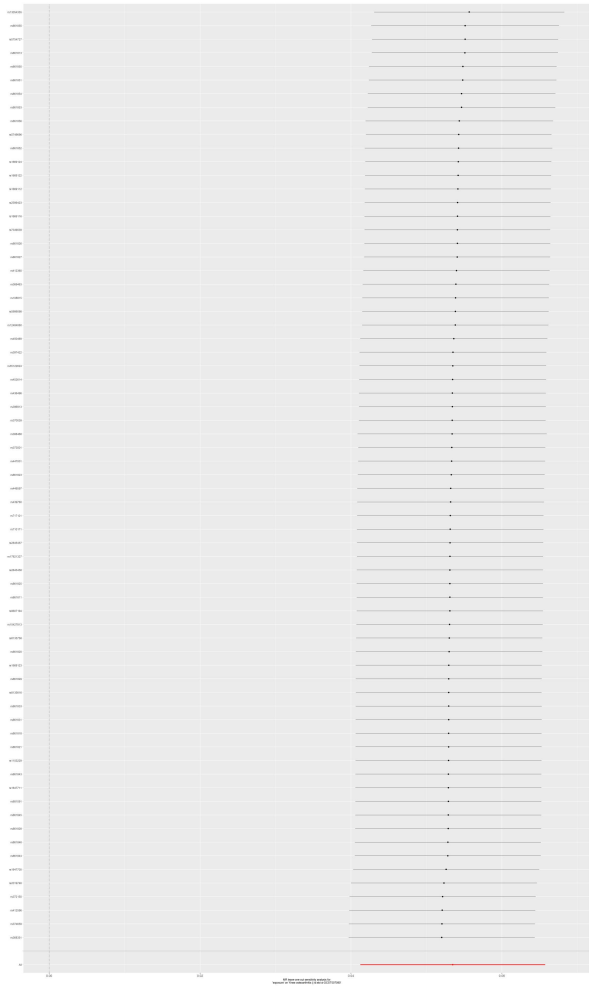

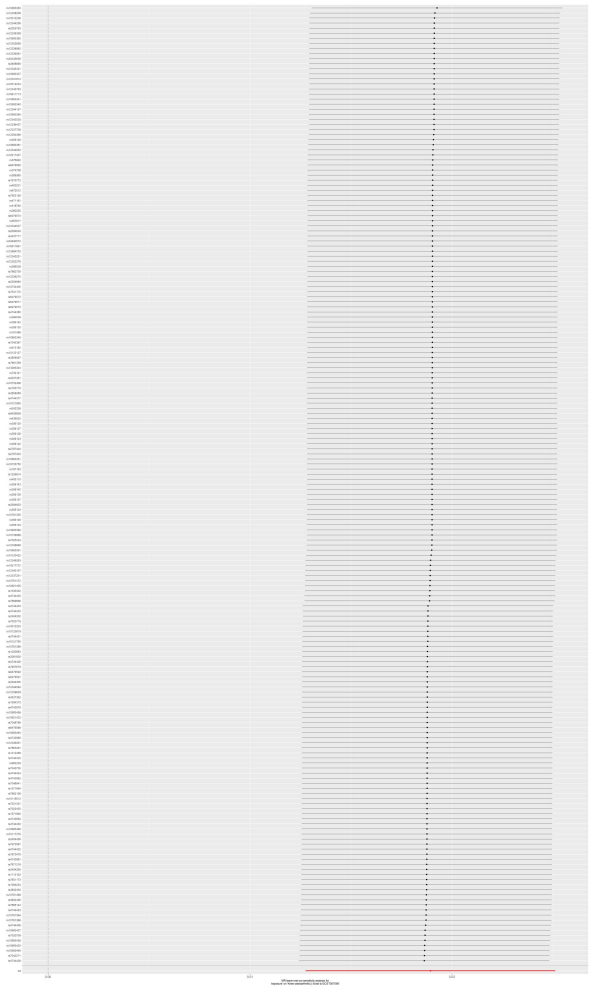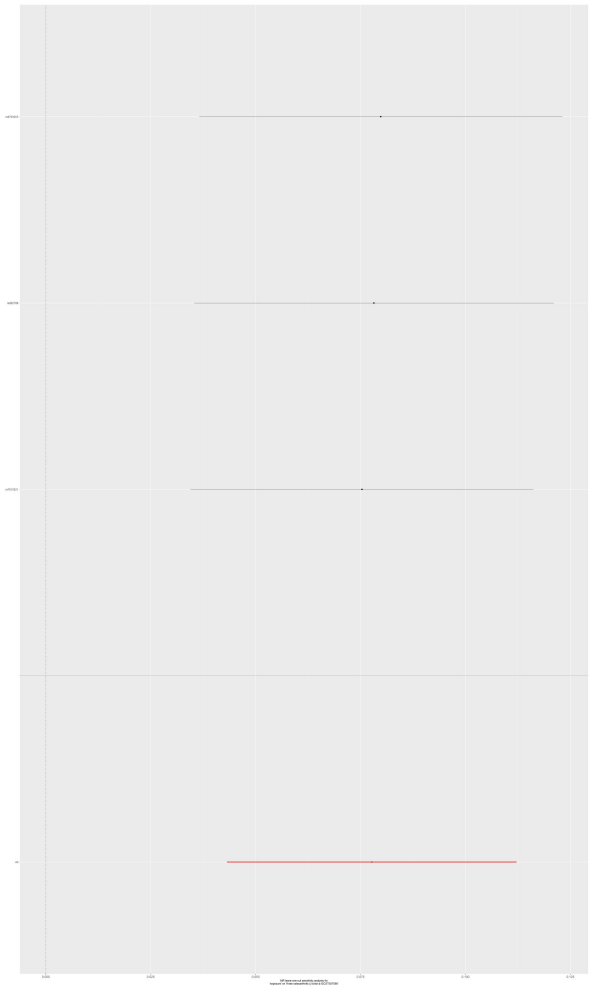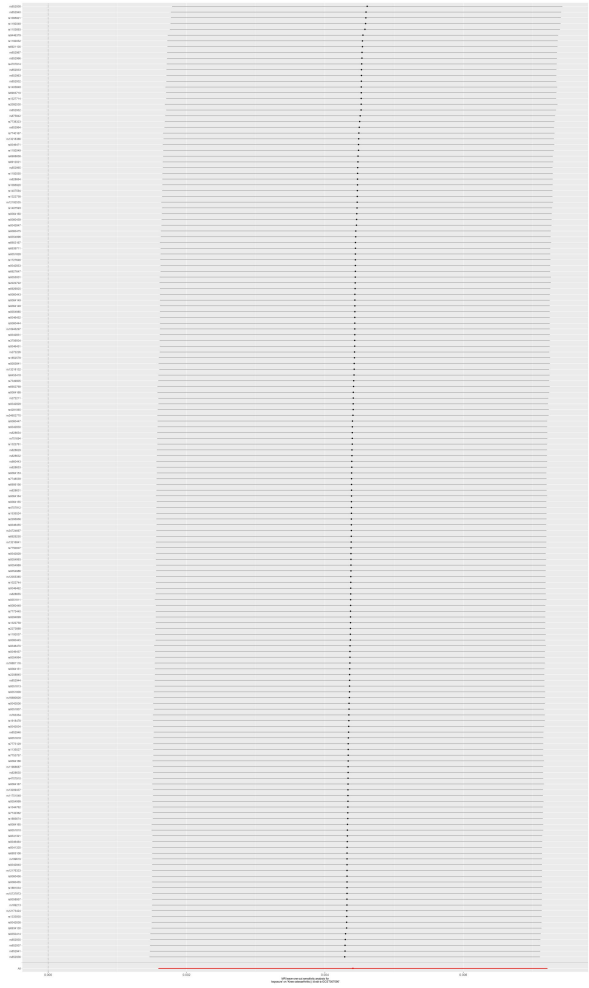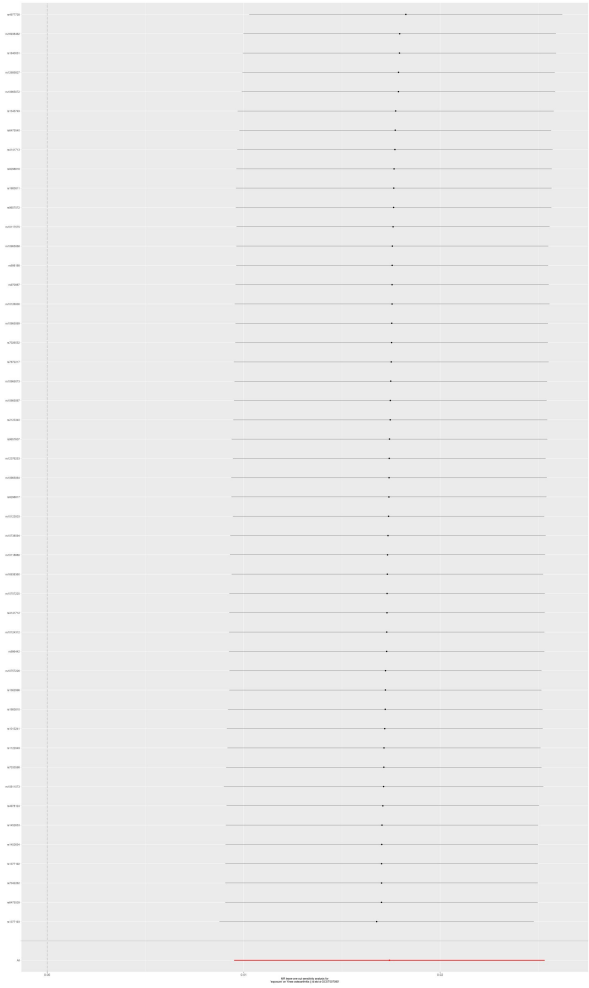

|      |  |  |  |  |  |
|------|--|--|--|--|--|
| 2000 |  |  |  |  |  |
| 2001 |  |  |  |  |  |
| 2002 |  |  |  |  |  |
| 2003 |  |  |  |  |  |
| 2004 |  |  |  |  |  |
| 2005 |  |  |  |  |  |
| 2006 |  |  |  |  |  |
| 2007 |  |  |  |  |  |
| 2008 |  |  |  |  |  |
| 2009 |  |  |  |  |  |
| 2010 |  |  |  |  |  |
| 2011 |  |  |  |  |  |
| 2012 |  |  |  |  |  |
| 2013 |  |  |  |  |  |
| 2014 |  |  |  |  |  |
| 2015 |  |  |  |  |  |
| 2016 |  |  |  |  |  |
| 2017 |  |  |  |  |  |
| 2018 |  |  |  |  |  |
| 2019 |  |  |  |  |  |
| 2020 |  |  |  |  |  |
| 2021 |  |  |  |  |  |
| 2022 |  |  |  |  |  |
| 2023 |  |  |  |  |  |
| 2024 |  |  |  |  |  |
| 2025 |  |  |  |  |  |
| 2026 |  |  |  |  |  |
| 2027 |  |  |  |  |  |
| 2028 |  |  |  |  |  |
| 2029 |  |  |  |  |  |
| 2030 |  |  |  |  |  |
| 2031 |  |  |  |  |  |
| 2032 |  |  |  |  |  |
| 2033 |  |  |  |  |  |
| 2034 |  |  |  |  |  |
| 2035 |  |  |  |  |  |
| 2036 |  |  |  |  |  |
| 2037 |  |  |  |  |  |
| 2038 |  |  |  |  |  |
| 2039 |  |  |  |  |  |
| 2040 |  |  |  |  |  |
| 2041 |  |  |  |  |  |
| 2042 |  |  |  |  |  |
| 2043 |  |  |  |  |  |
| 2044 |  |  |  |  |  |
| 2045 |  |  |  |  |  |
| 2046 |  |  |  |  |  |
| 2047 |  |  |  |  |  |
| 2048 |  |  |  |  |  |
| 2049 |  |  |  |  |  |
| 2050 |  |  |  |  |  |
| 2051 |  |  |  |  |  |
| 2052 |  |  |  |  |  |
| 2053 |  |  |  |  |  |
| 2054 |  |  |  |  |  |
| 2055 |  |  |  |  |  |
| 2056 |  |  |  |  |  |
| 2057 |  |  |  |  |  |
| 2058 |  |  |  |  |  |
| 2059 |  |  |  |  |  |
| 2060 |  |  |  |  |  |
| 2061 |  |  |  |  |  |
| 2062 |  |  |  |  |  |
| 2063 |  |  |  |  |  |
| 2064 |  |  |  |  |  |
| 2065 |  |  |  |  |  |
| 2066 |  |  |  |  |  |
| 2067 |  |  |  |  |  |
| 2068 |  |  |  |  |  |
| 2069 |  |  |  |  |  |
| 2070 |  |  |  |  |  |
| 2071 |  |  |  |  |  |
| 2072 |  |  |  |  |  |
| 2073 |  |  |  |  |  |
| 2074 |  |  |  |  |  |
| 2075 |  |  |  |  |  |
| 2076 |  |  |  |  |  |
| 2077 |  |  |  |  |  |
| 2078 |  |  |  |  |  |
| 2079 |  |  |  |  |  |
| 2080 |  |  |  |  |  |
| 2081 |  |  |  |  |  |
| 2082 |  |  |  |  |  |
| 2083 |  |  |  |  |  |
| 2084 |  |  |  |  |  |
| 2085 |  |  |  |  |  |
| 2086 |  |  |  |  |  |
| 2087 |  |  |  |  |  |
| 2088 |  |  |  |  |  |
| 2089 |  |  |  |  |  |
| 2090 |  |  |  |  |  |
| 2091 |  |  |  |  |  |
| 2092 |  |  |  |  |  |
| 2093 |  |  |  |  |  |
| 2094 |  |  |  |  |  |
| 2095 |  |  |  |  |  |
| 2096 |  |  |  |  |  |
| 2097 |  |  |  |  |  |
| 2098 |  |  |  |  |  |
| 2099 |  |  |  |  |  |
| 2100 |  |  |  |  |  |
| 2101 |  |  |  |  |  |
| 2102 |  |  |  |  |  |
| 2103 |  |  |  |  |  |
| 2104 |  |  |  |  |  |
| 2105 |  |  |  |  |  |
| 2106 |  |  |  |  |  |
| 2107 |  |  |  |  |  |
| 2108 |  |  |  |  |  |
| 2109 |  |  |  |  |  |
| 2110 |  |  |  |  |  |
| 2111 |  |  |  |  |  |
| 2112 |  |  |  |  |  |
| 2113 |  |  |  |  |  |
| 2114 |  |  |  |  |  |
| 2115 |  |  |  |  |  |
| 2116 |  |  |  |  |  |
| 2117 |  |  |  |  |  |
| 2118 |  |  |  |  |  |
| 2119 |  |  |  |  |  |
| 2120 |  |  |  |  |  |
| 2121 |  |  |  |  |  |
| 2122 |  |  |  |  |  |
| 2123 |  |  |  |  |  |
| 2124 |  |  |  |  |  |
| 2125 |  |  |  |  |  |
| 2126 |  |  |  |  |  |
| 2127 |  |  |  |  |  |
| 2128 |  |  |  |  |  |
| 2129 |  |  |  |  |  |
| 2130 |  |  |  |  |  |
| 2131 |  |  |  |  |  |
| 2132 |  |  |  |  |  |
| 2133 |  |  |  |  |  |
| 2134 |  |  |  |  |  |
| 2135 |  |  |  |  |  |
| 2136 |  |  |  |  |  |
| 2137 |  |  |  |  |  |
| 2138 |  |  |  |  |  |
| 2139 |  |  |  |  |  |
| 2140 |  |  |  |  |  |
| 2141 |  |  |  |  |  |
| 2142 |  |  |  |  |  |
| 2143 |  |  |  |  |  |
| 2144 |  |  |  |  |  |
| 2145 |  |  |  |  |  |
| 2146 |  |  |  |  |  |
| 2147 |  |  |  |  |  |
| 2148 |  |  |  |  |  |
| 2149 |  |  |  |  |  |
| 2150 |  |  |  |  |  |
| 2151 |  |  |  |  |  |
| 2152 |  |  |  |  |  |
| 2153 |  |  |  |  |  |
| 2154 |  |  |  |  |  |
| 2155 |  |  |  |  |  |
| 2156 |  |  |  |  |  |
| 2157 |  |  |  |  |  |
| 2158 |  |  |  |  |  |
| 2159 |  |  |  |  |  |
| 2160 |  |  |  |  |  |
| 2161 |  |  |  |  |  |
| 2162 |  |  |  |  |  |
| 2163 |  |  |  |  |  |
| 2164 |  |  |  |  |  |
| 2165 |  |  |  |  |  |
| 2166 |  |  |  |  |  |
| 2167 |  |  |  |  |  |
| 2168 |  |  |  |  |  |
| 2169 |  |  |  |  |  |
| 2170 |  |  |  |  |  |
| 2171 |  |  |  |  |  |
| 2172 |  |  |  |  |  |
| 2173 |  |  |  |  |  |
| 2174 |  |  |  |  |  |
| 2175 |  |  |  |  |  |
| 2176 |  |  |  |  |  |
| 2177 |  |  |  |  |  |
| 2178 |  |  |  |  |  |
| 2179 |  |  |  |  |  |
| 2180 |  |  |  |  |  |
| 2181 |  |  |  |  |  |
| 2182 |  |  |  |  |  |
| 2183 |  |  |  |  |  |
| 2184 |  |  |  |  |  |
| 2185 |  |  |  |  |  |
| 2186 |  |  |  |  |  |
| 2187 |  |  |  |  |  |
| 2188 |  |  |  |  |  |
| 2189 |  |  |  |  |  |
| 2190 |  |  |  |  |  |
| 2191 |  |  |  |  |  |
| 2192 |  |  |  |  |  |
| 2193 |  |  |  |  |  |
| 2194 |  |  |  |  |  |
| 2195 |  |  |  |  |  |
| 2196 |  |  |  |  |  |
| 2197 |  |  |  |  |  |
| 2198 |  |  |  |  |  |
| 2199 |  |  |  |  |  |
| 2200 |  |  |  |  |  |
| 2201 |  |  |  |  |  |
| 2202 |  |  |  |  |  |
| 2203 |  |  |  |  |  |
| 2204 |  |  |  |  |  |
| 2205 |  |  |  |  |  |
| 2206 |  |  |  |  |  |
| 2207 |  |  |  |  |  |
| 2208 |  |  |  |  |  |
| 2209 |  |  |  |  |  |
| 2210 |  |  |  |  |  |
| 2211 |  |  |  |  |  |
| 2212 |  |  |  |  |  |
| 2213 |  |  |  |  |  |
| 2214 |  |  |  |  |  |
| 2215 |  |  |  |  |  |
| 2216 |  |  |  |  |  |
| 2217 |  |  |  |  |  |
| 2218 |  |  |  |  |  |
| 2219 |  |  |  |  |  |
| 2220 |  |  |  |  |  |
| 2221 |  |  |  |  |  |
| 2222 |  |  |  |  |  |
| 2223 |  |  |  |  |  |
| 2224 |  |  |  |  |  |
| 2225 |  |  |  |  |  |
| 2226 |  |  |  |  |  |
| 2227 |  |  |  |  |  |
| 2228 |  |  |  |  |  |
| 2229 |  |  |  |  |  |
| 2230 |  |  |  |  |  |
| 2231 |  |  |  |  |  |
| 2232 |  |  |  |  |  |
| 2233 |  |  |  |  |  |
| 2234 |  |  |  |  |  |
| 2235 |  |  |  |  |  |
| 2236 |  |  |  |  |  |
| 2237 |  |  |  |  |  |
| 2238 |  |  |  |  |  |
| 2239 |  |  |  |  |  |
| 2240 |  |  |  |  |  |
| 2241 |  |  |  |  |  |
| 2242 |  |  |  |  |  |
| 2243 |  |  |  |  |  |
| 2244 |  |  |  |  |  |
| 2245 |  |  |  |  |  |
| 2246 |  |  |  |  |  |
| 2247 |  |  |  |  |  |
| 2248 |  |  |  |  |  |
| 2249 |  |  |  |  |  |
| 2250 |  |  |  |  |  |
| 2251 |  |  |  |  |  |
| 2252 |  |  |  |  |  |
| 2253 |  |  |  |  |  |
| 2254 |  |  |  |  |  |
| 2255 |  |  |  |  |  |
| 2256 |  |  |  |  |  |
| 2257 |  |  |  |  |  |
| 2258 |  |  |  |  |  |
| 2259 |  |  |  |  |  |
| 2260 |  |  |  |  |  |
| 2261 |  |  |  |  |  |
| 2262 |  |  |  |  |  |
| 2263 |  |  |  |  |  |
| 2264 |  |  |  |  |  |
| 2265 |  |  |  |  |  |
| 2266 |  |  |  |  |  |
| 2267 |  |  |  |  |  |
| 2268 |  |  |  |  |  |
| 2269 |  |  |  |  |  |
| 2270 |  |  |  |  |  |
| 2271 |  |  |  |  |  |
| 2272 |  |  |  |  |  |
| 2273 |  |  |  |  |  |
| 2274 |  |  |  |  |  |
| 2275 |  |  |  |  |  |
| 2276 |  |  |  |  |  |
| 2277 |  |  |  |  |  |
| 2278 |  |  |  |  |  |
| 2279 |  |  |  |  |  |
| 2280 |  |  |  |  |  |
| 2281 |  |  |  |  |  |
| 2282 |  |  |  |  |  |
| 2283 |  |  |  |  |  |
| 2284 |  |  |  |  |  |
| 2285 |  |  |  |  |  |
| 2286 |  |  |  |  |  |
| 2287 |  |  |  |  |  |
| 2288 |  |  |  |  |  |
| 2289 |  |  |  |  |  |
| 2290 |  |  |  |  |  |
| 2291 |  |  |  |  |  |
| 2292 |  |  |  |  |  |
| 2293 |  |  |  |  |  |
| 2294 |  |  |  |  |  |
| 2295 |  |  |  |  |  |
| 2296 |  |  |  |  |  |
| 2297 |  |  |  |  |  |
| 2298 |  |  |  |  |  |
| 2299 |  |  |  |  |  |
| 2300 |  |  |  |  |  |
| 2301 |  |  |  |  |  |
| 2302 |  |  |  |  |  |
| 2303 |  |  |  |  |  |
| 2304 |  |  |  |  |  |
| 2305 |  |  |  |  |  |
| 2306 |  |  |  |  |  |
| 2307 |  |  |  |  |  |
| 2308 |  |  |  |  |  |
| 2309 |  |  |  |  |  |
| 2310 |  |  |  |  |  |
| 2311 |  |  |  |  |  |
| 2312 |  |  |  |  |  |
| 2313 |  |  |  |  |  |
| 2314 |  |  |  |  |  |
| 2315 |  |  |  |  |  |
| 2316 |  |  |  |  |  |
| 2317 |  |  |  |  |  |
| 2318 |  |  |  |  |  |
| 2319 |  |  |  |  |  |
| 2320 |  |  |  |  |  |
| 2321 |  |  |  |  |  |
| 2322 |  |  |  |  |  |
| 2323 |  |  |  |  |  |
| 2324 |  |  |  |  |  |
| 2325 |  |  |  |  |  |
| 2326 |  |  |  |  |  |
| 2327 |  |  |  |  |  |
| 2328 |  |  |  |  |  |
| 2329 |  |  |  |  |  |
| 2330 |  |  |  |  |  |
| 2331 |  |  |  |  |  |
| 2332 |  |  |  |  |  |
| 2333 |  |  |  |  |  |
| 2334 |  |  |  |  |  |
| 2335 |  |  |  |  |  |
| 2336 |  |  |  |  |  |
| 2337 |  |  |  |  |  |
| 2338 |  |  |  |  |  |
| 2339 |  |  |  |  |  |
| 2340 |  |  |  |  |  |
| 2341 |  |  |  |  |  |
| 2342 |  |  |  |  |  |
| 2343 |  |  |  |  |  |
| 2344 |  |  |  |  |  |
| 2345 |  |  |  |  |  |
| 2346 |  |  |  |  |  |
| 2347 |  |  |  |  |  |
| 2348 |  |  |  |  |  |
| 2349 |  |  |  |  |  |
| 2350 |  |  |  |  |  |
| 2351 |  |  |  |  |  |
| 2352 |  |  |  |  |  |
| 2353 |  |  |  |  |  |
| 2354 |  |  |  |  |  |
| 2355 |  |  |  |  |  |
| 2356 |  |  |  |  |  |
| 2357 |  |  |  |  |  |
| 2358 |  |  |  |  |  |
| 2359 |  |  |  |  |  |
| 2360 |  |  |  |  |  |
| 2361 |  |  |  |  |  |
| 2362 |  |  |  |  |  |
| 2363 |  |  |  |  |  |
| 2364 |  |  |  |  |  |
| 2365 |  |  |  |  |  |
| 2366 |  |  |  |  |  |
| 2367 |  |  |  |  |  |
| 2368 |  |  |  |  |  |
| 2369 |  |  |  |  |  |
| 2370 |  |  |  |  |  |
| 2371 |  |  |  |  |  |
| 2372 |  |  |  |  |  |
| 2373 |  |  |  |  |  |
| 2374 |  |  |  |  |  |

Supplement: Supplementary file 1 [file ijms-26-00283-s001.zip › Supplement File S3. knee OA leave-one-out forest maps.pdf]

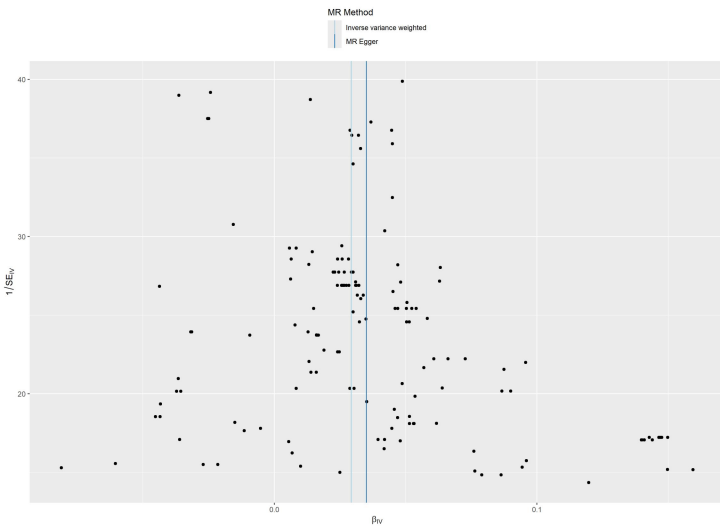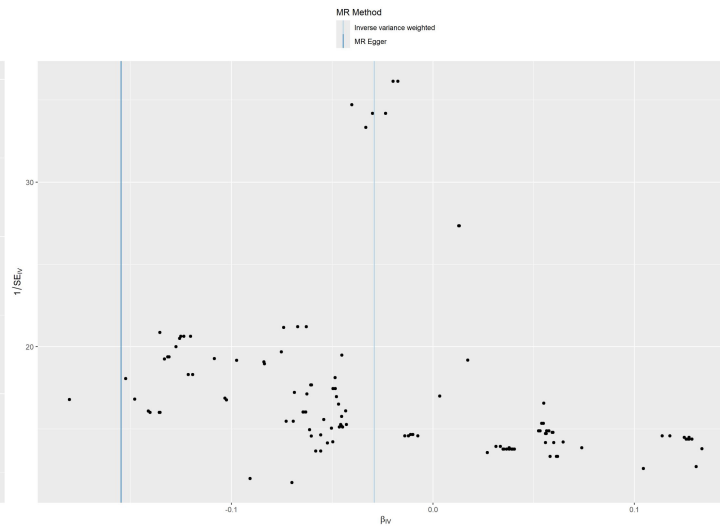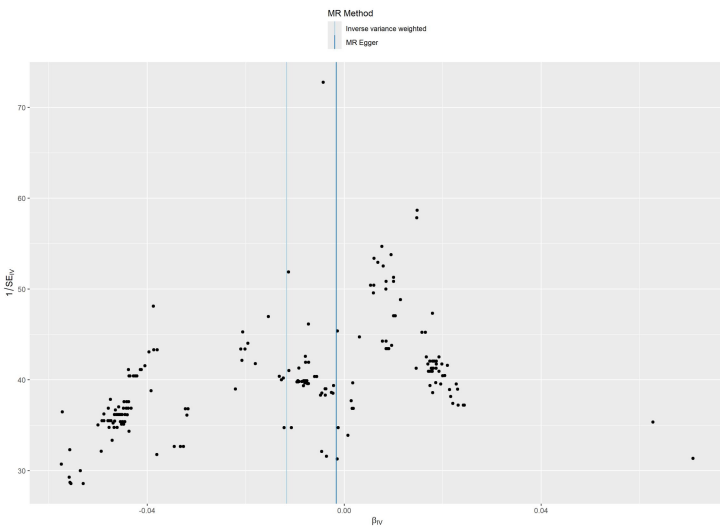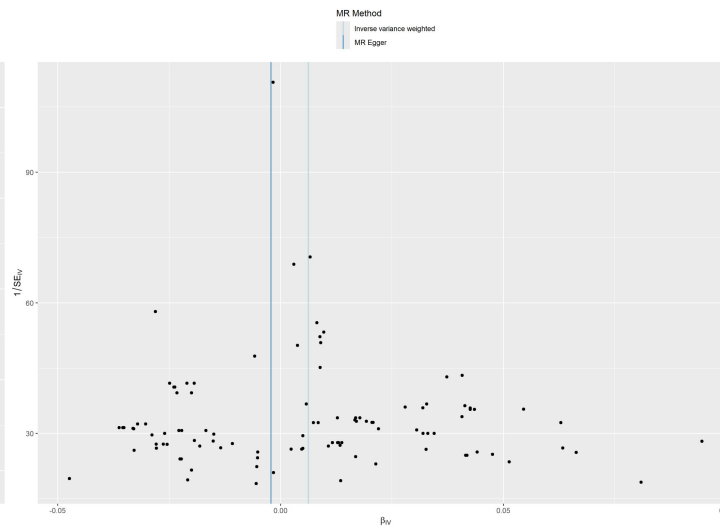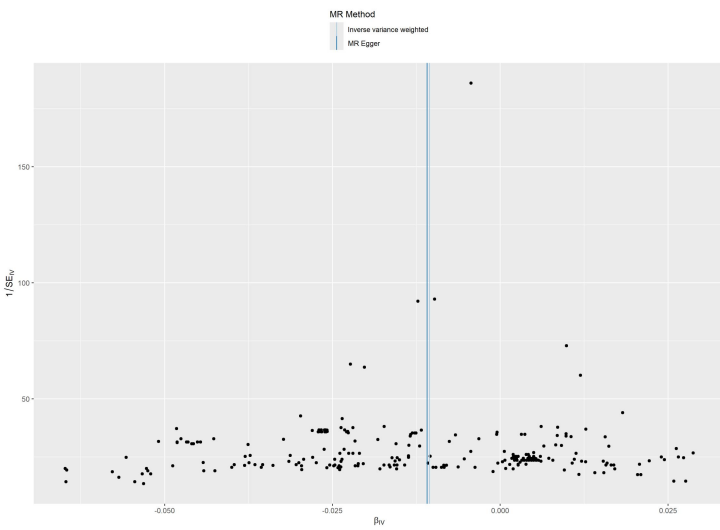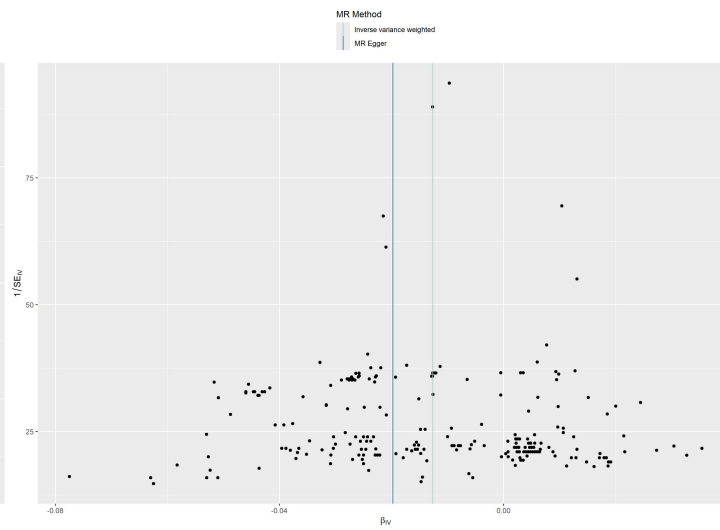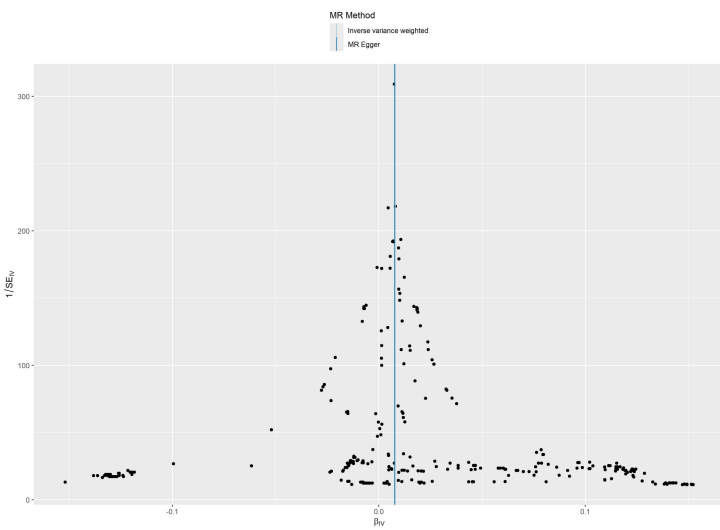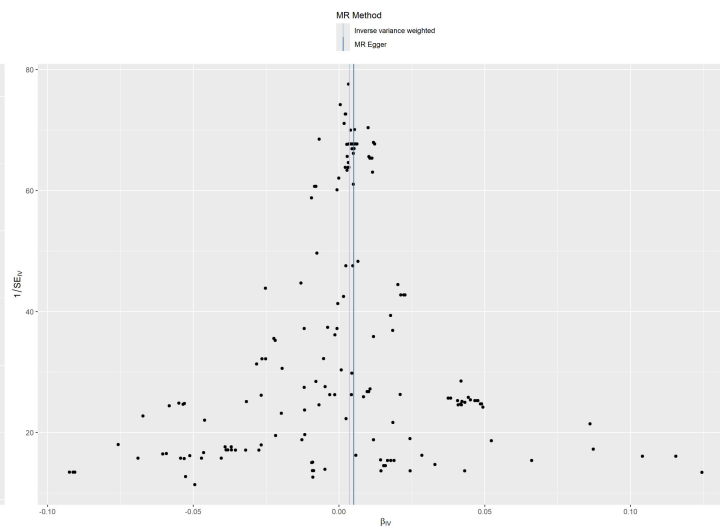

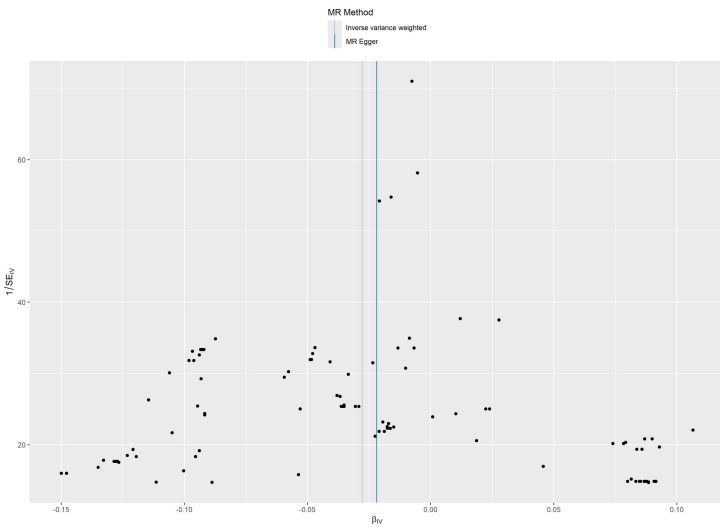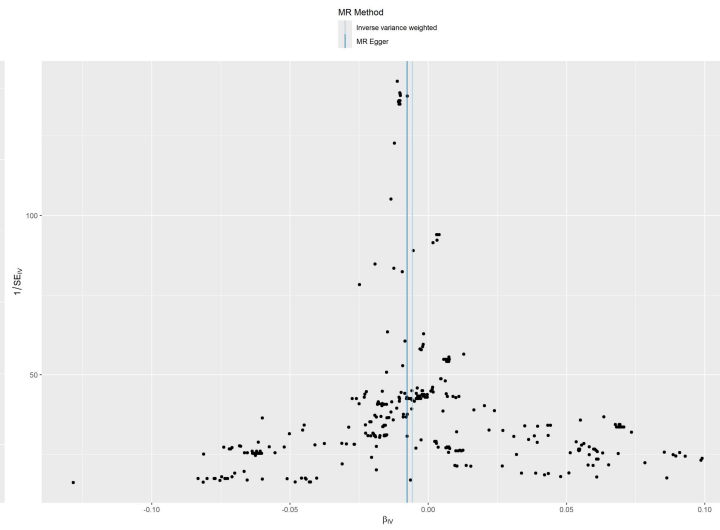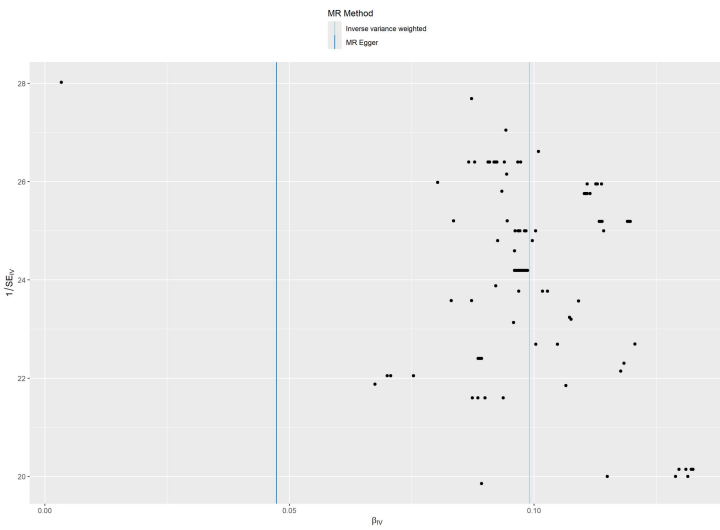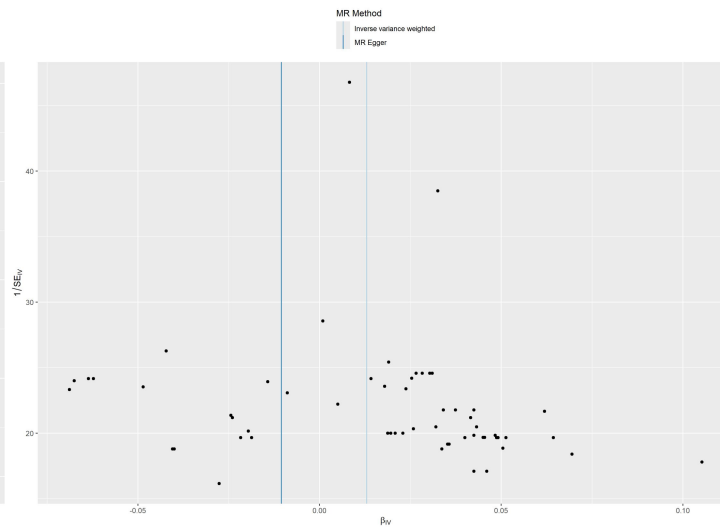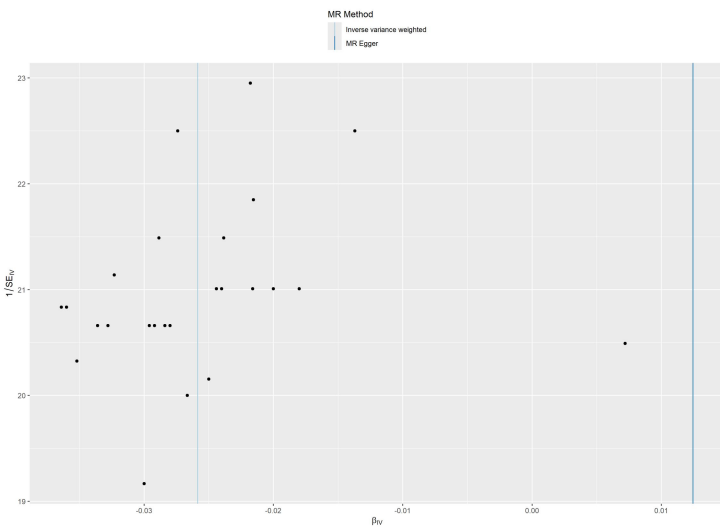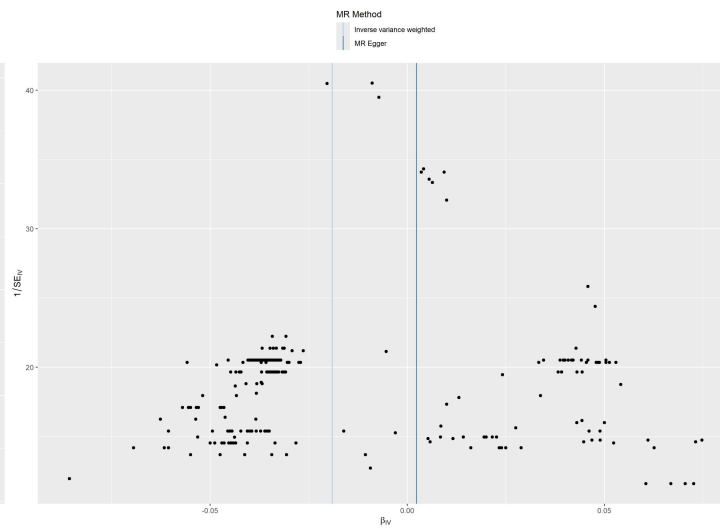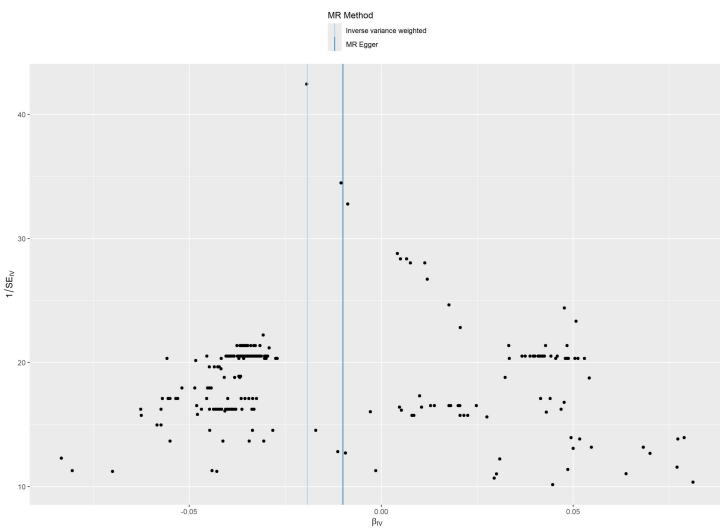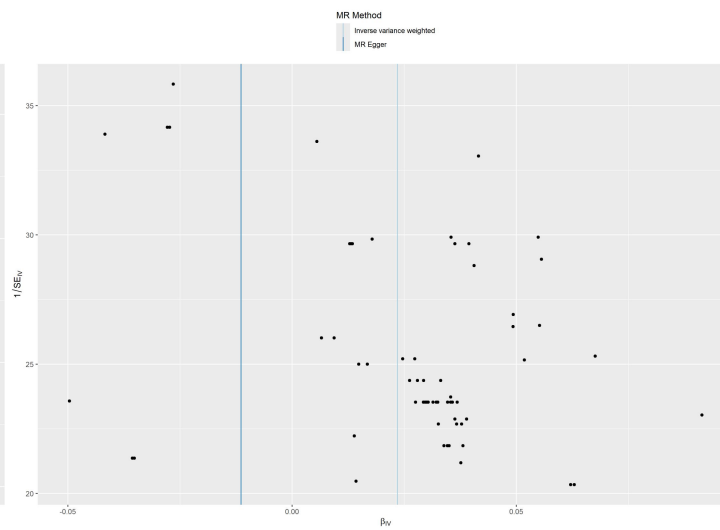

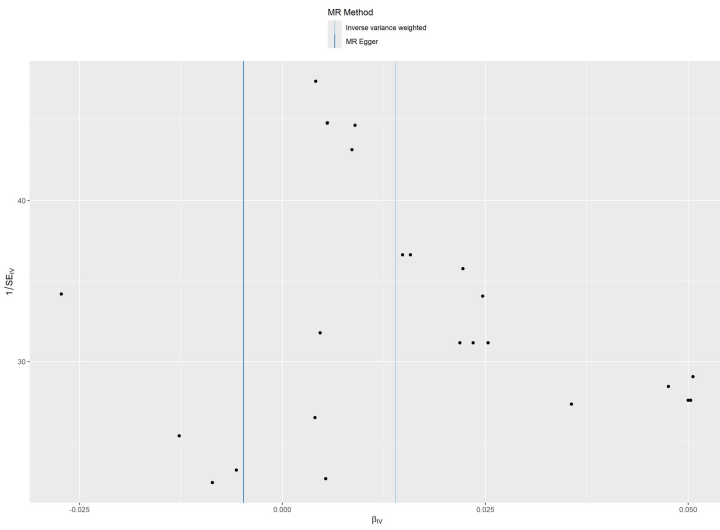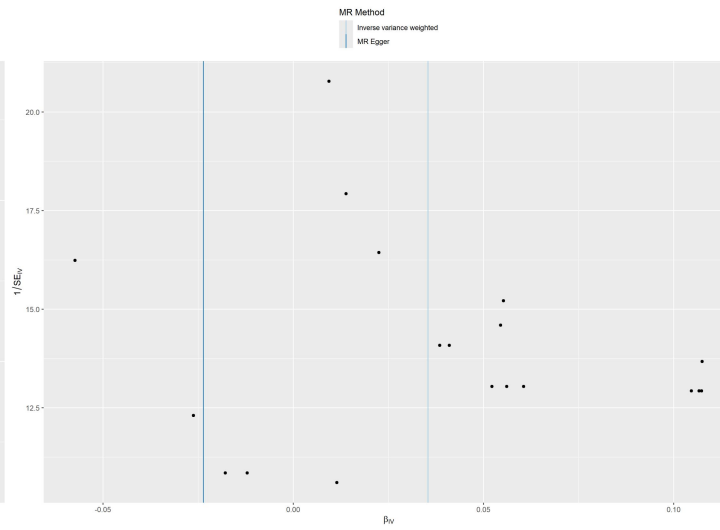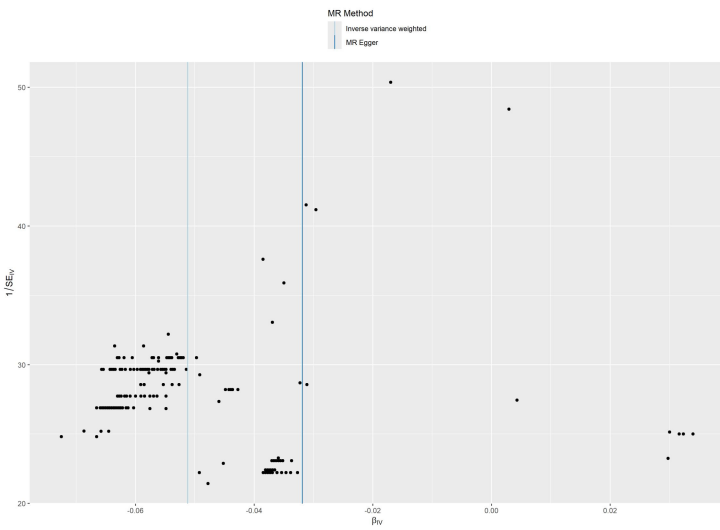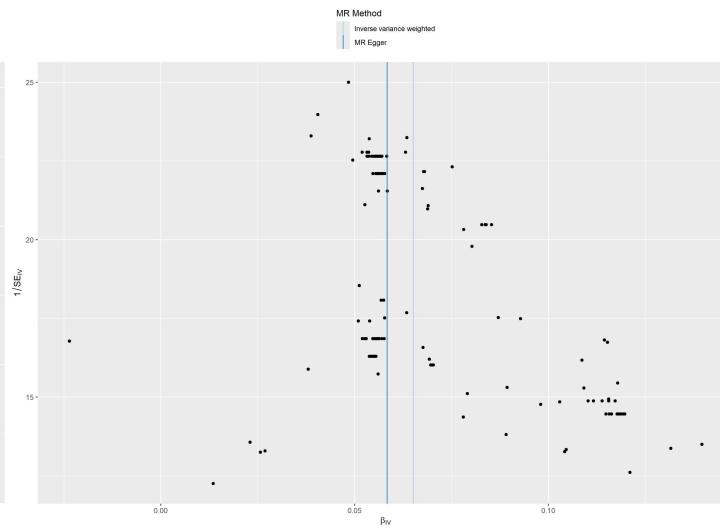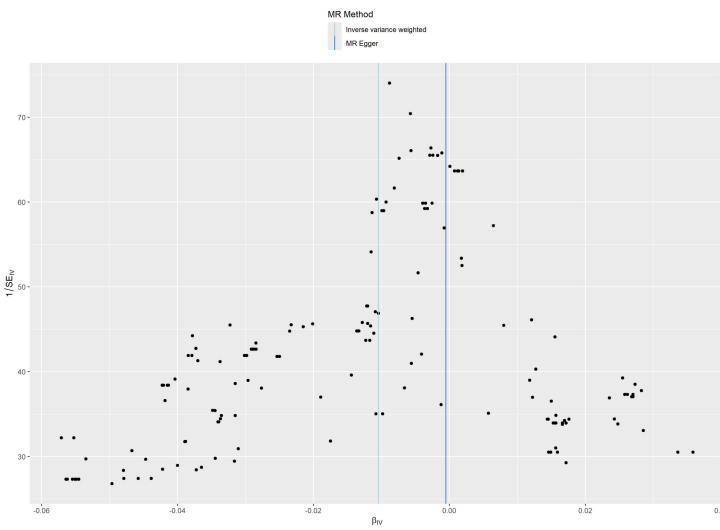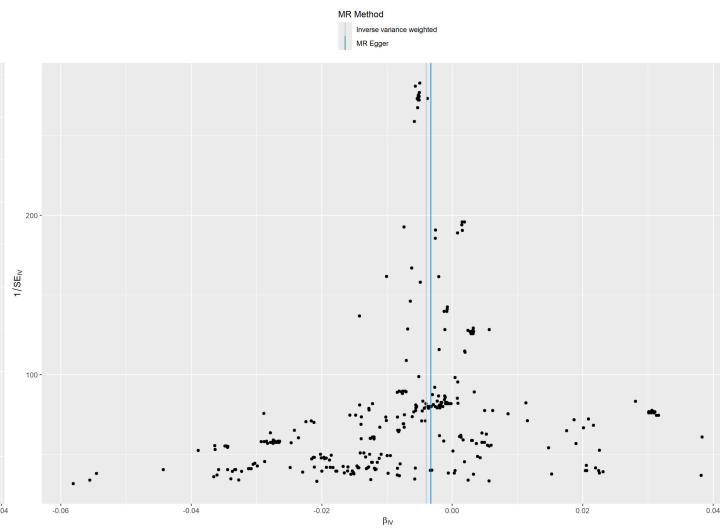

Supplement: Supplementary file 1 [file ijms-26-00283-s001.zip › Supplement File S4. hip OA funnel plots.pdf]

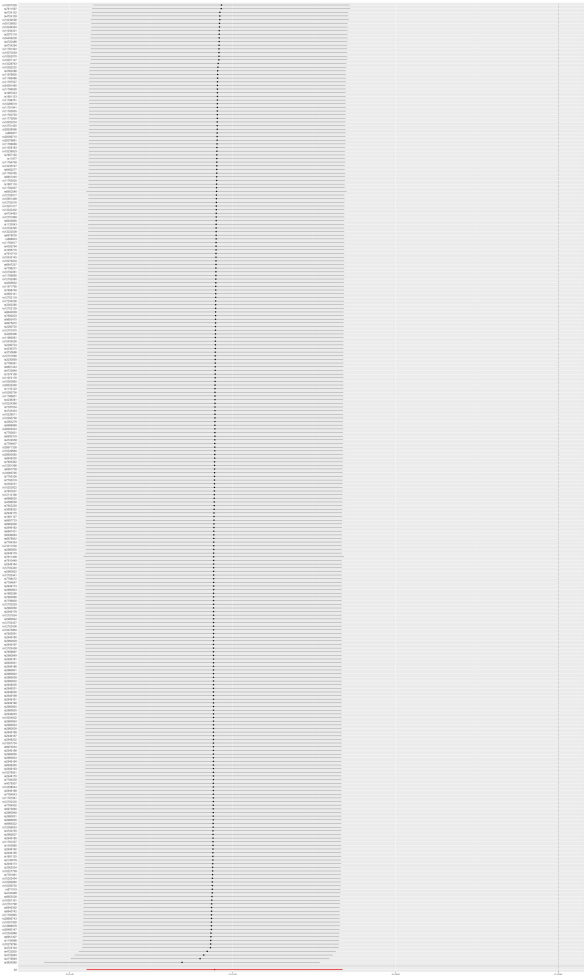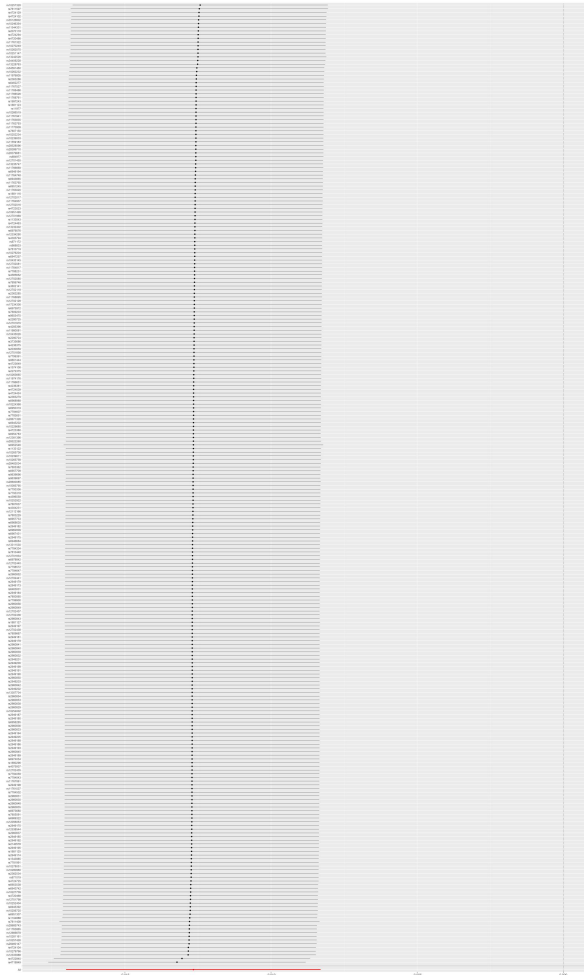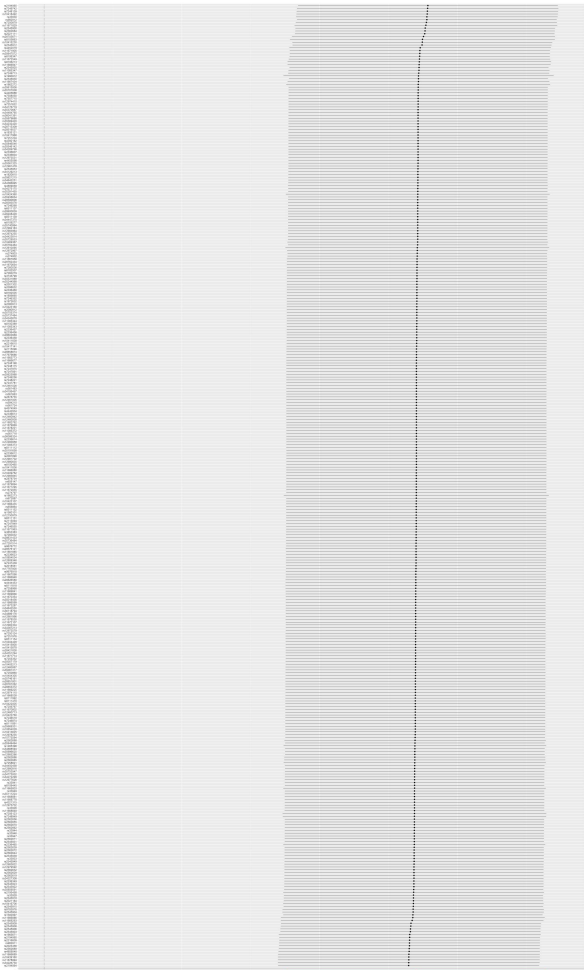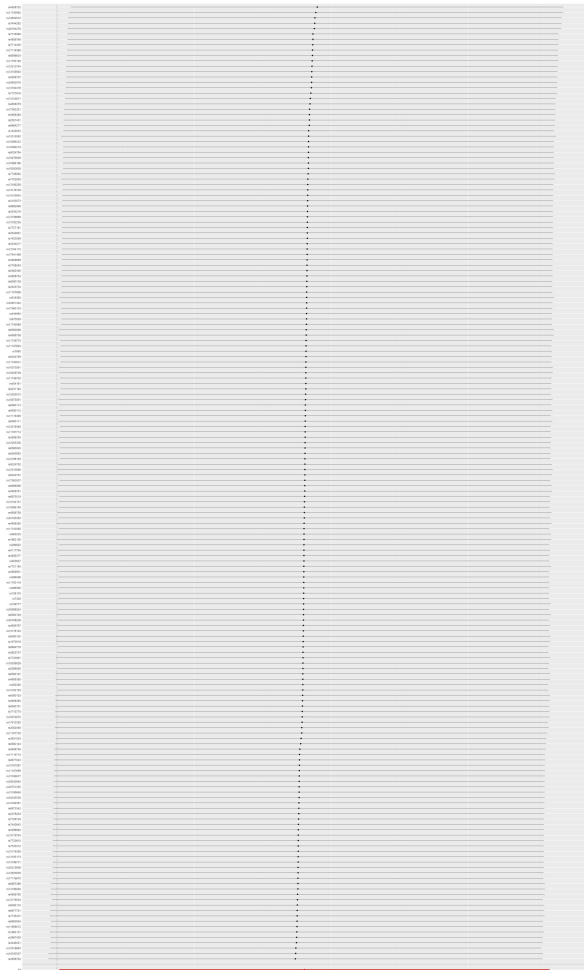

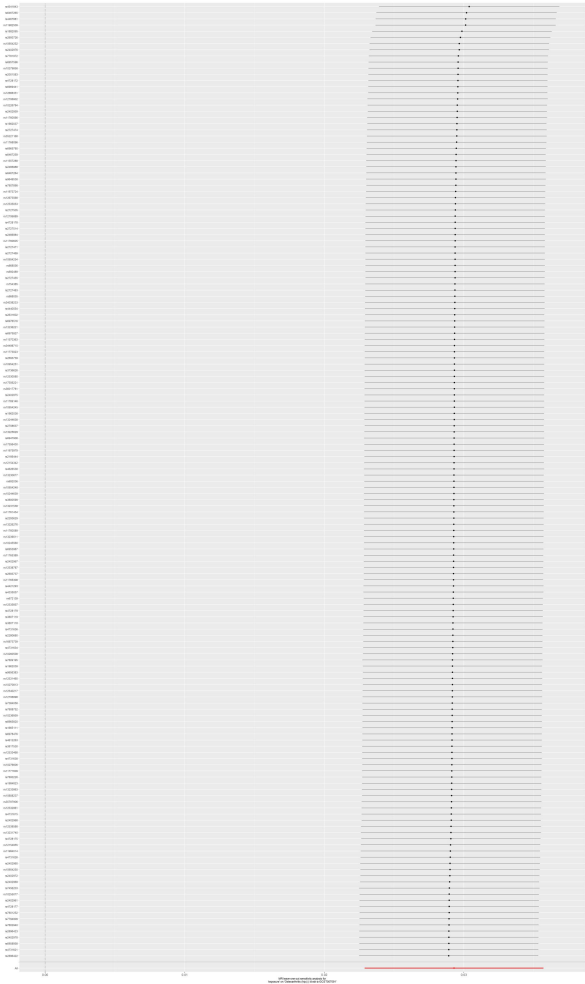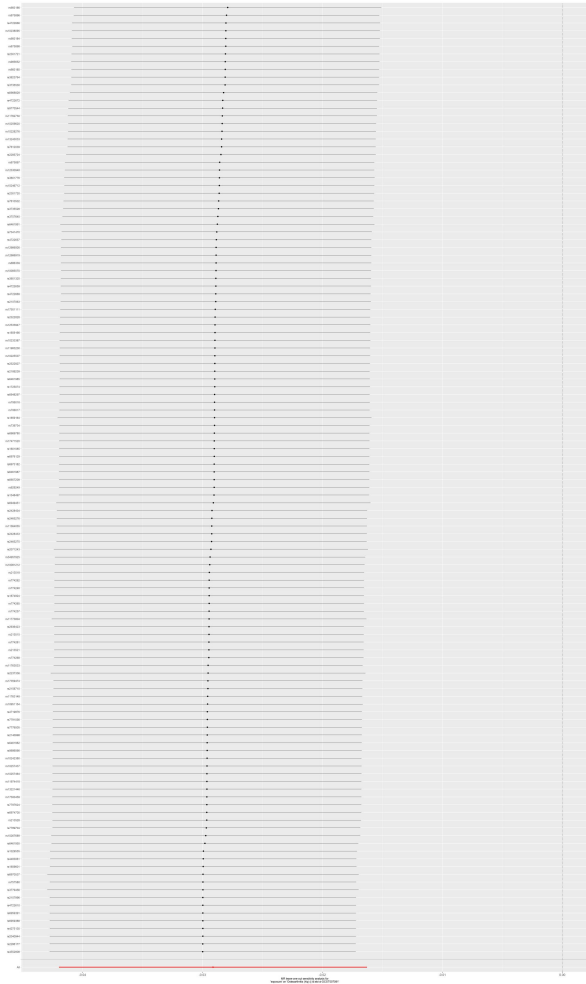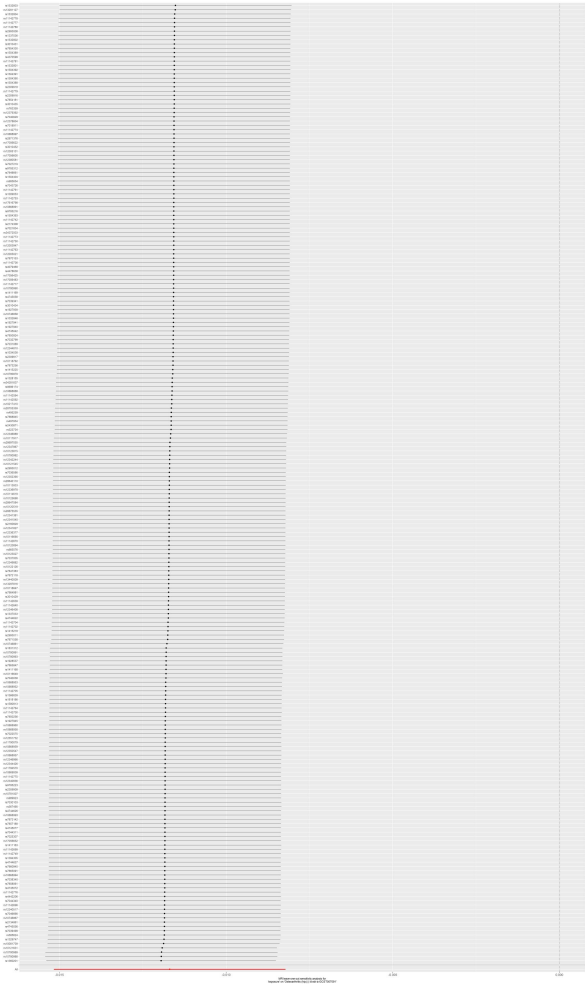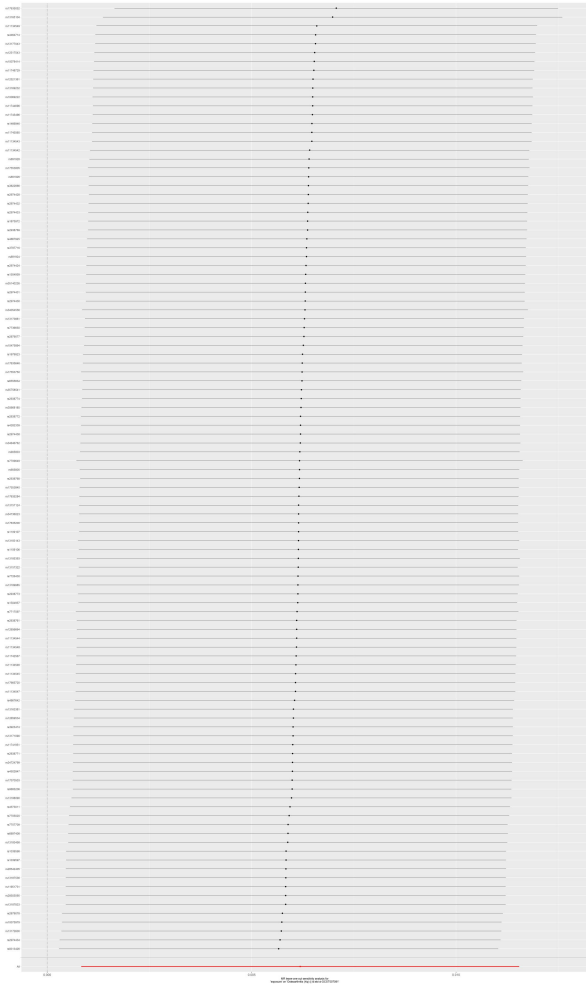

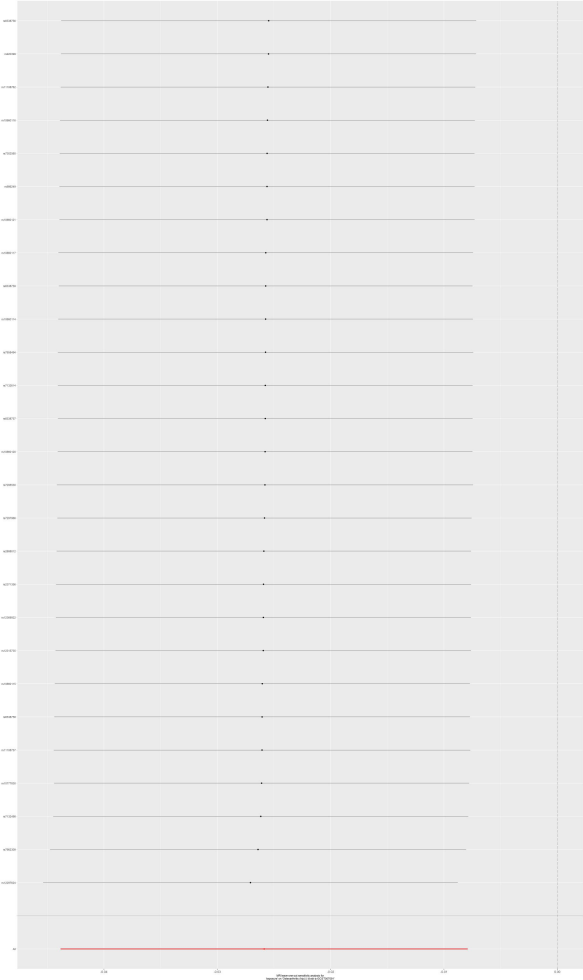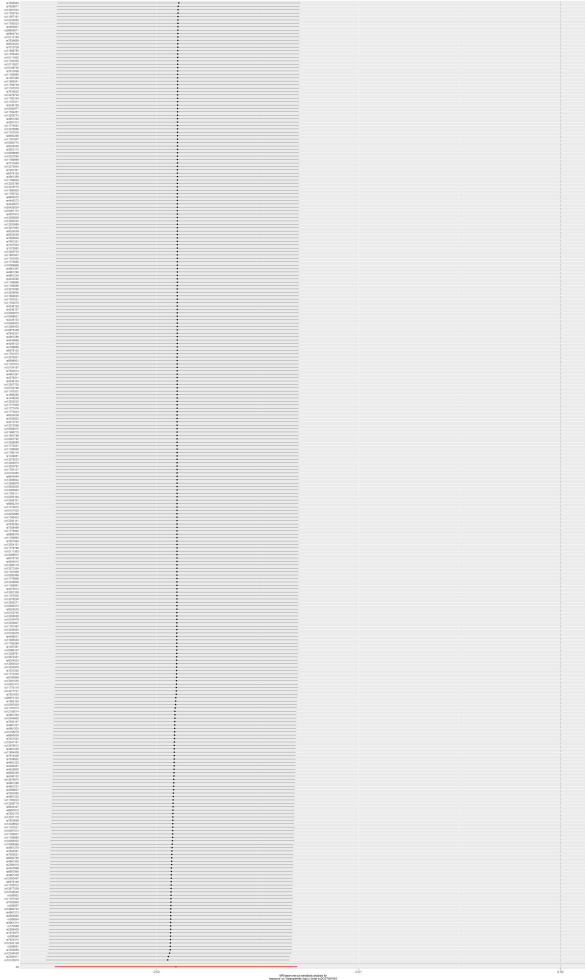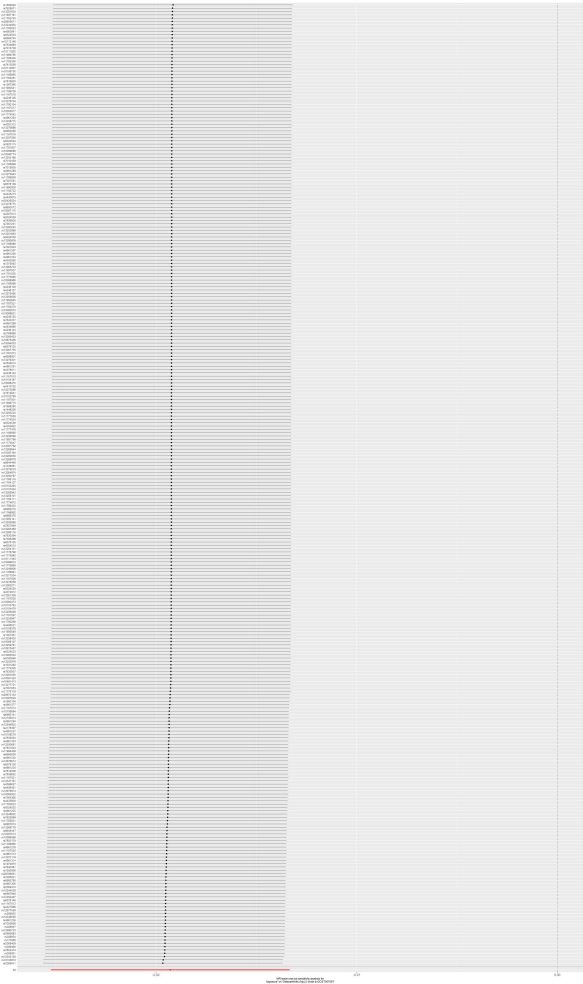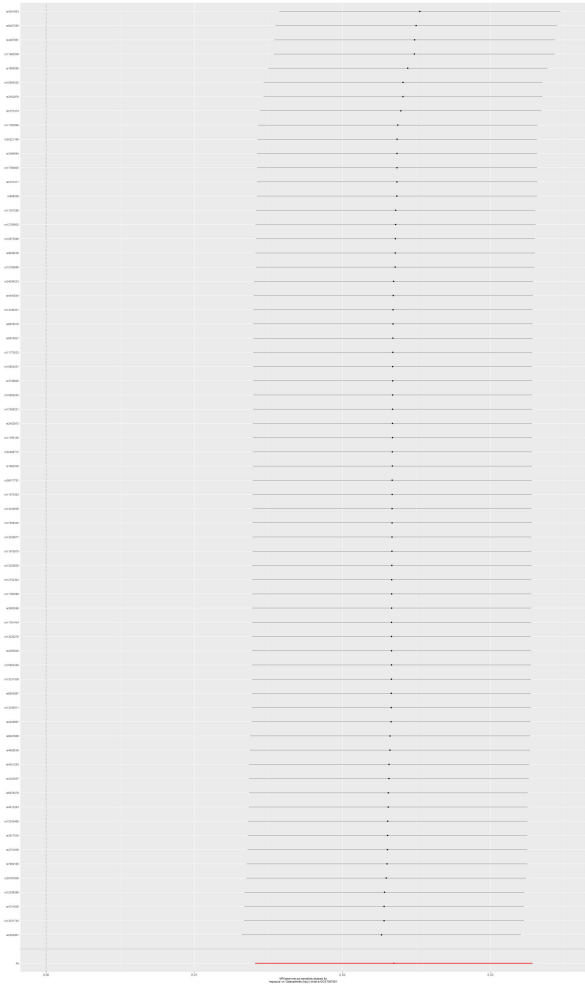

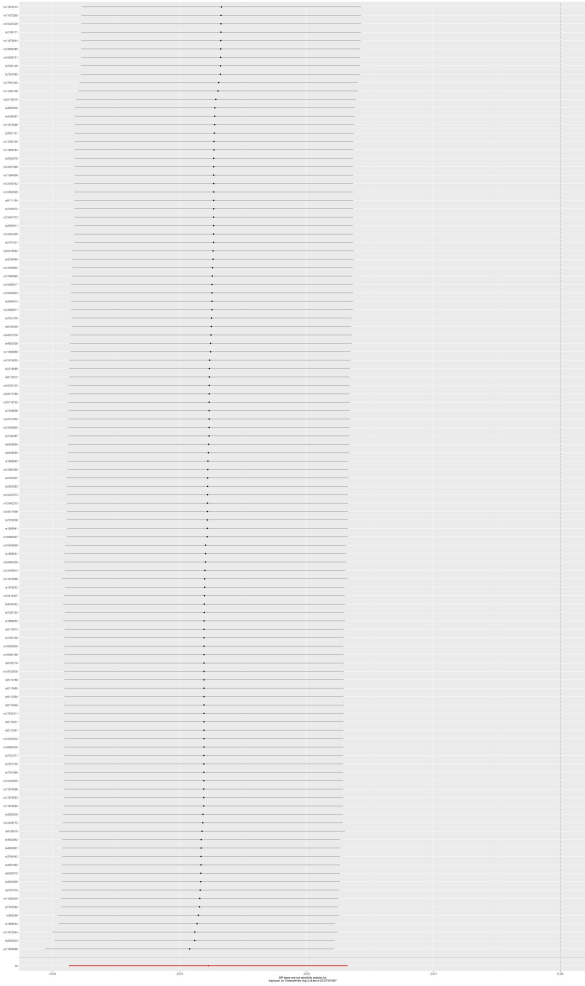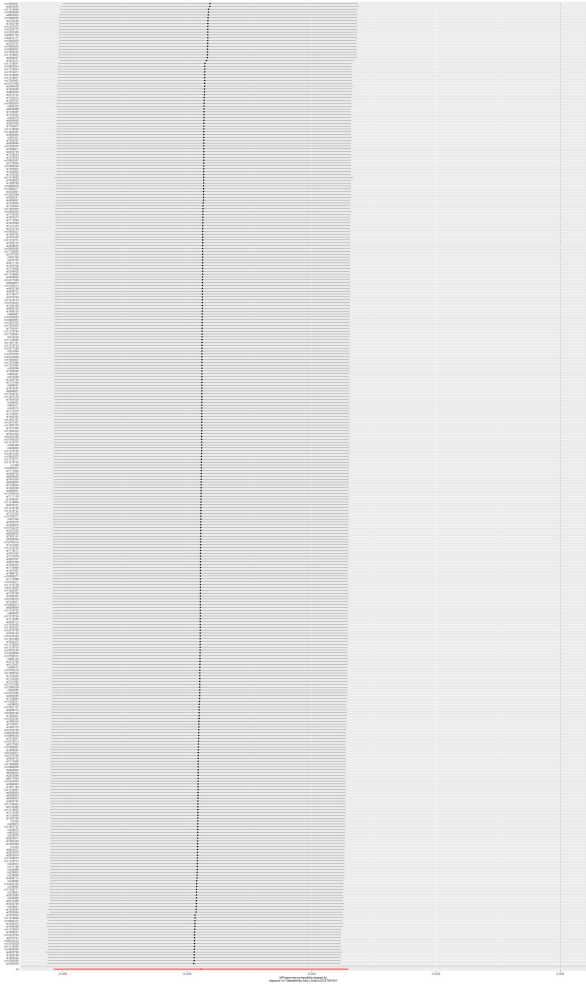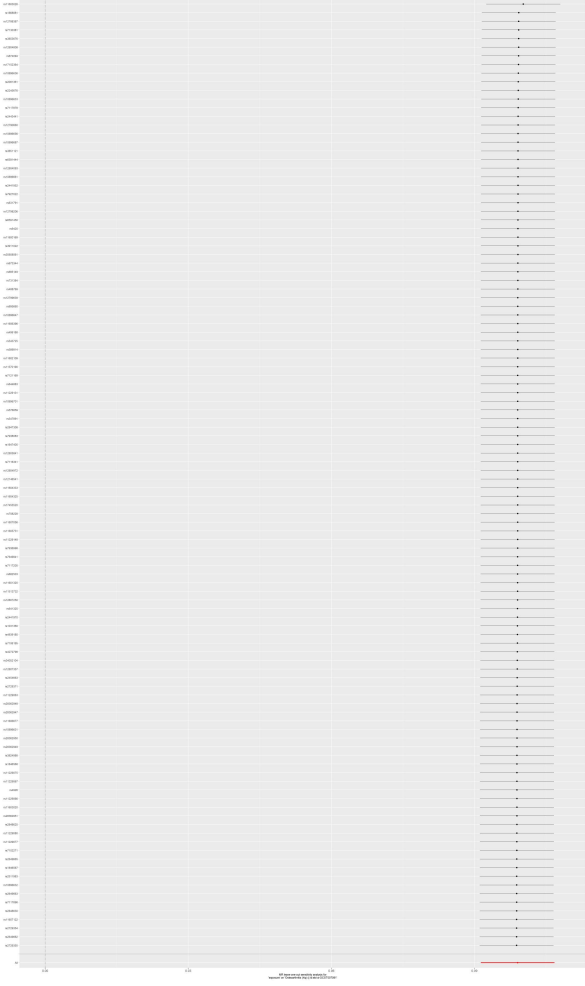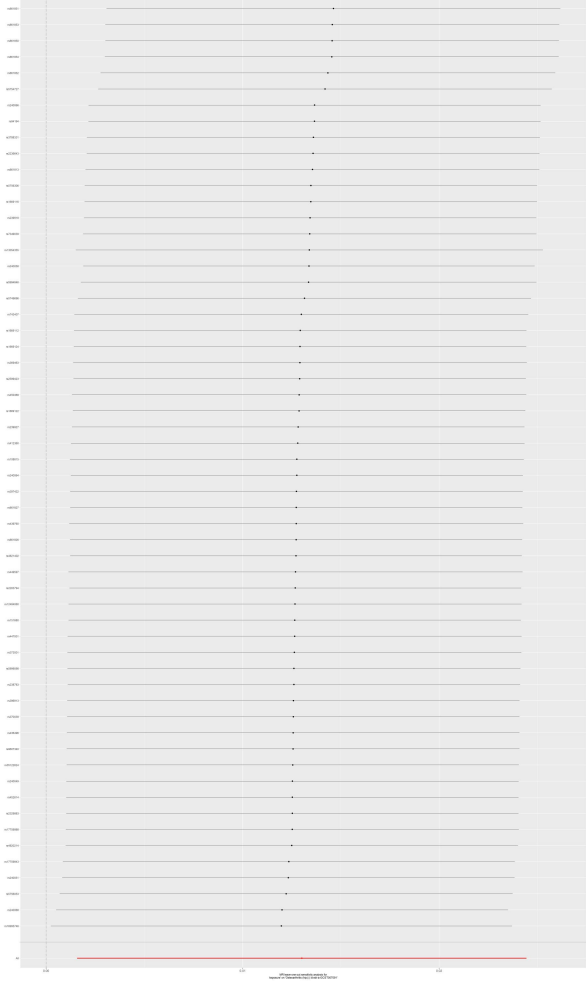

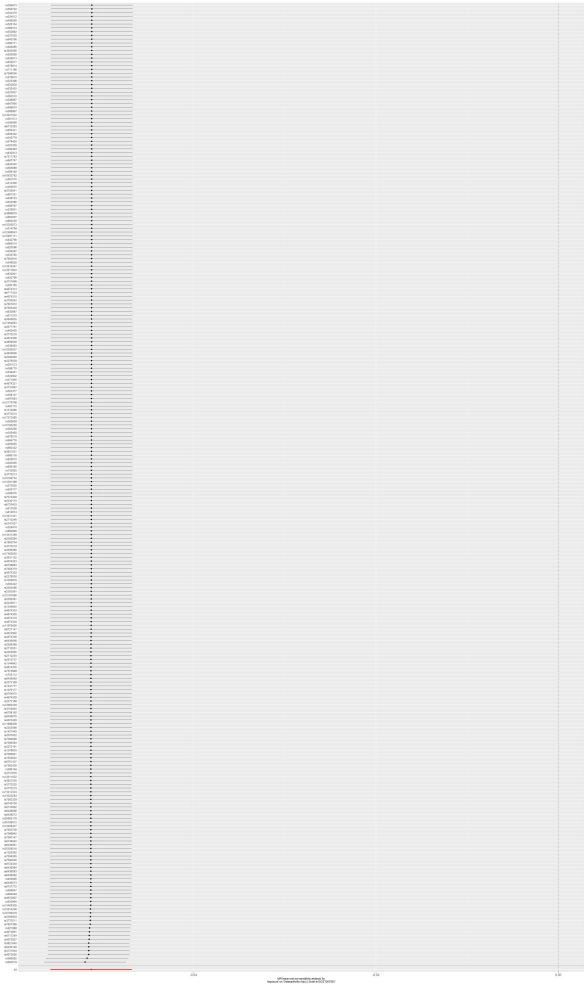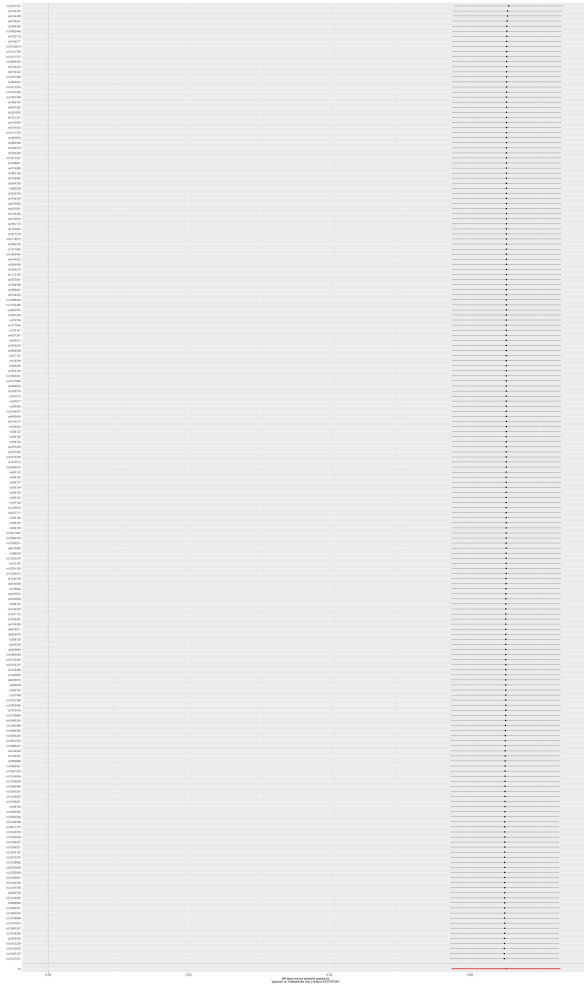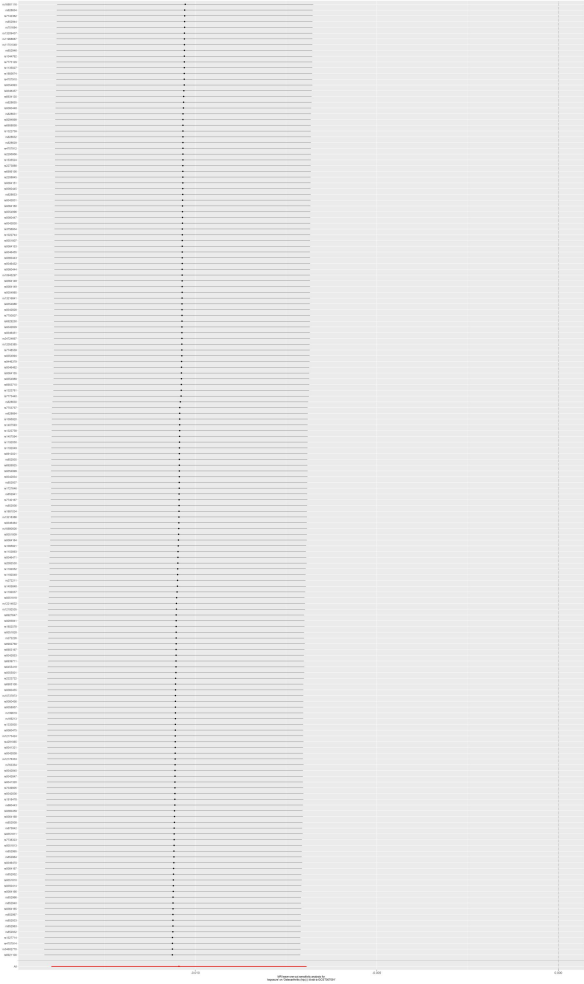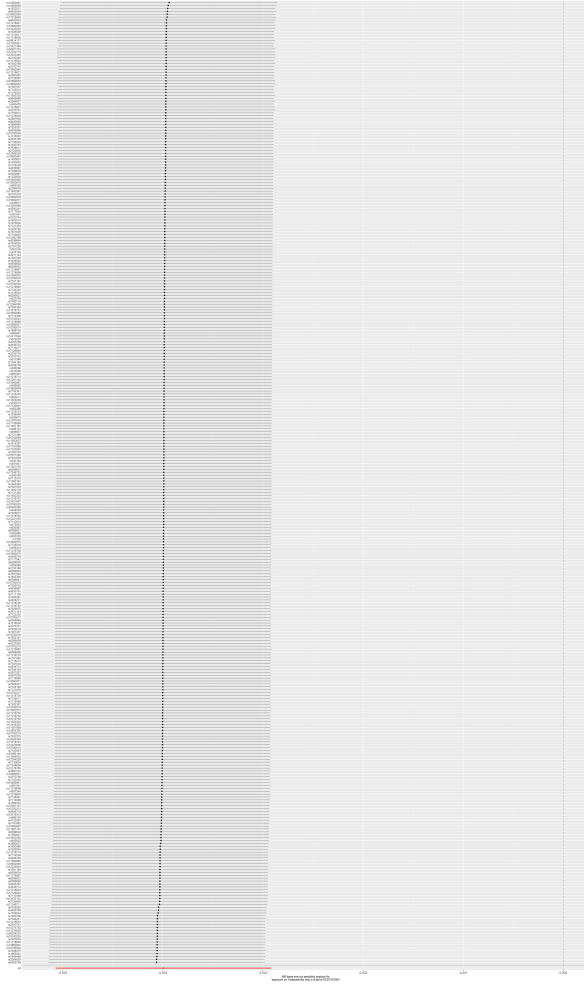

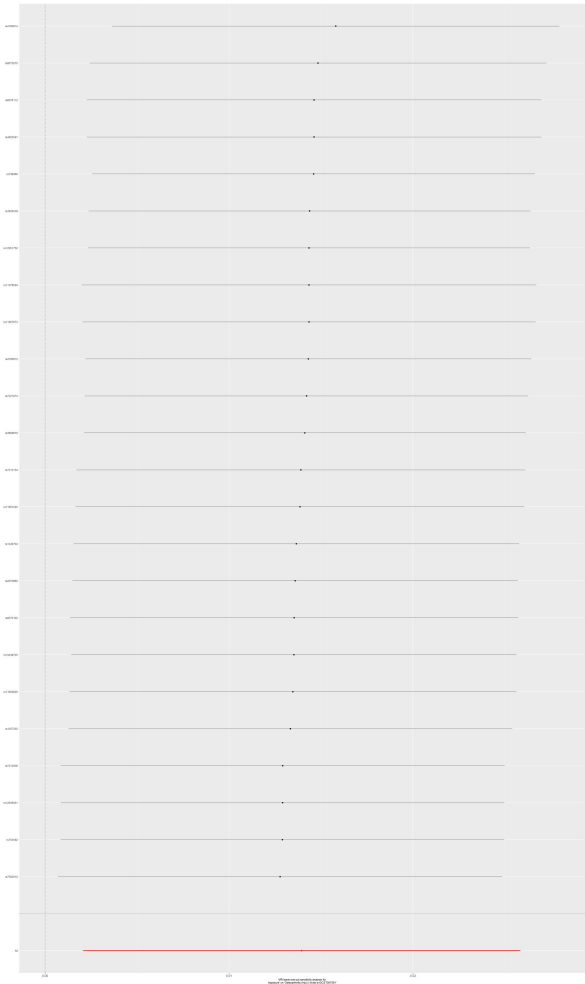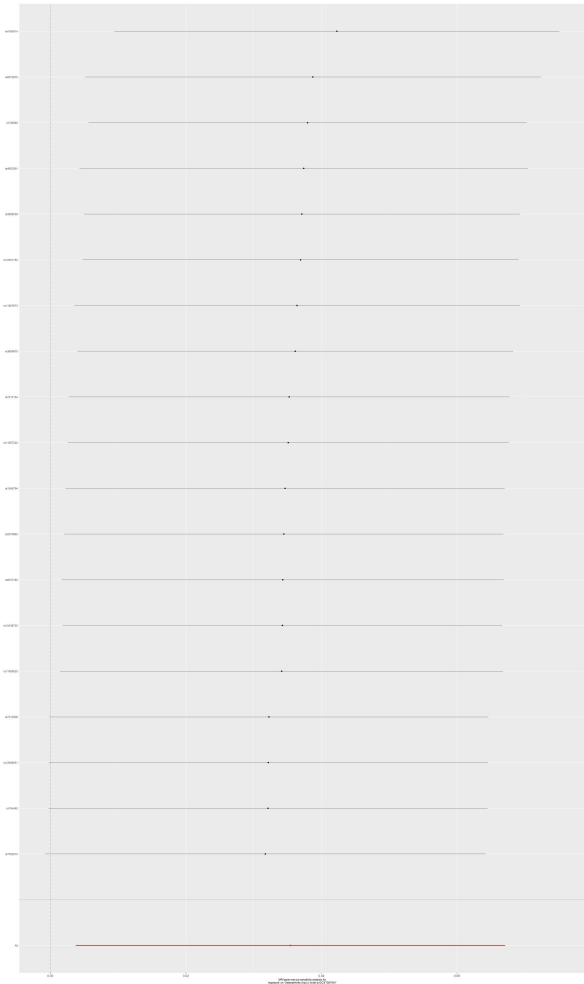

Supplement: Supplementary file 1 [file ijms-26-00283-s001.zip › Supplement File S5. hip OA leave-one-out forest maps.pdf]
